# Markers of Type 2 Inflammation and Immunosenescence Are Upregulated in Localized Scleroderma

**DOI:** 10.3390/ijms26031258

**Published:** 2025-01-31

**Authors:** Lauren Khoury, Connor Prosty, Stephanie Ghazal, Sofianne Gabrielli, Kathryn S. Torok, Mohammed Osman, Elvis Martinez-Jaramillo, Philippe Lefrançois, Elena Netchiporouk

**Affiliations:** 1 Faculty of Medicine, McGill University, Montreal, QC H3G 2M1, Canadaconnor.prosty@mail.mcgill.ca (C.P.); sofianne.gabrielli@mail.mcgill.ca (S.G.); 2 Division Dermatology, McGill University Health Centre, Montreal, QC H4A 3J1, Canada; stephanie.ghazal@mail.mcgill.ca (S.G.); elvis.martinez-jaramillo@mail.mcgill.ca (E.M.-J.); 3 Department of Pediatrics (Rheumatology), University of Pittsburgh, Pittsburgh, PA 15224, USA; kathryn.torok@chp.edu; 4 Division of Rheumatology, University of Alberta, Edmonton, AB T6G 2R3, Canada; mosman@ualberta.ca; 5 Division Dermatology, Jewish General Hospital, Montreal, QC H3T 1E2, Canada; philippe.lefrancois2@mcgill.ca

**Keywords:** immunology, inflammaging, morphea, pathogenesis, RNA sequencing, immunosenescence, autoimmunity, localized scleroderma

## Abstract

Localized scleroderma (LS) is an autoimmune, fibrotic skin disease that is thought to be triggered by environmental factors. Recent evidence from systemic autoimmune diseases proposed that the induction of immunosenescence may link environmental triggers with autoimmunity development. We aimed to explore the inflammatory signature in juvenile LS and investigate the presence of DNA instability and immunosenescence using publicly available transcriptomic data. High-throughput RNA sequencing data from 28 juvenile LS and 10 healthy controls were analyzed. Unsupervised clustering, pathway analyses, cell-type enrichment, fusion analyses, and immunosenescence gene set enrichment were performed. IFN and Type 1/2/3 pathways were upregulated in clinically active and histologically inflammatory LS. Type 2 inflammatory signature in both inflammatory and fibrotic LS was demonstrated by enriched genes, pathways, and deconvolution analyses (eosinophils). Features of genotoxic stress signals manifesting as DNA instability genes, pathways, and fusion events as well as mitochondrial dysfunction were demonstrated for the first time in LS. Features of immunosenescence (e.g., the upregulation of pathways involved in T cell exhaustion, inhibitory receptors, and cellular senescence and the enrichment of senescent genes) were also confirmed in (active and inflammatory) LS. Immunosenescence and inflammaging may underlie the complex and heterogeneous nature of immune responses seen in LS and should be further studied.

## 1. Introduction

Localized scleroderma (LS, also known as morphea) is a fibrotic skin disease with juvenile onset in up to one-third of cases [1]. The disease presents in various forms—circumscribed, linear, generalized, and mixed—which differ in their risk of permanent sequelae [2]. Non-circumscribed subtypes, particularly in pediatric patients, often lead to life-long disfigurement and functional impairments [2].

LS progresses through three distinct stages: initial inflammation, characterized by violaceous plaques with lymphoplasmacytic infiltration; fibrosis marked by dense dermal sclerosis; and eventual skin atrophy [1,3]. While the clinical course and histopathology are well-documented, the underlying pathogenesis remains poorly understood. Recent studies implicate dysregulated interferon (IFN) signaling and a potential shift from Type 1/3 to Type 2 immunity as drivers of disease progression [2,4,5]. However, the triggers for IFN release and its impact on disease stages require further investigation.

Current treatments primarily involve immunosuppressive agents like methotrexate and corticosteroids, which are limited by moderate efficacy and significant adverse effects [6]. As a result, there is an urgent need for targeted therapies based on a deeper understanding of LS’s immune mechanisms [3].

In this study, we leveraged high-throughput transcriptomic data to explore immune signatures in juvenile LS. We hypothesized that early inflammatory stages would exhibit Type 1/3 immune responses, transitioning to Type 2 responses in fibrosis. We also investigated the role of DNA instability and immunosenescence in disease pathogenesis. Our findings aim to elucidate novel molecular pathways and provide a rationale for repurposing existing therapies for LS treatment.

## 2. Results

### 2.1. Demographics and Clinical Characteristics

In this study, we analyzed 28 pediatric LS cases and 10 HCs (Table 1). LS cases were categorized into linear (n = 12), generalized (n = 8), and circumscribed (n = 8) subtypes. Two-thirds of the cases were active LS (mLoSSI or PGA-A of > 0). The mean mLoSSI was 7.6 ± 6 among active cases. Histologically, 12 samples were inflammatory, and dermal collagen thickness ranged from 6.20–41.20 μm.

### 2.2. Differentially Expressed Genes (DEGs), Clustering, Pathway Analyses, and Deconvolution

**Figure 1:**
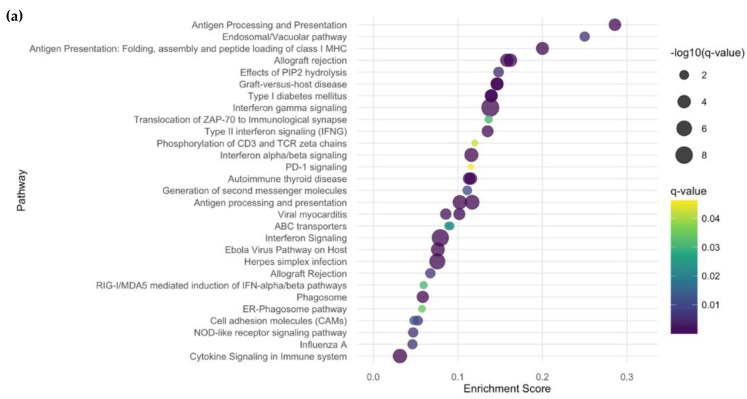

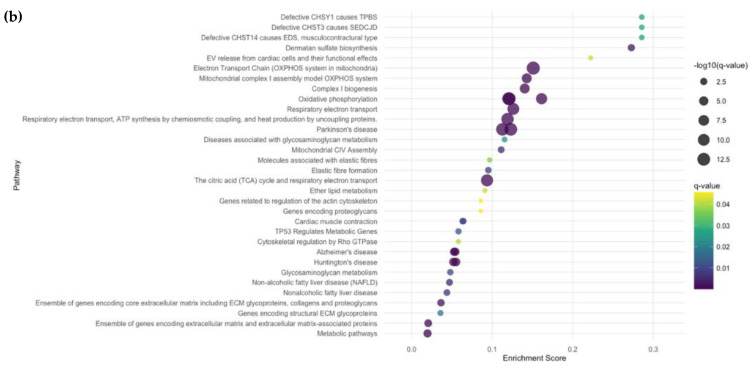
Bubble plot of dysregulated pathways in LS vs. HC. (**a**) Upregulated ToppGene Pathways in LS vs. HC. (**b**) Downregulated ToppGene Pathways in LS vs. HC. The enrichment plots illustrate pathways ranked by their biological significance, with the *x*-axis representing the enrichment score and the *y*-axis listing the pathways. Circle size corresponds to statistical significance, where larger circles indicate more significant pathways based on q-value. The color gradient, ranging from dark purple to yellow-green, reflects the q-value, with darker colors denoting higher significance. LS, localized scleroderma; HC, healthy controls.

We identified 173 upregulated and 160 downregulated DEGs when comparing LS lesional skin to HC (Tables S1–S3). Upregulated genes included those involved in Type 1 IFN signaling (e.g., *IRF7*, *IFI27*, *CIITA*), Type 2 immunity (e.g., *CORO1A*, *TNFRSF25*, *ANO1*), DNA instability/repair/oxidative response (e.g., *CPSF1*, *PARP10*, *FCHSD1*, *PABPC1L*), and immunosenescence (e.g., *DOT1L*, *TCOF1*) (Figure S1) [7,8]. Enriched pathways included Type 1/2 IFN responses, T-cell activation, Th1/Th2 differentiation, viral/autoimmune responses, and Programmed Death (PD)-1 signaling pathways (Figure 1a and Figure S2).

Conversely, downregulated genes were primarily linked to mitochondrial respiration (e.g., *MT-ND4L*, *MT-ATP8*), vasculogenesis (e.g., *LYVE1*, *CNN1*), autophagy (e.g., *DCN*), and hair follicle development (e.g., *LGR5*) (Table S3). ToppGene corroborated mitochondrial respiration, oxidative phosphorylation, neurodegenerative diseases, encoding extracellular matrix proteins, glycosaminoglycans, elastin, and TP53 as the most downregulated pathways (Figure 1b).

Using xCell deconvolution, eosinophil scores were significantly increased in LS lesional skin (Q = 1.28 × 10^−2^) (Figure S3). However, CIBERSORT analysis did not show distinct cell-type enrichment between LS and HC samples.

#### 2.2.1. Subgroup Analyses: Linear, Circumscribed, Generalized LS vs. HC

The unsupervised hierarchical clustering illustrated three clusters. The first two clusters included LS patients without predilection for a specific subtype, and the third cluster included 10 HCs and 3 LS samples (Figure S4). DEGs, pathway analyses, xCell, and CIBERSORT were performed separately for each subtype and compared to HC. Similar trends as those previously mentioned were found.

#### 2.2.2. Subgroup Analyses: Active vs. Inactive LS

In total, 276 genes were upregulated and 246 downregulated in active LS (n = 19) vs. HC (n = 10) samples (Table S1). Unsupervised hierarchical clustering based on the top 50 upregulated genes revealed clear differentiation, with one cluster of 13 active LS cases and mixed clusters of active/inactive samples (Figure S5).

Unique to active LS (n = 19) were genes involved in B-cell immunity (e.g., IGLC2, IGKC, ITGAL) (Table S4). As we expected, the exclusion of inactive LS samples revealed more prominent immune-mediated pathways among the subset of active LS. Notably, pathways enriched in active LS included Type 1/2/3 IFN signaling, T-cell differentiation/activation, viral illnesses, and autoimmune responses, alongside Th1/Th2/Th17 and JAK-STAT pathways, suggesting broad immune activation (Figures S6–S9). Additionally, the immunosenescence pathways associated with immune exhaustion (e.g., PD-1, CTLA-4, cellular senescence) and pathways in cancer were also upregulated.

Downregulated pathways were largely related to mitochondrial function, gene expression regulation, oxidative stress response, autophagy, TP53 signaling, and neurodegeneration. The upregulated (Figures S6 and S7) and downregulated (Figures S8 and S9) pathways presented with ToppGene and KEGG are summarized. xCell analysis further confirmed the enrichment of Th1 cells (Q = 2.08 × 10^−2^) and eosinophils (Q = 2.05 × 10^−2^) in active LS (Figure S10), while CIBERSORT did not detect significant cell-type differences.

#### 2.2.3. Subgroup Analyses: Inflammatory vs. HC

**Figure 2:**
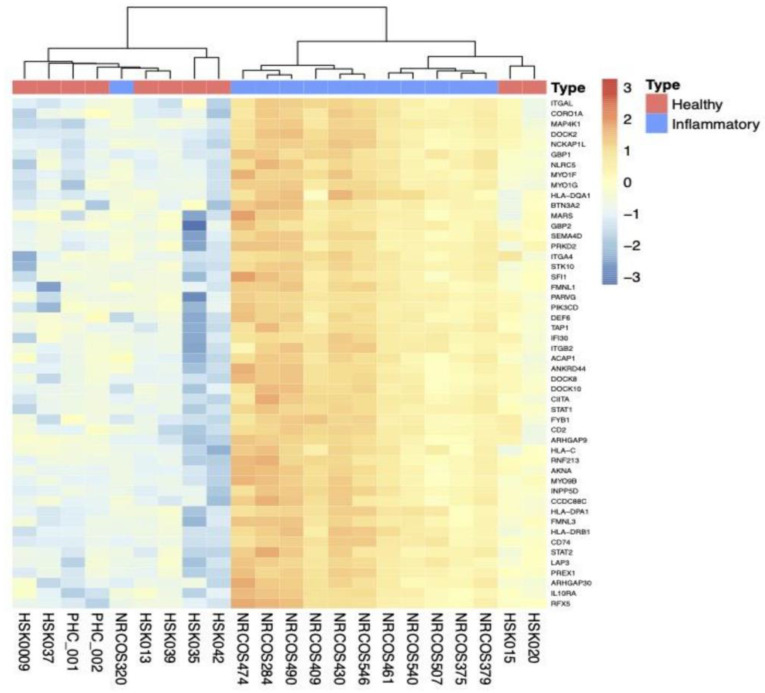
Unsupervised hierarchical clustering based on top 50 genes in inflammatory LS vs. HC. The color key indicates gene expression as a z-score. Inflammatory LS samples (n = 12) are indicated by blue color, HC (n = 10) by red. Two clusters were identified, the first consisting of 8 HC/1 LS samples and the second 11 LS/2 HC. LS, localized scleroderma; HC, healthy controls.

As demonstrated in the heat map of active LS (Figure S11), some cases clustered with HCs which may have stemmed from similar signals between mildly active (resolving) and inactive LS. Hence, we performed a subgroup analysis focusing on the differences between inflammatory LS samples (n = 12) vs. HC (n = 10). Unsupervised hierarchical clustering revealed two clusters. The first included 8 HC/1 LS samples and the second 11 LS/2 HC samples, suggesting a better separation between inflammatory vs. HC than active vs. HC (Figure 2). A total of 109 genes were upregulated, and 148 genes were downregulated (Table S1). The top 50 upregulated and downregulated genes (Tables S6 and S7) and ToppGene and KEGG pathways analyses (Figures S12 and S13) are summarized.

In addition to the upregulated pathways seen in active LS, Th2 signals such as IL-3, IL-4, IL-5, IL-9, IL-13, and TSLP signaling pathways were upregulated, indicating strong Th2 skewing. DNA damage, immunosenescence, and pathways in cancer were prominent. Unlike other comparison groups, the upregulation of pathways involved in platelet activation and hemostasis were noted. There were no significantly downregulated pathways using pathway analyses.

Cell abundance scores (xCell) determined that only eosinophils were increased in inflammatory skin compared to HC skin (Q = 3.52 × 10^−2^) (Figure S14). CIBERSORT analysis did not detect any differences in cell-type enrichment between LS and HC skin samples.

#### 2.2.4. Subgroup Analyses: Fibrotic vs. HC

**Figure 3:**
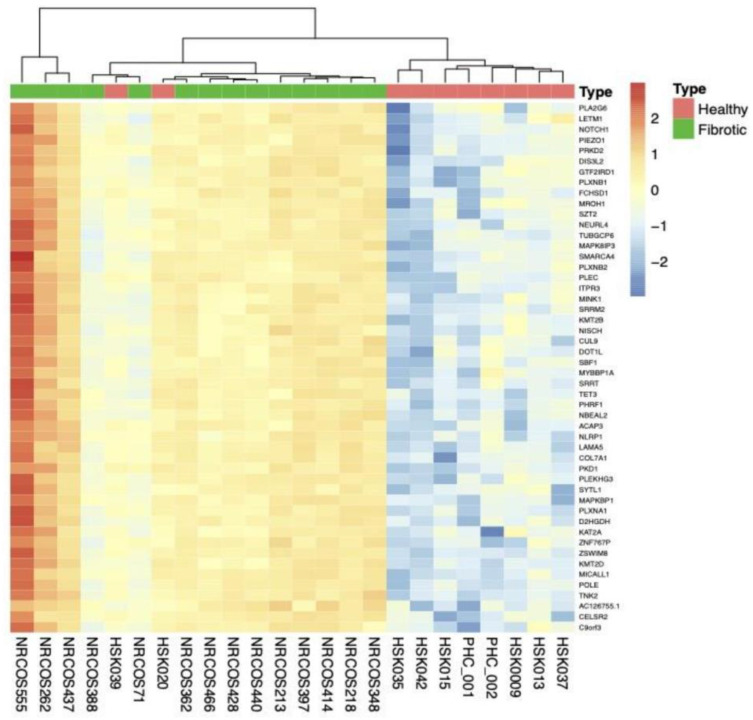
Unsupervised hierarchical clustering based on top 50 genes in fibrotic LS vs. HC. The color key indicates gene expression as a z-score. Fibrotic LS samples (n = 14) are indicated by green color, HC (n = 10) by red. Three clusters were identified, the first consisting of 3 fibrotic LS, the second of 2 HC/11 LS, and the latter including 8 HC. LS, localized scleroderma; HC, healthy controls.

Analyses were also performed for fibrotic LS samples (n = 14) vs. HC (n = 10). Unsupervised hierarchical clustering revealed three clusters, the first consisting of 3 fibrotic LS, the second of 11 fibrotic LS and 2 HC, and the latter including 8 HC (Figure 3).

A total of 160 genes were upregulated, and 225 genes were downregulated (Table S1). Most upregulated genes were involved in the epidermal structure (e.g., *KRT1*, *FLG*, *KRT5*, *KRT10*, *KRT 14*, and *COL17A1*), and extracellular matrix production (e.g., *COL7A1*, *CLCA2*, and *COL27A1*) (Table S8). The most downregulated genes were *AMY1B* (Q = 1.10 × 10^−2^) and *AMY2A* (Q = 2.58 × 10^−2^), along with mitochondrial genes such as *MT-ATP8* (Q = 3.98 × 10^−2^) and *MT-ND4L* (Q = 2.59 × 10^−2^) (Table S9). Most upregulated pathways involved collagen biosynthesis/formation, extracellular matrix organization, keratinization, and angiogenesis (Figures S15 and S16). Downregulated pathways were similar to LS in general and were involved in mitochondrial oxidation, respiration, and neuroinflammation (Figure S17). There were no downregulated pathways in the KEGG analysis.

Five cell types were enriched in fibrotic LS compared to HC skin: CD4+ central memory T cells (Q = 2.88 × 10^−3^), eosinophils (Q = 2.88 × 10^−3^), epithelial cells (Q = 3.68 × 10^−3^), keratinocytes (Q = 3.68 × 10^−3^), sebocytes (Q = 5.12 × 10^−3^), and central dendritic cells (Q = 3.52 × 10^−2^) (Figure S18). RNA deconvolution did not detect any differences in cell-type enrichment between LS and HC skin samples.

### 2.3. Gene Set Enrichment Analysis (GSEA) of Immunosenescence-Associated Genes

**Figure 4:**
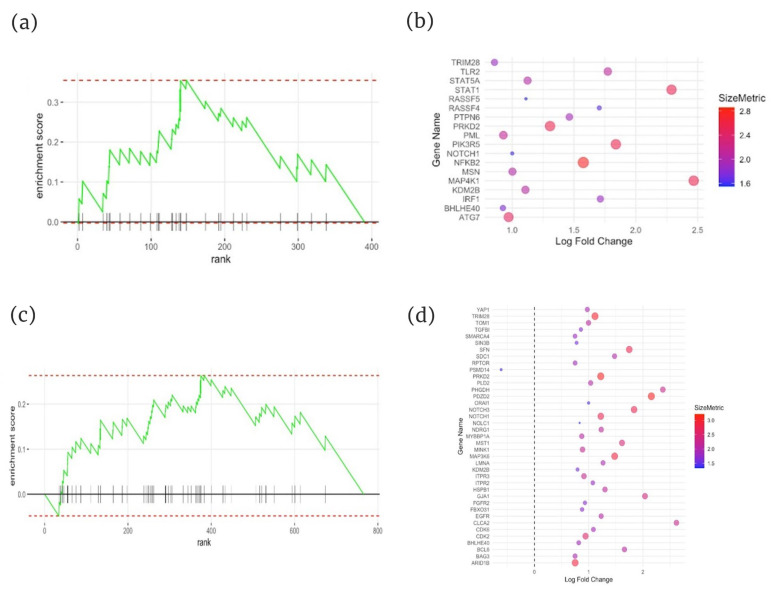
Distribution of leading-edge genes from GSEA comparing inflammatory LS and fibrotic LS vs. HC based on logFC and significance. (**a**) Enrichment score distribution for senescence-associated genes in inflammatory LS. (**b**) Gene influence bubble plot comparing inflammatory LS vs. HC, utilizing the CellAge Senescence Database. (**c**) Enrichment score distribution for senescence-associated genes in fibrotic LS. (**d**) Gene influence bubble plot comparing fibrotic LS vs. HC, referenced to CellAge Senescence Database. LS, localized scleroderma; HC, healthy controls.

Gene Set Enrichment Analysis (GSEA) was performed to investigate the enrichment of the senescence pathway within the dataset using the CellAge version 3 database. Inflammatory LS (n = 12, Q = 0.02) and fibrotic LS (n = 14, Q = 0.04) were significantly enriched with a normalized enrichment score (NES) of 1.76 and 1.56, indicating a moderate–strong enrichment (Figure 4a–d).

### 2.4. Fusion Transcript Analysis

A higher number of fusion events were observed in both active and inactive LS patients compared to HC. Specifically, a mean of 282.10 fusion events per sample were identified in active LS (n = 19) and 245.10 events per sample in inactive LS (n = 9), while HC had a mean of 189.60 fusion events per sample (Table S10). Only one HC (1/10) sample and six LS samples (6/28) demonstrated the same fusion genes identified by both STAR-fusion and Arriba (Table S11).

## 3. Materials and Methods

### 3.1. Data Sources

The Gene Expression Omnibus (GEO), a publicly available repository, was searched for high-throughput RNA-sequencing transcriptomic data in LS [9]. Two datasets were identified: GSE166863 (28 LS patients and 10 healthy controls [HC]) and GSE166861 (14 LS patients and 4 HC) [2,10]. GSE166863 included all of the GSE166861 sample raw data with batch correction. RNA-seq was performed on formalin-fixed paraffin-embedded (FFPE) samples with a %DV200 of at least 30% (range 30–78%) using Illumina NextSeq 500, and FastQ files were made using Illumina bcl2fastq2 [2]. The normalized reads per kilobase million (RPKM) from GSE166863 were downloaded from the GEO query package using the getGEO function, and linear models were fitted to each gene using Limma-Voom [11]. To increase the specificity, a union of Limma-Voom and EdgeR was used for inflammatory and fibrotic LS sample sets [11,12].

All demographic and clinical data were retrieved and supplemented by data provided by the authors (K.T.) in the case of missing or incomplete data [2,10]. LS subtypes were classified according to the Padua system, recognized as the gold standard for defining LS disease types [10,13]. Active LS (i.e., not atrophic or burned out LS) was defined as Physician Global Assessment of Activity (PGA-A) and modified Localized Scleroderma Skin Severity Index (mLoSSI) scores of > 0 [10]. Histological assessment categorized specimens into inflammatory or fibrotic with subcategories based on the degree of inflammation and fibrosis, as previously described [2].

### 3.2. Comparisons

LS was compared to HCs. LS subgroups based on disease subtypes (circumscribed, generalized, linear head, linear trunk/extremities), disease activity status (active vs. inactive), and histologic classification (inflammatory, fibrotic, normal-like) were compared to HC.

### 3.3. Differentially Expressed Genes (DEGs), Violin Plots, Unsupervised Clustering, Pathway Analyses, Gene Set Enrichment Analysis (GSEA)

DEGs were defined as genes with ≥2-fold change in expression (|logFc| ≥ 1) and a multiple hypothesis testing–accounting false discovery rate (Q-value) < 0.05 using the Benjamini–Hochberg method [14]. The functional annotation of DEGs was assessed using the Database for Annotation, Visualization, and Integrated Discovery (DAVID), and Violin plots were used to present the distribution of DEGs of interest [15,16]. Unsupervised hierarchical clustering was performed for the top 50 DEGs using the pheatmap package [17]. Pathway analyses were performed using ToppGene and KEGG according to all upregulated and downregulated genes [18,19]. GSEA with ‘fgsea’ R package was used to compare the enrichment of senescence-related genes across all LS groups using CellAge version 3 “https://genomics.senescence.info/cells/ (accessed on 01 May 2024)”, a manually curated database of 1259 gene expression changes associated with cellular senescence [20,21].

### 3.4. Cell Type Estimation

RNA deconvolution was performed with CIBERSORT using the standard leukocyte gene signature matrix (LM22) to estimate the relative proportions of 22 immune cell types [11]. Cell abundance scores were determined by xCell, using the standard 64-cell type signature [22]. CIBERSORT and xCell data were analyzed by bootstrapping using 100,000 iterations [11,22]. *p*-values were corrected by multiple hypothesis testing to give the Q separately in each of the xCell and CIBERSORT comparisons via the Benjamini–Hochberg method [14]. Box plots were used to illustrate individual cell enrichment [23].

### 3.5. Identification of Fusion Transcripts

To identify potential fusion transcripts, gene fusion analysis was conducted using STAR-Fusion and Arriba [24,25]. Fusion transcripts, hybrid RNA molecules formed from two previously separate genes, result from genomic rearrangements and indicate genomic instability. To avoid false positives, only the fusion genes selected by both methods were identified as significant. Quality control was performed using Picard tools, RNASeq2, RSeQC, and MultiQC [26,27,28].

### 3.6. Statistical Analyses

*p*-values and *Q*-values less than 0.05 were considered significant. All statistical tests were performed on R version 4.0.4 (R Core Team, Vienna, Austria), and graphs were plotted using the Ggplot2 package [23].

## 4. Discussion

LS is an autoimmune fibrotic disorder with a complex and poorly understood etiology [3]. While a rare pathogenic *STAT4* variant was implicated in severe juvenile cases, particularly pansclerotic LS, the majority of LS cases are thought to be driven by environmental factors in genetically predisposed individuals [29,30,31]. External triggers, including radiation, immunotherapy, occupational exposures, and infections, are associated with LS onset [32,33,34,35,36].

Our study aimed to elucidate the immune landscape of pediatric LS using RNA-seq analysis of lesional skin compared to HCs. Our findings demonstrate that while there are numerous transcriptional changes between LS and HC regardless of the clinical subtype, these changes are more pronounced in the clinically active and histologically inflammatory LS. IFN and Type 1/2/3 immune responses, as seen in active/inflammatory LS, mirror early systemic sclerosis (SSc) [37]. Type 2 inflammatory signature in both inflammatory and fibrotic LS as demonstrated by enriched genes, pathways, and deconvolution analyses (eosinophils) is novel. Besides Type 2 signal, fibrotic LS was pauci-inflammatory with prominent upregulation of genes and pathways involved in epidermal structure and extracellular matrix remodeling as seen in established SSc [37]. In contrast to SSc, the upregulation of genes and pathways involved in humoral immunity and vasculogenesis was scarce and only seen in inflammatory LS [37]. Taken together, this may suggest that although upstream inflammatory changes may not be the same between SSc and LS, downstream pathways leading to fibrosis may be more similar. We believe that the fibrosis seen in LS may represent the sequelae of the inflammatory damage; therefore, early treatment to abrogate inflammation during the inflammatory stage is imperative [38].

A key novel finding is the presence of genotoxic stress markers, including the upregulation of DNA damage response pathways and increased fusion transcript events, which have not been previously reported in LS. cGAS-STING pathway activation, driven by cytoplasmic DNA fragments, could be a potential mechanism for the observed IFN release [39]. This pathway was implicated in systemic autoimmune diseases like systemic lupus erythematosus (SLE) and SSc, where chronic IFN production can perpetuate immune activation [40]. The evidence from our study suggests that DNA instability and mitochondrial dysfunction, likely induced by environmental stressors such as those reported in association with LS, may drive chronic IFN signaling.

A novel and significant finding in this study reveals that immunosenescence plays a critical role in the pathogenesis of LS. We observed the upregulation of pathways associated with T cell exhaustion (e.g., PD-1, CTLA-4), cellular senescence, reduced mitochondrial function, and autophagy, especially in active and inflammatory LS. Immunosenescence, typically a natural part of aging, can be prematurely triggered by environmental or drug-induced factors, such as chemotherapy or chronic inflammation [41]. Chronic type 1 IFN release exacerbates immune exhaustion, accelerates telomere shortening, and contributes to DNA instability, leading to impaired immune responses and “inflammaging” [42,43]. This persistent inflammation fosters senescent cell expansion, promoting fibrosis through Type 2 immune skewing and architectural changes in endothelial cells and fibroblasts [44,45]. Moreover, clonal expansions of aging-associated B cells (ABCs) were implicated in other autoimmune diseases, such as SSc [46]. Our findings align with recent evidence of genomic instability and senescence signatures in early SSc, further linking immunosenescence to fibrotic diseases [41].

Our findings suggest a new theory for LS pathogenesis. In patients with inherited *STAT4* mutations, strong IFN signaling may drive premature immunosenescence. In idiopathic cases, a combination of genetic predisposition and external triggers (e.g., radiation, chemotherapy, trauma) could induce DNA damage, leading to immunosenescence. This mechanism may explain the diverse immune responses in LS and its known association with cancer [47]. Future studies are needed to validate this hypothesis.

This study reanalyzed publicly available RNA-seq data, limiting the ability to conduct confirmatory experiments. The relatively small sample size may reduce statistical power, especially for subgroup analyses. While multiple testing corrections were applied, this may have further decreased sensitivity to detect significant findings. The use of deconvolution algorithms like xCell and CIBERSORT comes with limitations related to their reliance on reference gene profiles derived from isolated cell populations, which may not fully capture in vivo cell heterogeneity. Additionally, while recent studies suggest RNA quality from FFPE samples is comparable to fresh-frozen, FFPE RNA remains more prone to degradation [10]. Finally, as our study focused on pediatric LS, the findings may not be generalizable to adult cases.

Our study highlights the complex immune landscape of LS, characterized by strong Type 1 IFN signaling, Type 2 skewing, DNA instability, and immunosenescence. Understanding these pathways opens new avenues for therapeutic interventions. The potential repurposing of existing drugs targeting these pathways, such as nintedanib (a tyrosine kinase inhibitor), tocilizumab (targeting the IL-6 receptor), and JAK/STAT inhibitors, could be explored for LS treatment [48,49,50].

Future research should focus on expanding the sample size, validating these findings in an independent cohort of LS patients including experimental validation, and exploring targeted therapies based on the molecular signatures identified in this study. Additionally, investigating the role of environmental exposures in driving genomic instability and IFN release may offer further insights into LS pathogenesis and prevention strategies.

## Figures and Tables

**Table 1:** Demographics and clinical characteristics. Adapted from Table 1, Schutt et al. [2] and supplemented by data uploaded from Geo and obtained from K.T. n, number; ssDNA, single stranded DNA; Th2, T helper.

Patient Characteristics	LS (n = 28)
Female sex (n, %)	17 (61)
Age at the time of biopsy, median (IQR)	13 (10–16)
Race White (n, %) Other (n, %)	25 (89) 3 (11)
Disease subtype Linear trunk/limbs (n, %) Linear head (n, %) Circumscribed (n, %) Generalized (n, %)	8 (29) 4 (14) 8 (29) 8 (29)
Disease activity Active (n, %) Inactive (n, %)	19 (68) 9 (32)
Histologic subtype Inflammatory	
Yes (n, %) No (n, %)	12 (43) 16 (57)
Severity grade None/mild (n, %) Moderate (n, %) Severe (n, %)	19 (68) 3 (11) 6 (21)
Collagen thickness in μm Papillary dermis (range) Upper reticular dermis (range) Lower reticular dermis (range)	1.92–7.44 6.17–41.09 12.10–74.25
Comorbid Th2 Diseases Eczema (n, %) Seasonal Allergies (n, %) Asthma (n, %)	5 (18) 3 (11) 1 (4)
ssDNA antibody positivity (n, %)	8 (29)

## Data Availability

Publicly available high-throughput RNA sequencing analyzed in this study was retrieved from the Gene Expression Omnibus (GEO) database “www.ncbi.nlm.nih.gov/geo/ (accessed on 30 August 2023)” with the accession number GSE166863.

## Supplementary Materials

The following supporting information can be downloaded at: https://www.mdpi.com/article/10.3390/ijms26031258/s1.

## References

[1] Laxer R.M., Zulian F. (2006). Localized scleroderma. Curr. Opin. Rheumatol..

[2] Schutt C., Mirizio E., Salgado C., Reyes-Mugica M., Wang X., Chen W., Grunwaldt L., Schollaert K.L., Torok K.S. (2021). Transcriptomic Evaluation of Juvenile Localized Scleroderma Skin With Histologic and Clinical Correlation. Arthritis Rheumatol..

[3] CPapara, De Luca D.A., Bieber K., Vorobyev A., Ludwig R.J. (2023). Morphea: The 2023 update. Front. Med..

[4] Tsou P.-S., Shi B., Varga J. (2022). Role of cellular senescence in the pathogenesis of systemic sclerosis. Curr. Opin. Rheumatol..

[5] Gao Y., Cai W., Zhou Y., Li Y., Cheng J., Wei F. (2022). Immunosenescence of T cells: A key player in rheumatoid arthritis. Inflamm. Res..

[6] Zulian F., Culpo R., Sperotto F., Anton J., Avcin T., Baildam E.M., Boros C., Chaitow J., Constantin T., Kasapcopur O. (2019). Consensus-based recommendations for the management of juvenile localised scleroderma. Ann. Rheum. Dis..

[7] Wang L., Wang S., Li W. (2012). RSeQC: Quality control of RNA-seq experiments. Bioinformatics.

[8] Graubert A., Aguet F., Ravi A., Ardlie K.G., Getz G. (2021). RNA-SeQC 2: Efficient RNA-seq quality control and quantification for large cohorts. Bioinformatics.

[9] Leon K.E., Buj R., Lesko E., Dahl E.S., Chen C.-W., Tangudu N.K., Imamura-Kawasawa Y., Kossenkov A.V., Hobbs R.P., Aird K.M. (2021). DOT1L modulates the senescence-associated secretory phenotype through epigenetic regulation of IL1A. J. Cell Biol..

[10] Grzanka M., Piekiełko-Witkowska A. (2021). The Role of Gene in Health and Disease: Beyond Treacher Collins Syndrome. Int. J. Mol. Sci..

[11] Edgar R., Domrachev M., Lash A.E. (2002). Gene Expression Omnibus: NCBI gene expression and hybridization array data repository. Nucleic Acids Res..

[12] Mirizio E., Liu C., Yan Q., Waltermire J., Mandel R., Schollaert K.L., Konnikova L., Wang X., Chen W., Torok K.S. (2021). Genetic Signatures From RNA Sequencing of Pediatric Localized Scleroderma Skin. Front. Pediatr..

[13] Newman A.M., Liu C.L., Green M.R., Gentles A.J., Feng W., Xu Y., Hoang C.D., Diehn M., Alizadeh A.A. (2015). Robust enumeration of cell subsets from tissue expression profiles. Nat. Methods.

[14] Robinson M.D., McCarthy D.J., Smyth G.K. (2010). edgeR: A Bioconductor package for differential expression analysis of digital gene expression data. Bioinformatics.

[15] Asano Y., Fujimoto M., Ishikawa O., Sato S., Jinnin M., Takehara K., Hasegawa M., Yamamoto T., Ihn H. (2018). Diagnostic criteria, severity classification and guidelines of localized scleroderma. J. Dermatol..

[16] Benjamini Y. (2010). Discovering the false discovery rate: False Discovery Rate. J. R. Stat. Soc. Series B Stat. Methodol..

[17] Sherman B.T., Hao M., Qiu J., Jiao X., Baseler M.W., Lane H.C., Imamichi T., Chang W. (2022). DAVID: A web server for functional enrichment analysis and functional annotation of gene lists (2021 update). Nucleic Acids Res..

[18] Huang D.W., Sherman B.T., Lempicki R.A. (2009). Systematic and integrative analysis of large gene lists using DAVID bioinformatics resources. Nat. Protoc..

[19] Kolde (2019). Pheatmap: Pretty Heatmaps.

[20] Kanehisa M., Goto S. (2000). KEGG: Kyoto encyclopedia of genes and genomes. Nucleic Acids Res..

[21] Chen J., Bardes E.E., Aronow B.J., Jegga A.G. (2009). ToppGene Suite for gene list enrichment analysis and candidate gene prioritization. Nucleic Acids Res..

[22] Tacutu R., Thornton D., Johnson E., Budovsky A., Barardo D., Craig T., Diana E., Lehmann G., Toren D., Wang J. (2018). Human Ageing Genomic Resources: New and updated databases. Nucleic Acids Res..

[23] Mootha V.K., Lindgren C.M., Eriksson K.-F., Subramanian A., Sihag S., Lehar J., Puigserver P., Carlsson E., Ridderstråle M., Laurila E. (2003). PGC-1alpha-responsive genes involved in oxidative phosphorylation are coordinately downregulated in human diabetes. Nat. Genet..

[24] Aran D., Hu Z., Butte A.J. (2017). xCell: Digitally portraying the tissue cellular heterogeneity landscape. Genome Biol..

[25] Wickham (2009). Ggplot2: Elegant Graphics for Data Analysis.

[26] Haas B.J., Dobin A., Stransky N., Li B., Yang X., Tickle T., Bankapur A., Ganote C., Doak T.G., Pochet N. (2017). STAR-Fusion: Fast and Accurate Fusion Transcript Detection from RNA-Seq. bioRxiv.

[27] Uhrig S., Ellermann J., Walther T., Burkhardt P., Fröhlich M., Hutter B., Toprak U.H., Neumann O., Stenzinger A., Scholl C. (2021). Accurate and efficient detection of gene fusions from RNA sequencing data. Genome Res..

[28] PEwels, Magnusson M., Lundin S., Käller M. (2016). MultiQC: Summarize analysis results for multiple tools and samples in a single report. Bioinformatics.

[29] Baghdassarian H., Blackstone S.A., Clay O.S., Philips R., Matthiasardottir B., Nehrebecky M., Hua V.K., McVicar R., Liu Y., Tucker S.M. (2023). Variant and Response to Ruxolitinib in an Autoinflammatory Syndrome. N. Engl. J. Med..

[30] Jacobe H., Ahn C., Arnett F.C., Reveille J.D. (2014). Major histocompatibility complex class I and class II alleles may confer susceptibility to or protection against morphea: Findings from the Morphea in Adults and Children cohort. Arthritis Rheumatol..

[31] Saracino A.M., Denton C.P., Orteu C.H. (2017). The molecular pathogenesis of morphoea: From genetics to future treatment targets. Br. J. Dermatol..

[32] Laetsch B., Hofer T., Lombriser N., Lautenschlager S. (2011). Irradiation-Induced Morphea: X-Rays as Triggers of Autoimmunity. Dermatology.

[33] Beigi P.K.M. (2022). The Immunogenetics of Morphea and Lichen Sclerosus. Adv. Exp. Med. Biol..

[34] Partl R., Regitnig P., Lukasiak K., Winkler P., Kapp K.S. (2020). Incidence of Morphea following Adjuvant Irradiation of the Breast in 2,268 Patients. Breast Care.

[35] Hanami Y., Ohtsuka M., Yamamoto T. (2016). Paraneoplastic eosinophilic fasciitis with generalized morphea and vitiligo in a patient working with organic solvents. J. Dermatol..

[36] Aounallah A., Lahouel I., Mokni S., Ksiaa M., Hayouni M., Belajouza C., Kotti F., Saidi W., Boussofara L., El Maalel O. (2018). Atypical generalized morphea-like scleroderma occurring in a patient exposed to organic solvents and having chronic hepatitis C virus infection. Indian J. Dermatol. Venereol. Leprol..

[37] Truchetet M.E., Brembilla N.C., Chizzolini C. (2023). Current concepts on the pathogenesis of systemic sclerosis. Clin. Rev. Allergy Immunol..

[38] O’Brien J.C., Nymeyer H., Green A., Jacobe H.T. (2020). Changes in Disease Activity and Damage Over Time in Patients With Morphea. JAMA Dermatol..

[39] Huang Y., Liu B., Sinha S.C., Amin S., Gan L. (2023). Mechanism and therapeutic potential of targeting cGAS-STING signaling in neurological disorders. Mol. Neurodegener..

[40] Lood C., Blanco L.P., Purmalek M.M., Carmona-Rivera C., De Ravin S.S., Smith C.K., Malech H.L., A Ledbetter J., Elkon K.B., Kaplan M.J. (2016). Neutrophil extracellular traps enriched in oxidized mitochondrial DNA are interferogenic and contribute to lupus-like disease. Nat. Med..

[41] Gniadecki R., Iyer A., Hennessey D., Khan L., O’Keefe S., Redmond D., Storek J., Durand C., Cohen-Tervaert J.W., Osman M. (2022). Genomic instability in early systemic sclerosis. J. Autoimmun..

[42] ALonde C., Fernandez-Ruiz R., Julio P.R., Appenzeller S., Niewold T.B. (2023). Type I Interferons in Autoimmunity: Implications in Clinical Phenotypes and Treatment Response. J. Rheumatol..

[43] Zhu C., Gabrielli S., Khoury L., Osman M., Litvinov I.V., Iannattone L., Lefrançois P., Netchiporouk E. (2023). Immunosenescence, Inflammaging, and Dermatology: Insights and Opportunities. J. Cutan. Med. Surg..

[44] Mogilenko D.A., Shchukina I., Artyomov M.N. (2021). Immune ageing at single-cell resolution. Nat. Rev. Immunol..

[45] Wang Y., Dong C., Han Y., Gu Z., Sun C. (2022). Immunosenescence, aging and successful aging. Front. Immunol..

[46] Ruan P., Wang S., Yang M., Wu H. (2022). The ABC-associated Immunosenescence and Lifestyle Interventions in Autoimmune Disease. Rheumatol. Immunol. Res..

[47] Joly-Chevrier M., Gélinas A., Ghazal S., Moussa S., McCuaig C.C., Piram M., Mereniuk A., Litvinov I.V., Osman M., Pehr K. (2023). Morphea, Eosinophilic Fasciitis and Cancer: A Scoping Review. Cancers.

[48] Wijsenbeek M., Swigris J.J., Inoue Y., Kreuter M., Maher T.M., Suda T., Baldwin M., Mueller H., Rohr K.B., Flaherty K.R. (2024). Effects of nintedanib on symptoms in patients with progressive pulmonary fibrosis. Eur. Respir. J..

[49] Lonowski S., Goldman N., Kassamali B., Shahriari N., LaChance A., Vleugels R.A. (2022). Tocilizumab for refractory morphea in adults: A case series. JAAD Case Rep..

[50] Damsky W., Patel D., Garelli C.J., Garg M., Wang A., Dresser K., Deng A., Harris J.E., Richmond J., King B. (2020). Jak Inhibition Prevents Bleomycin-Induced Fibrosis in Mice and Is Effective in Patients with Morphea. J. Invest. Dermatol..

