# Supplementary material for: Markers of Type 2 Inflammation and Immunosenescence Are Upregulated in Localized Scleroderma
Source: Int J Mol Sci. 2025 Jan 31;26(3):1258. doi: 10.3390/ijms26031258 (PMC11818363; doi:10.3390/ijms26031258)
Supplement: Supplementary file 1 [file ijms-26-01258-s001.zip › ijms-3384224-supplementary.pdf]

Supporting information for

Markers of Type 2 Inflammation and Immunosenescence are Upregulated in Localized Scleroderma

Lauren Khoury [1], Connor Prosty [1], Stephanie Ghazal [2], Sofianne Gabrielli [1], Kathryn S Torok [3], Mohammed Osman [4], Elvis Martinez-Jaramillo [2], Philippe Lefrançois [5], Elena Netchiporouk [2]

Correspondence to: elena.netchiporouk@mcgill.ca

[1] Faculty of Medicine, McGill University, Montreal, QC, Canada.
[2] Division Dermatology, McGill University Health Centre, Montreal, QC, Canada.
[3] University of Pittsburgh, University of Pittsburgh Medical Center, Children's Hospital of Pittsburgh, Pittsburgh, Pennsylvania.
[4] Division of Rheumatology, University of Alberta, Edmonton, Alberta, Canada.
[5] Division Dermatology, Jewish General Hospital, Montreal, QC, Canada.

This file includes:

Figures S1 to S18.
Tables S1 to S11.

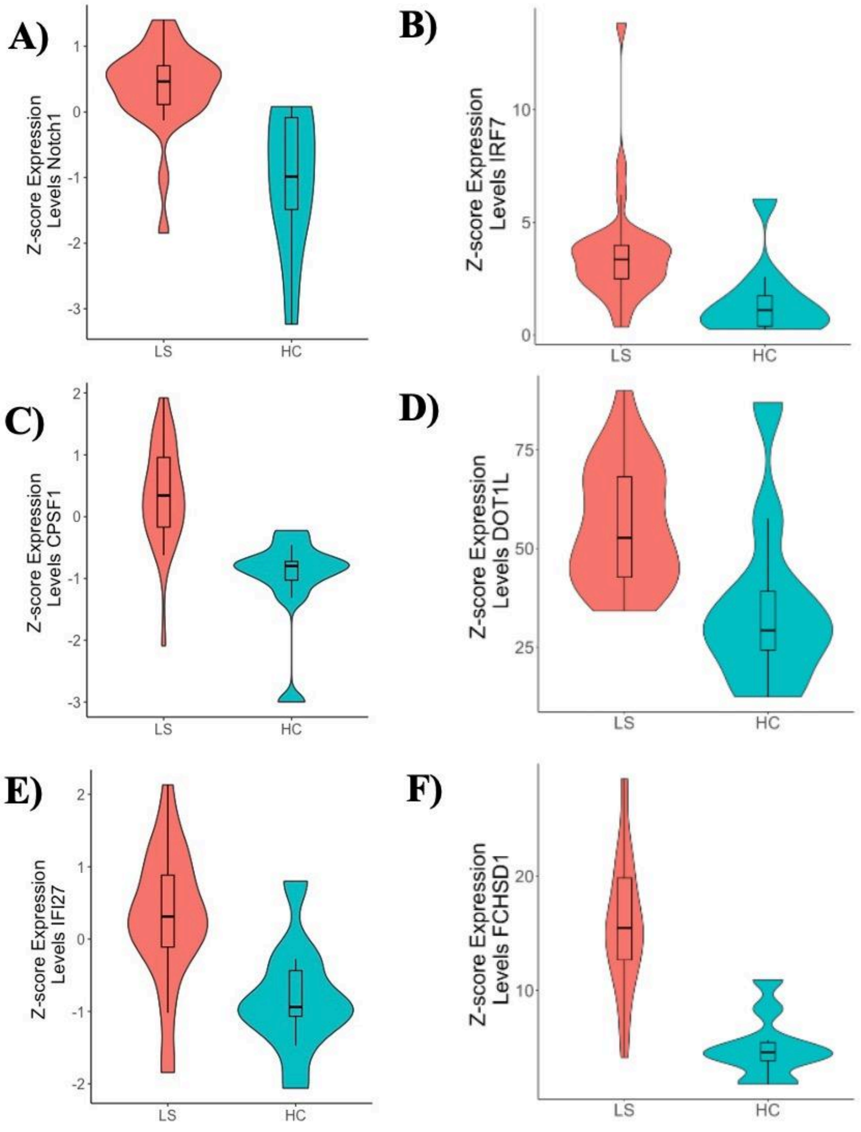

Figure S1. Violin Plots of Selected Genes of Interest in Pediatric LS *vs.* HC. Violin plots of differentially expressed genes in LS *vs.* HC samples. (A) *Notch1* involved in the regulation of T cell proliferation, differentiation, and apoptosis (*Q*=5.72E-04, logFc =1.14), (B) *IRF7* involved in regulating interferon-beta, gamma, alpha signaling and responds to DNA damage (*Q*=1.77E-02, logFc =1.63), (C) *CPSF1* involved in mRNA processing (*Q*=4.78E-03, logFc =1.38), (D) *DOT1L* involved in DNA damage repair (*Q*=3.03E-03, logFc =1.12), (E) *IFI27* involved in type 1 interferon signaling (*Q*=4.69E-02, logFc =1.70), (F) *FCHSD1* involved in the regulation of actin filament polymerization (*Q*=2.80E-03, logFc =1.41). Function is adapted from Database for Annotation, Visualization and Integrated Discovery (DAVID). LS, localized scleroderma; HC, healthy controls; adj.P.value, adjusted P value.

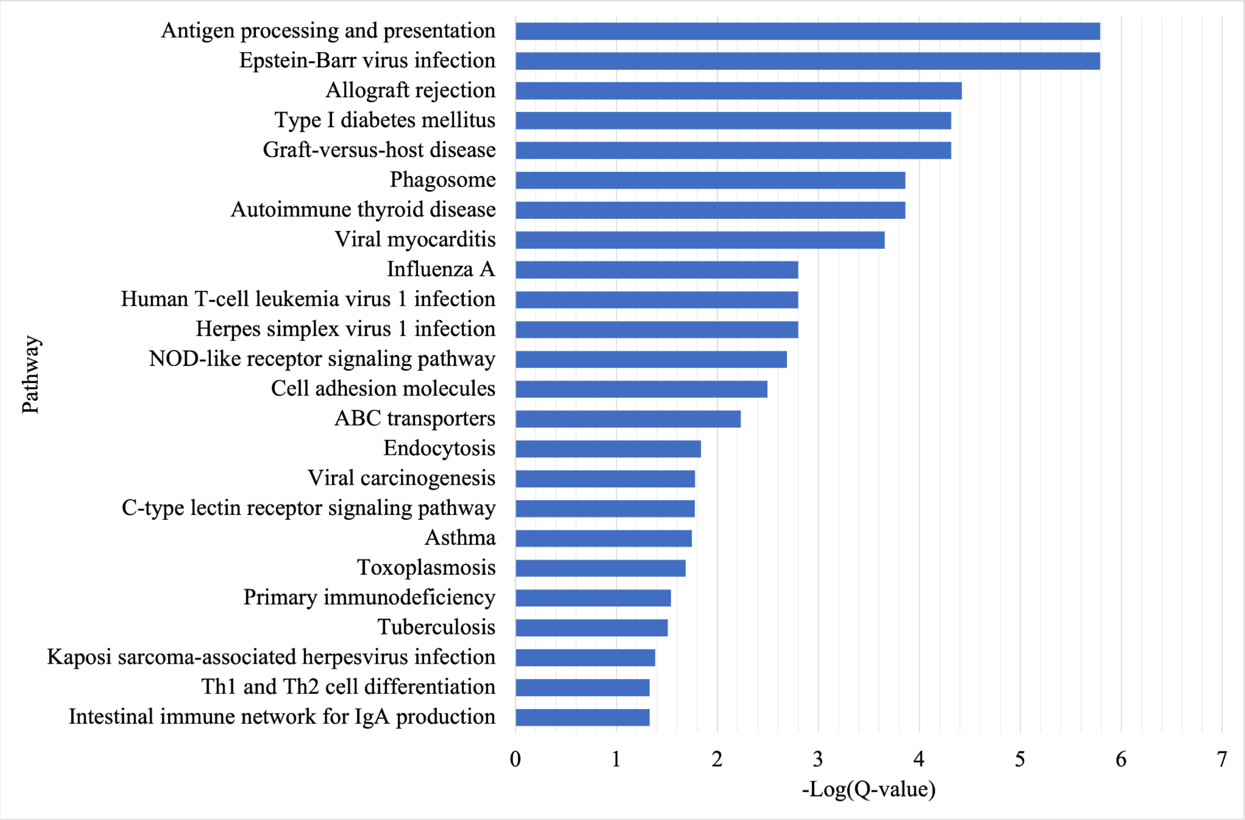

Figure S2. Top 20 upregulated KEGG pathways in LS *vs.* HC. The remaining pathways are type 1 diabetes mellitus, allograft rejection, and Epstein-Barr virus infection.

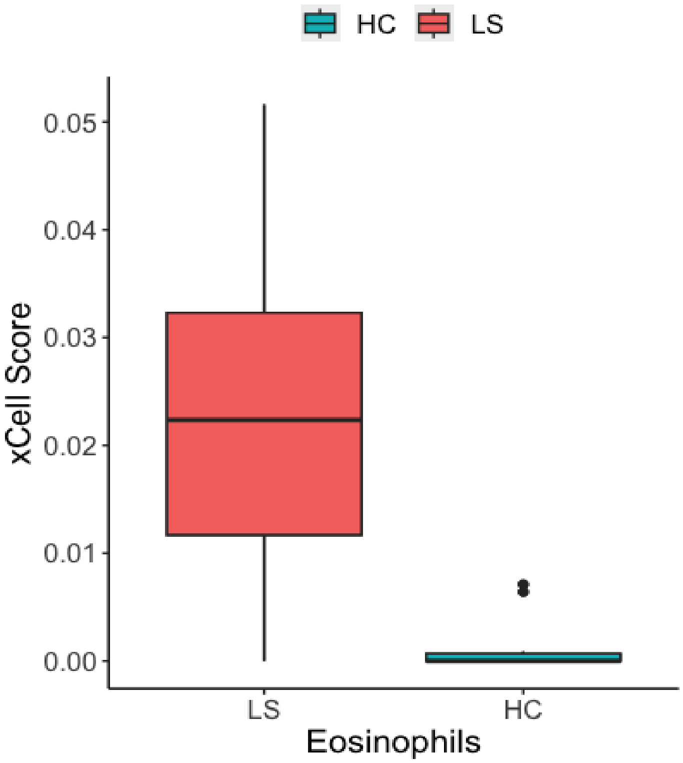

Figure S3. xCell scores for eosinophils in LS *vs*. HC. Eosinophils are overexpressed in LS lesional skin samples (*Q*=1.28E-02). LS, localized scleroderma; HC, healthy controls.

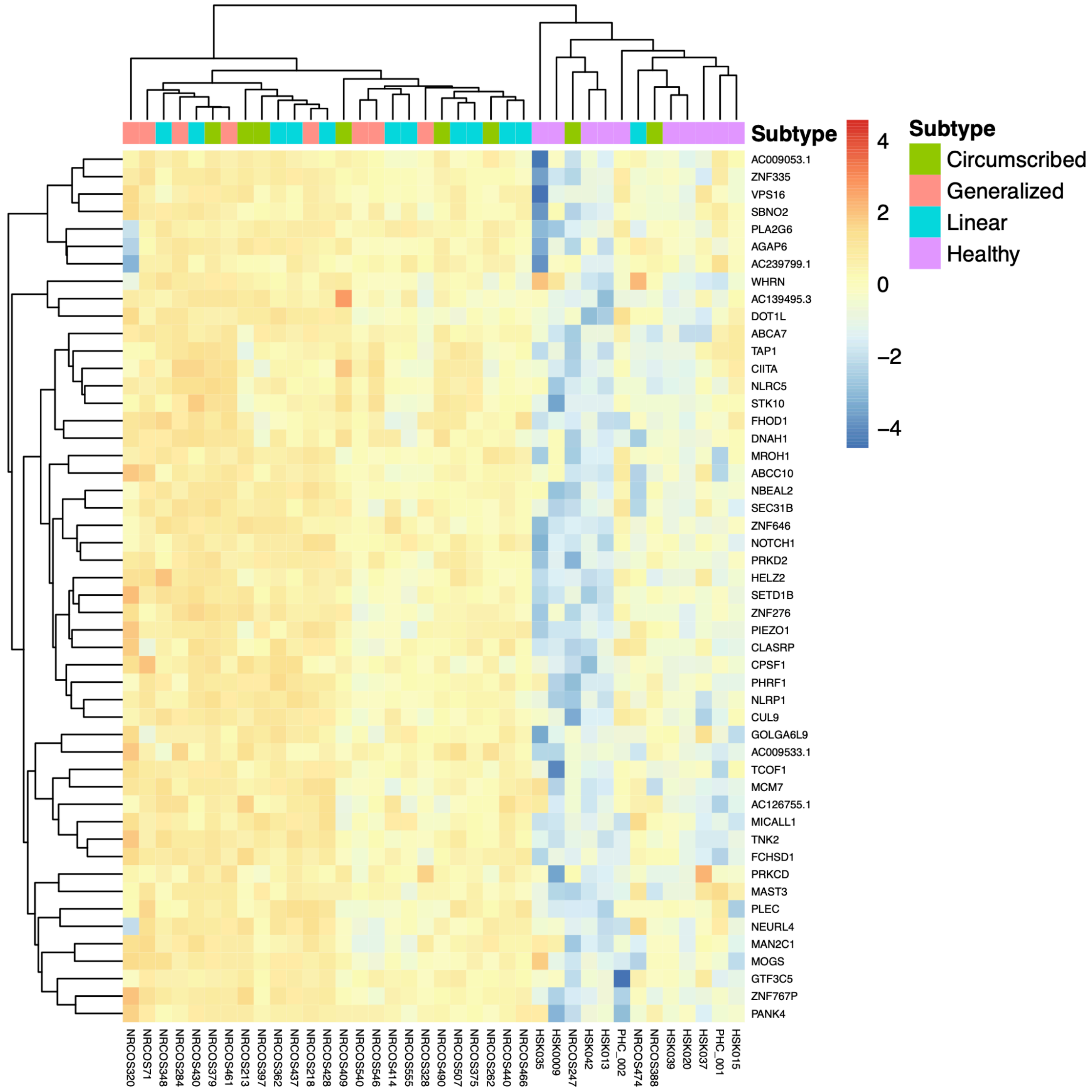

Figure S4. Unsupervised hierarchical clustering based on top 50 genes in linear (blue), circumscribed (green), generalized (red) LS *vs*. HC (purple). The color key indicates gene expression as a z-score. Circumscribed LS samples (n = 8) are indicated by green color, Generalized LS samples (n = 8) are indicated by blue color, Linear LS (n = 12) are indicated by blue color, and HC (n = 10) by purple. Three clusters are identified, one consisting of 3 Circumscribed LS, 5 Generalized LS, and 5 Linear. The second consists of 3 Circumscribed LS, 3 Generalized LS, and 6 Linear LS and the third consists of 3 Circumscribed LS, 1 Linear LS, and 10 HC.

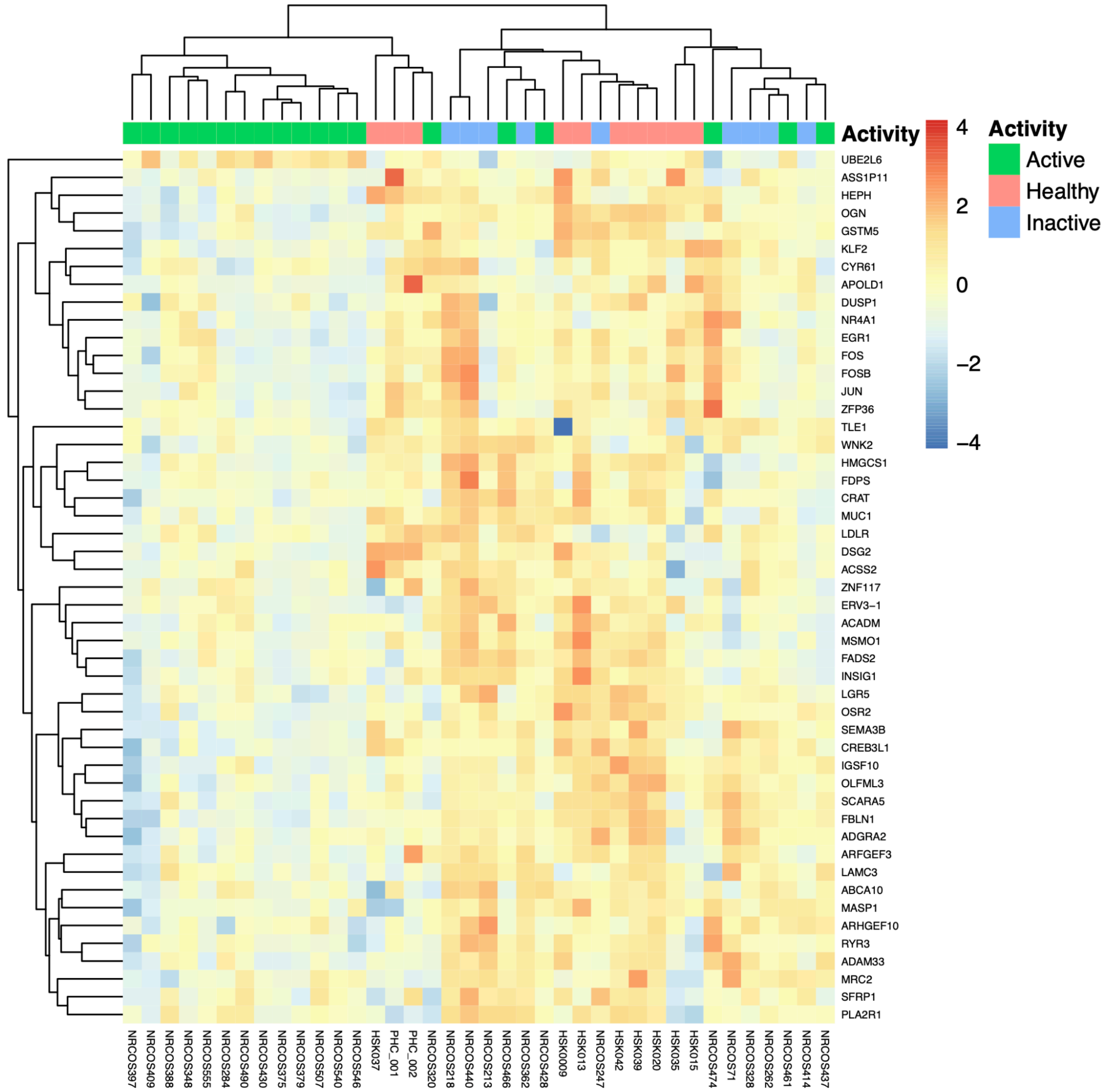

Figure S5. Unsupervised Hierarchical Clustering of Top 50 Upregulated Genes per Activity Status *vs.* HC. Active LS samples (n = 19) are indicated by green color, Inactive LS samples (n = 9) are indicated by blue color, and HC (n = 10) by red. Two clusters are identified, one consisting of 13 Active LS, 3 HC, and 1 Inactive LS and the second consists of 5 Active LS, 9 Inactive LS, and 7 HC. LS, localized scleroderma; HC, healthy controls.

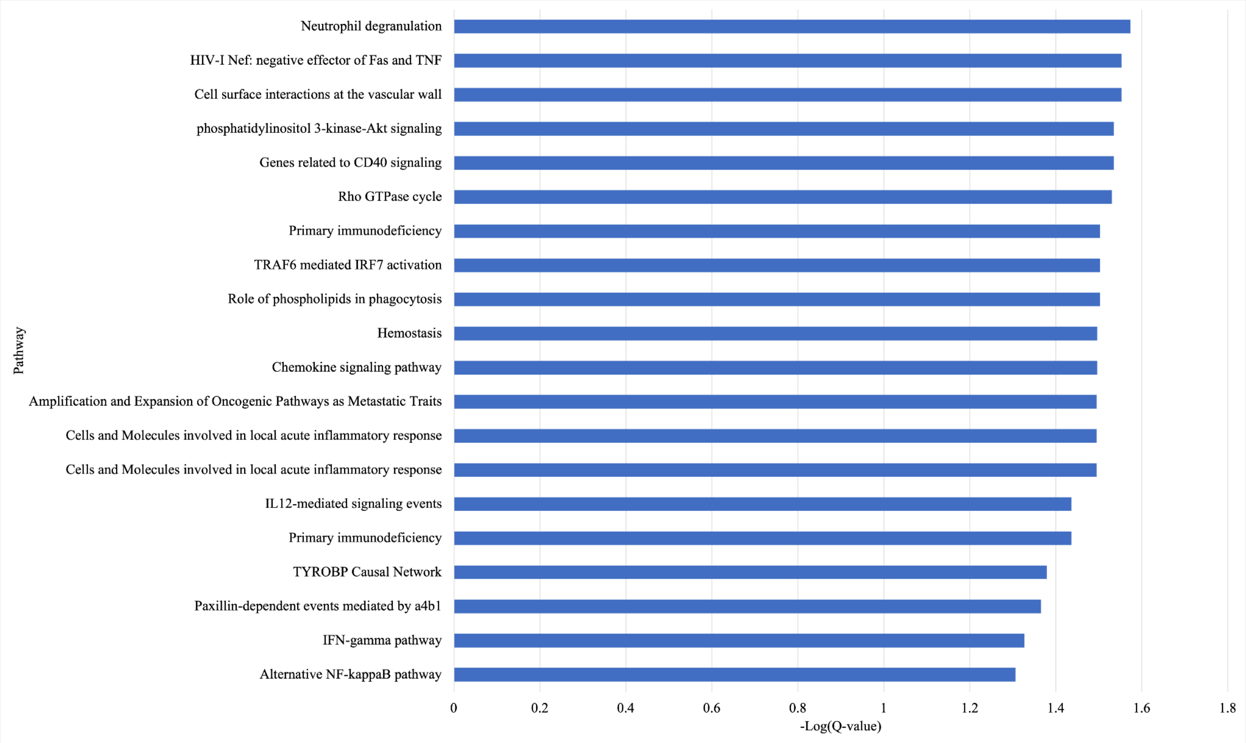

Figure S6. Top 20 upregulated ToppGene pathways in Active LS *vs.* HC. The remaining pathways include neutrophil degranulation, IL-4 signaling, MAPKinase signaling, interleukin signaling, SARS coronavirus and innate immunity, pathways in cancer, adhesion and diapedesis of lymphocytes, CTLA4 cancer immunotherapy, and measles.

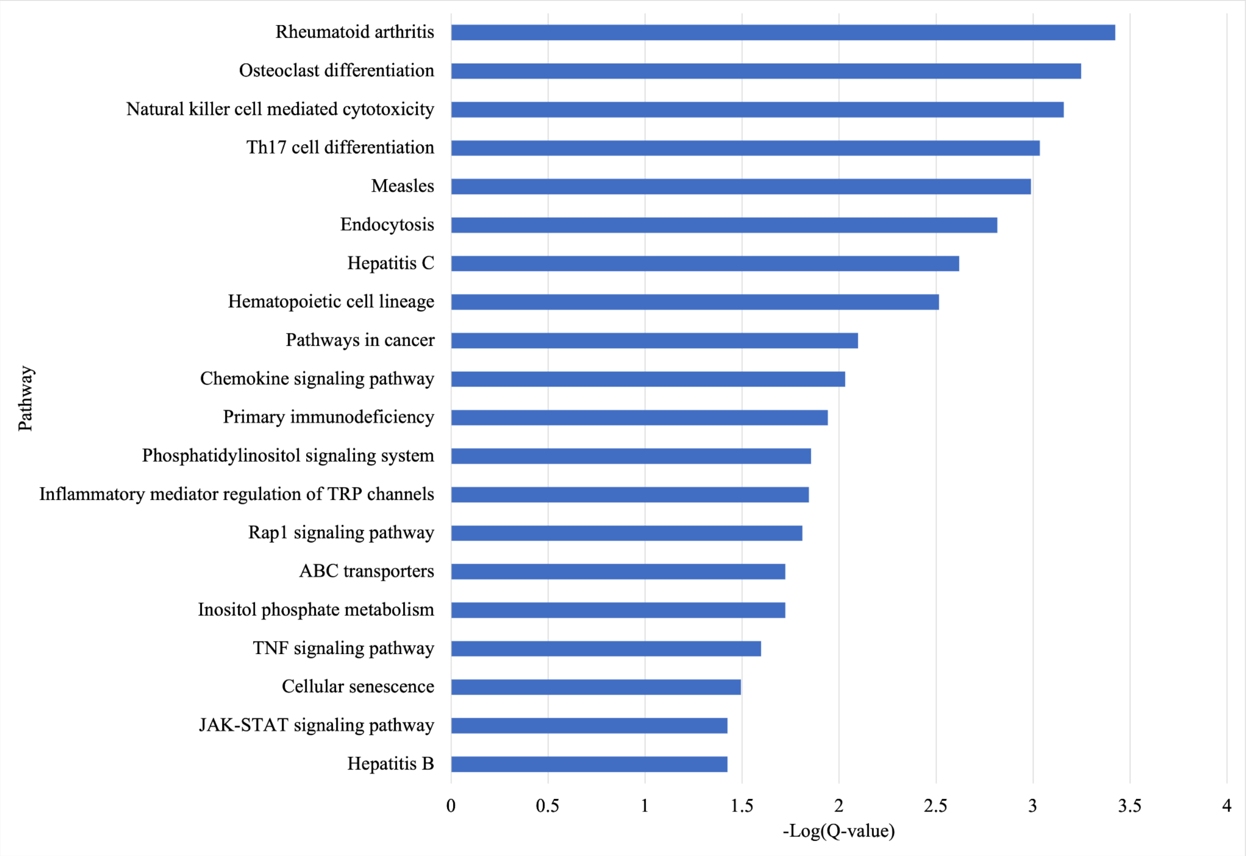

Figure S7. Top 20 upregulated KEGG pathways in Active LS *vs.* HC. The remaining pathways include human immunodeficiency virus 1 infection, inflammatory bowel disease, staphylococcus aureus infection, Th1 and Th2 cell differentiation, viral carcinogenesis, human papillomavirus infection, asthma, human cytomegalovirus infection, toxoplasmosis, and intestinal immune network for IgA production.

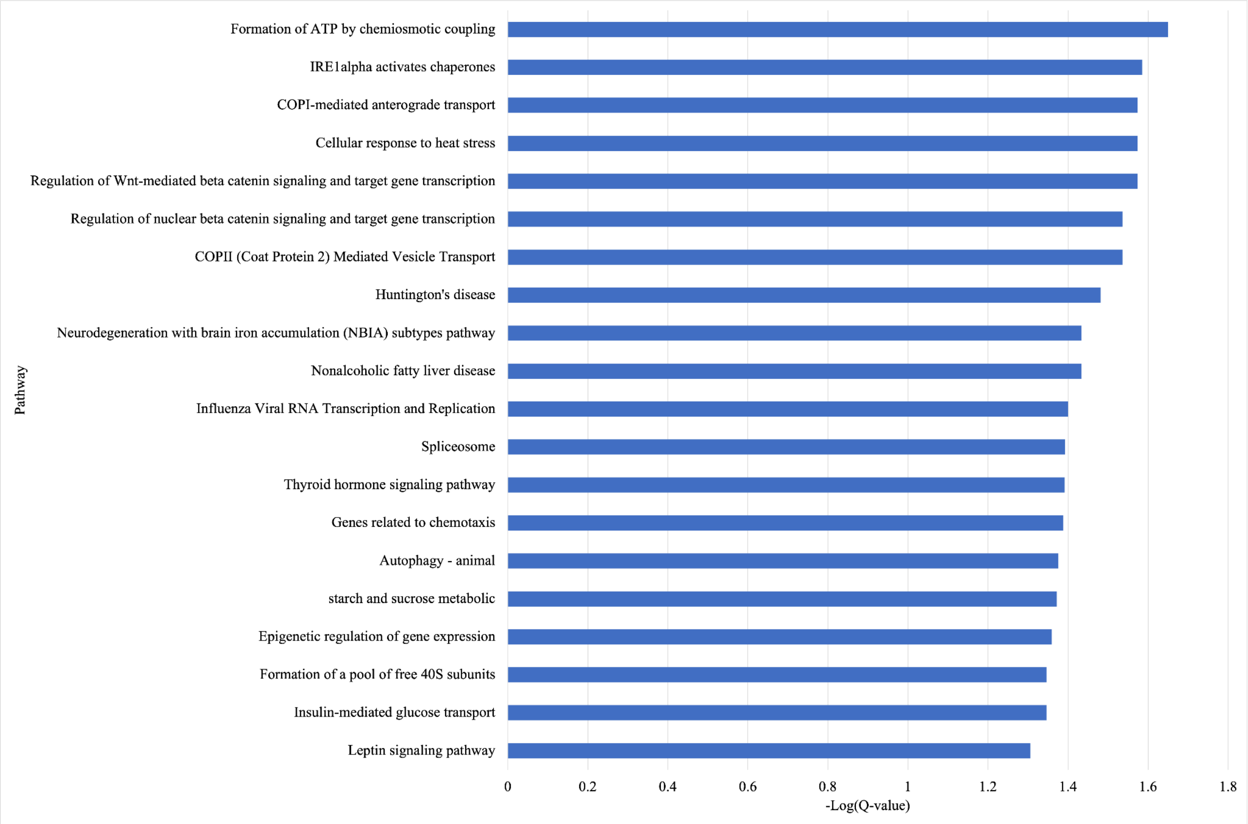

Figure S8. Top 20 downregulated ToppGene pathways in Active LS *vs*. HC. The remaining pathways include XBP1(S) activates chaperone genes, acute myeloid leukemia, RHO GTPase effectors, non-alcoholic fatty liver disease, SRP-dependent cotranslational protein targeting to membrane, RHO GTPases activate ROCKs, LKB1 signaling events, I3K-AKT-mTOR signaling pathway, and intra-golgi traffic.

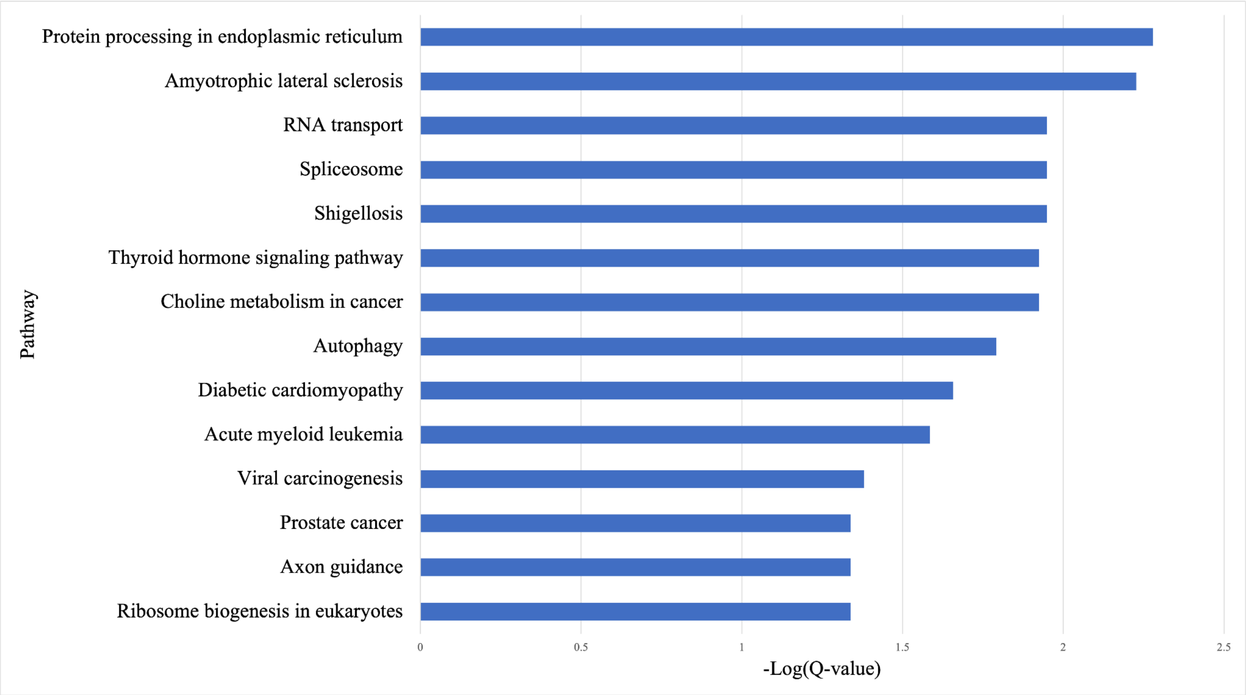

Figure S9. Top downregulated KEGG pathways in Active LS *vs.* HC.

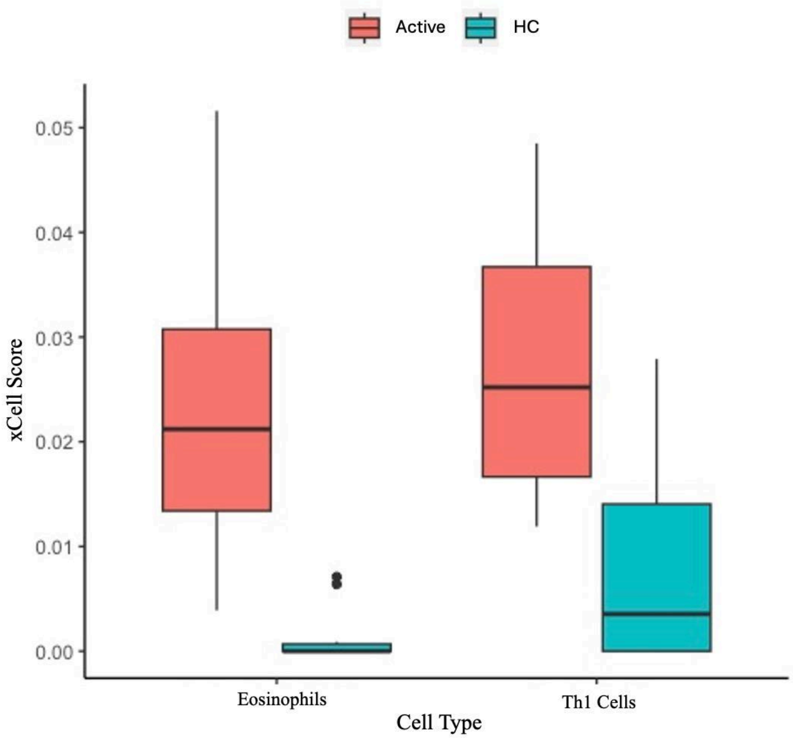

Figure S10. xCell scores for Th1 (*Q*=2.08E-02) and eosinophils (*Q*=2.05E-02) in Active LS *vs*. HC skin samples.

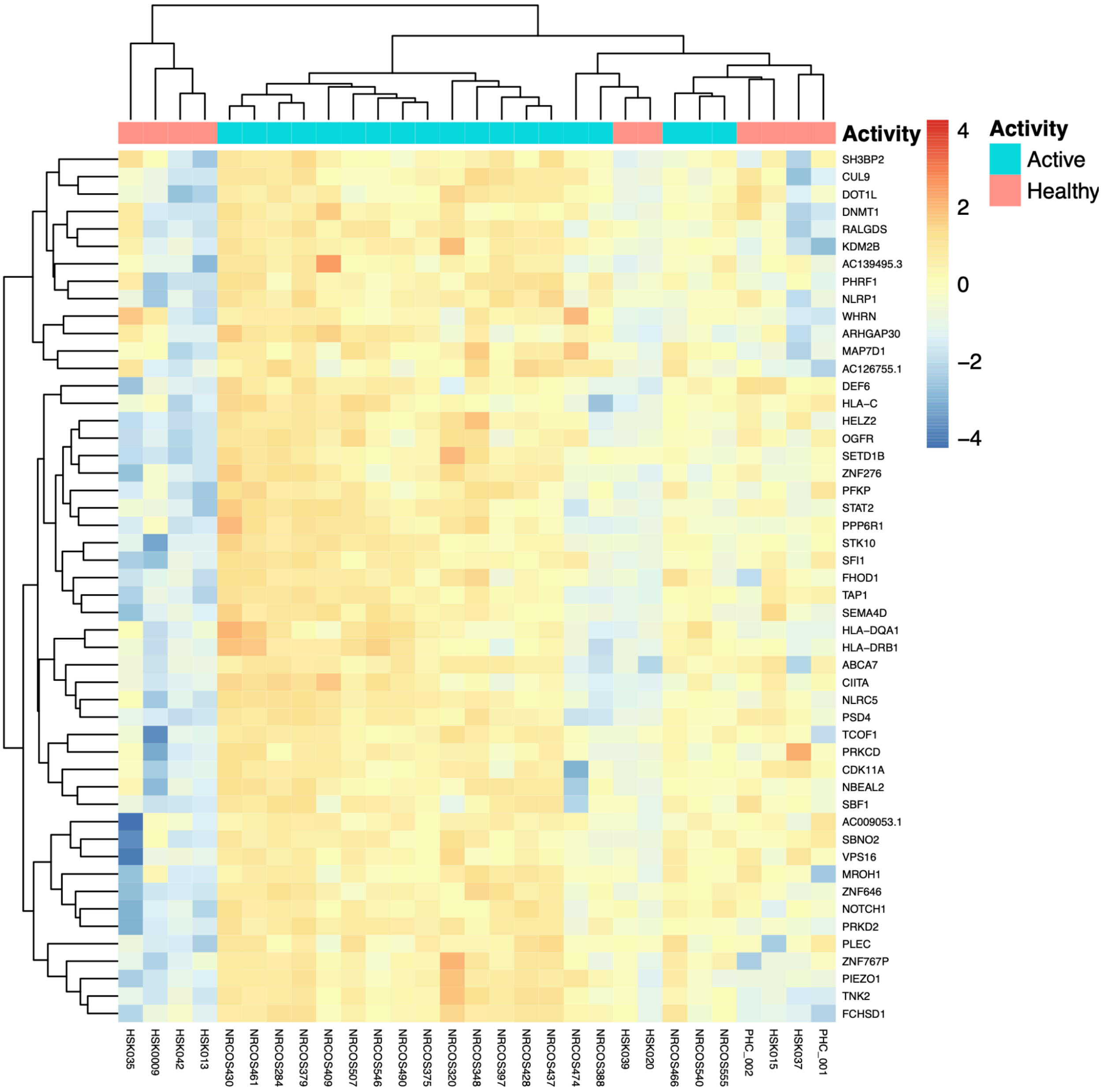

Figure S11. Unsupervised Hierarchical Clustering of Top 50 Upregulated Genes in Active LS *vs*. HC. Active LS samples (n = 19) are indicated by blue color, HC (n = 10) by red. Three clusters are identified, 1 consisting of 4 HCs, second of 14 LS and third of 5 LS and 6 HC. LS, localized scleroderma; HC, healthy controls.

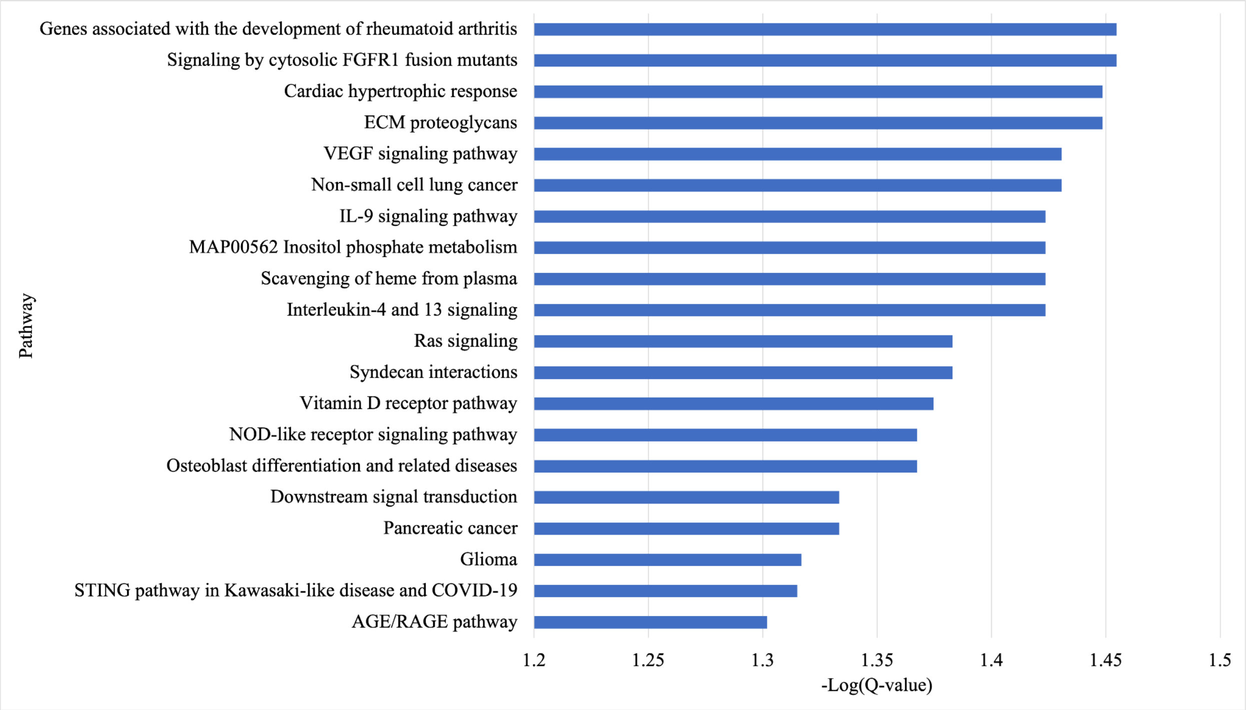

Figure S12. Top 20 upregulated ToppGene pathways in Inflammatory LS *vs.* HC. The remaining pathways include TGF-beta signaling in thyroid cells for epithelial-mesenchymal transition, complement system in neuronal development and plasticity, interferon type I signaling, IL-4 signaling, amplification and expansion of oncogenic pathways as metastatic traits, platelet-mediated interactions with vascular and circulating cells, inositol phosphate metabolism, toll-like receptor signaling, integrin signalling, and signaling events mediated by stem cell factor receptor.

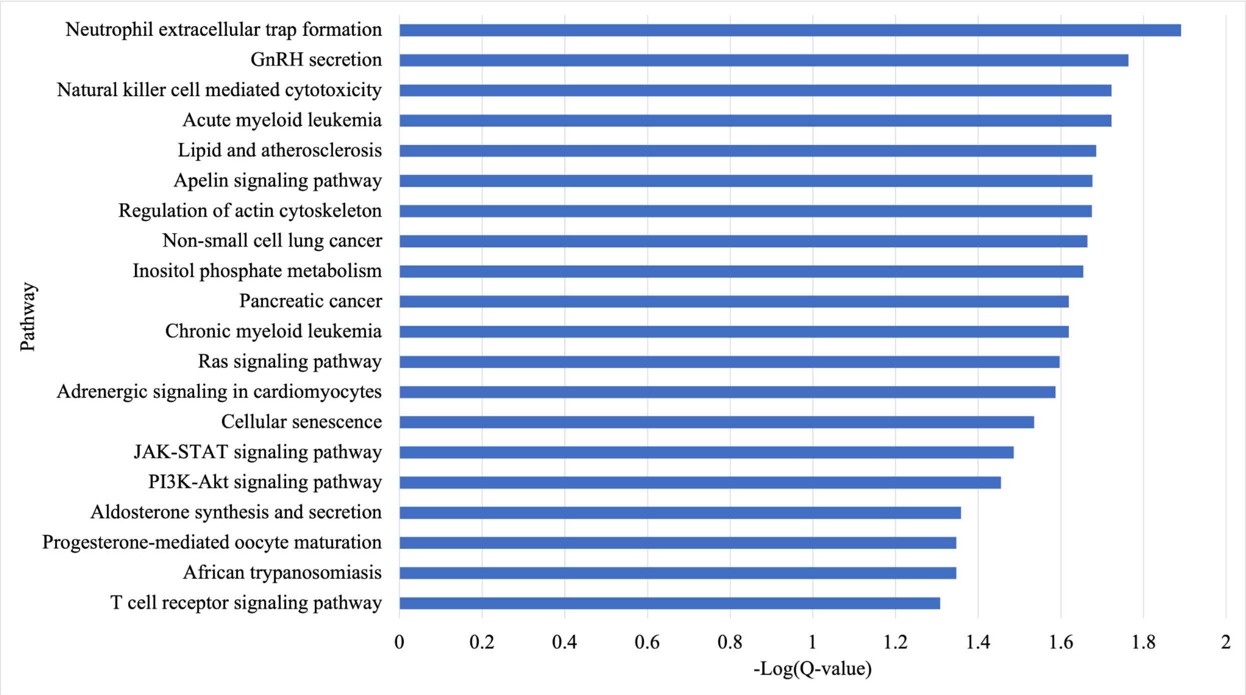

Figure S13. Top 20 upregulated KEGG pathways in Inflammatory LS *vs.* HC ranked. The remaining pathways include malaria, toll-like receptor signaling, salmonella, cGMP-PKG signaling, inflammatory mediator regulation of TRP channels, oxytocin signaling, PD-L1 expression and PD-1 checkpoint pathway in cancer, phospholipase D signaling, primary immunodeficiency, and human immunodeficiency virus 1 infection.

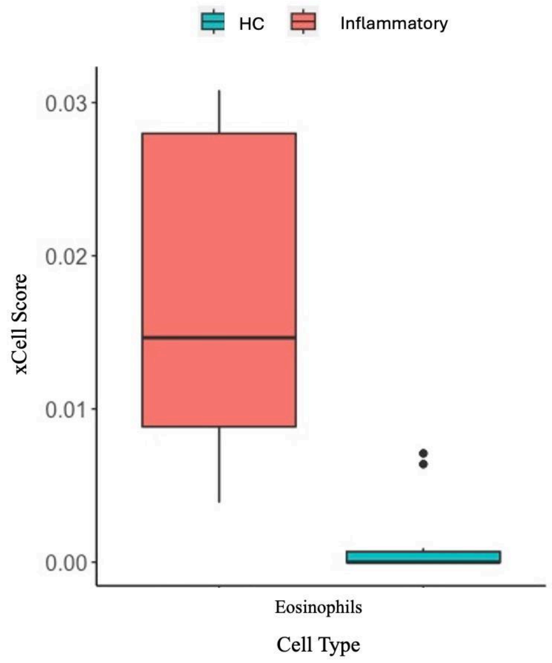

Figure S14. xCell scores for eosinophils (*Q*=3.52E-02) in inflammatory LS compared to HC skin samples.

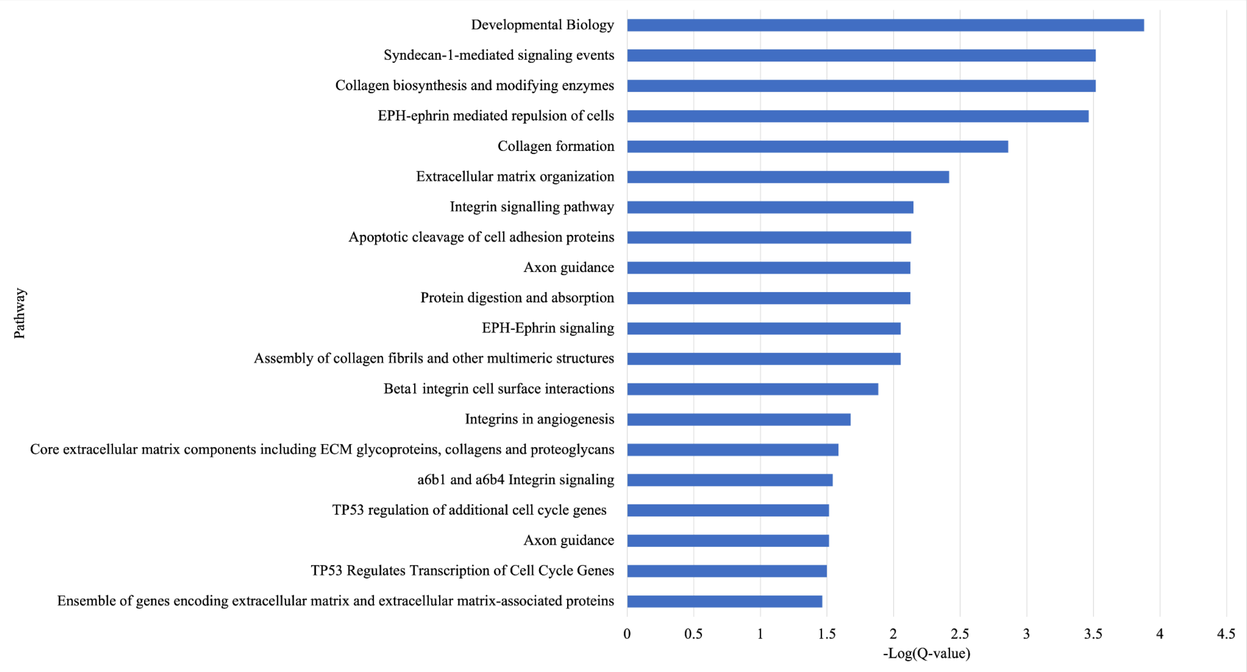

Figure S15. Top 20 upregulated ToppGene pathways in Fibrotic LS *vs*. HC. The remaining pathways are collagen chain trimerization, genes encoding collagen proteins, formation of the cornified envelope, and keratinization.

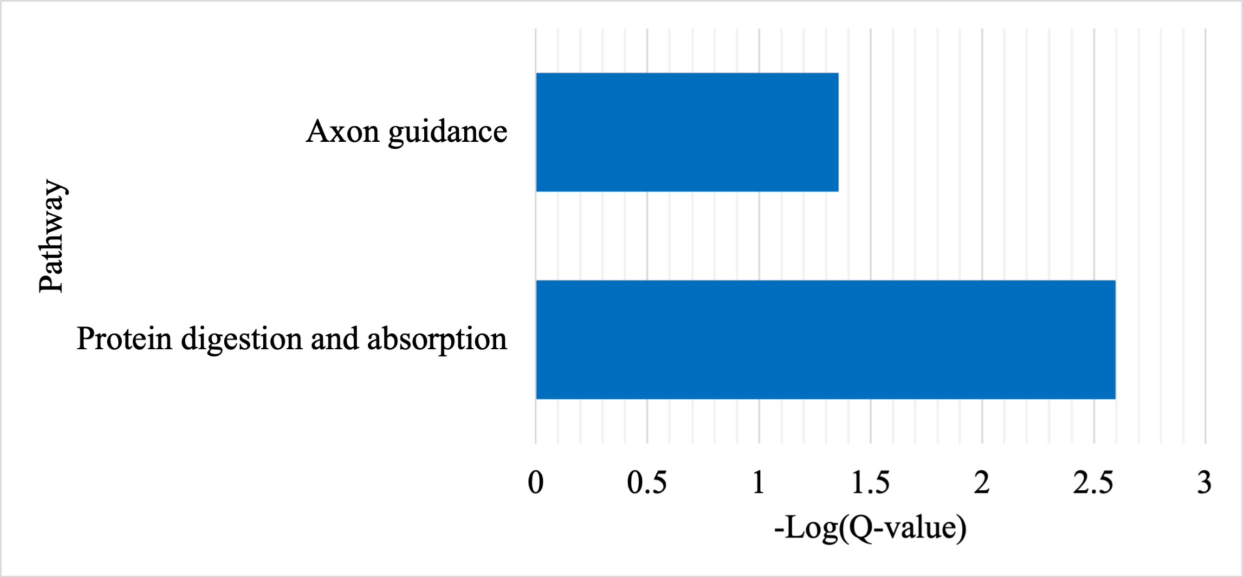

Figure S16. Top upregulated KEGG pathways in Fibrotic LS *vs.* HC.

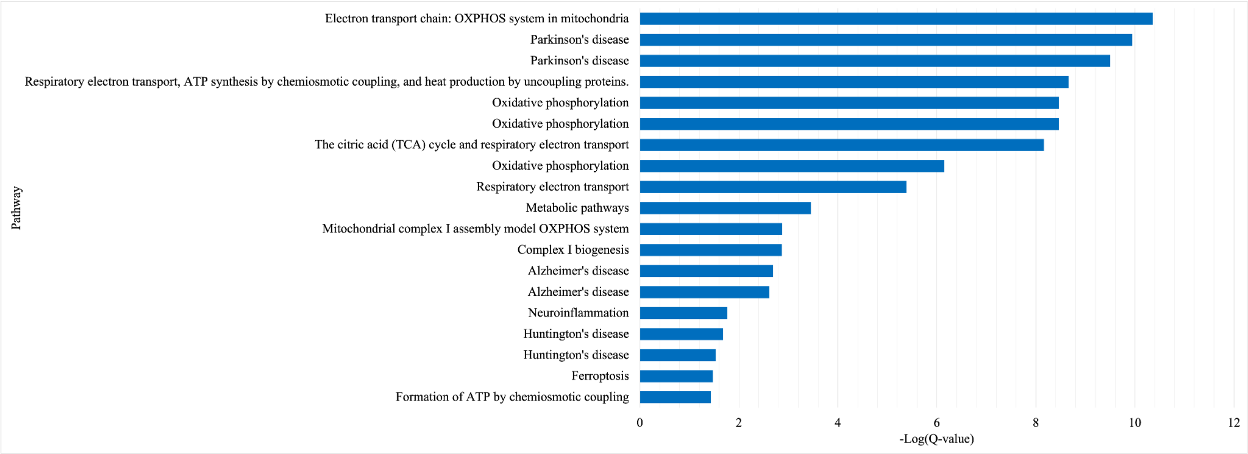

Figure S17. Downregulated ToppGene pathways in Fibrotic LS *versus* HC.

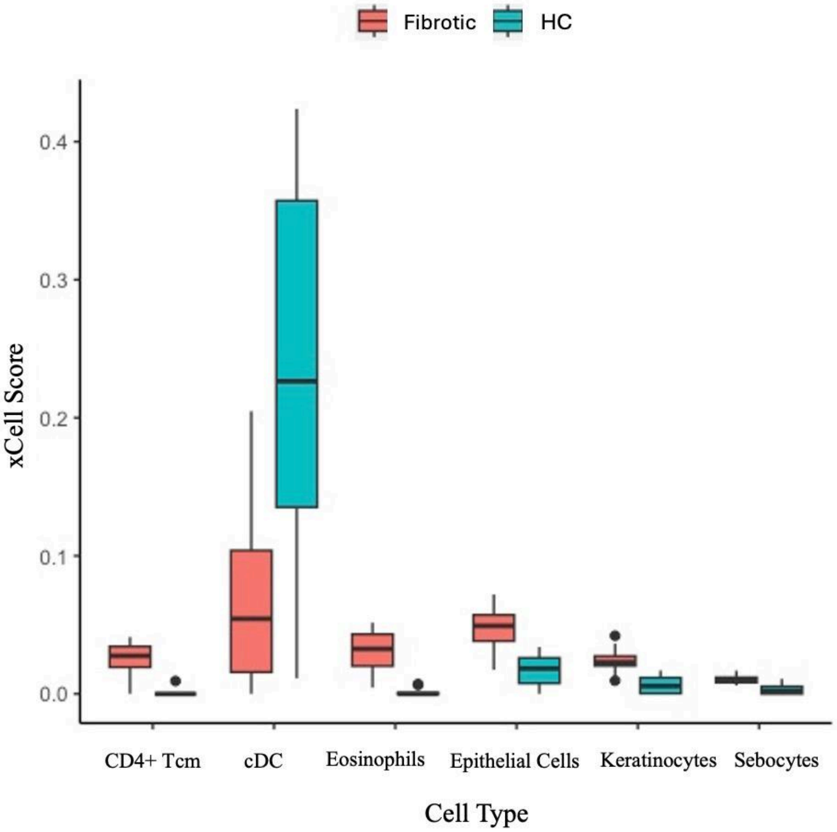

Figure S18. xCell scores for CD4+ Tcm, central dendritic cells, eosinophils, epithelial, keratinocytes, and sebocytes in fibrotic LS *vs*. HC skin samples. The following cells are overexpressed in fibrotic LS *vs*. HC: CD4+ Tcm (*Q*=2.88E-03), central dendritic cells (*Q*=3.52E-02), eosinophils (*Q*=2.88E-03), epithelial (*Q*=3.68E-03), keratinocytes (*Q*=3.68E-03), and sebocytes (*Q*=5.12E-03).

	Comparison	Up	Down	Total
	LS-HC	173	160	333
**Activity Status**	Active-HC	276	246	522
	Inactive-HC	303	71	374
**Clinical subtype**	Circumscribed-HC	57	65	122
	Generalized-HC	140	30	170
	Linear-HC	384	260	644
**Clinical Stage**	Inflammatory-HC	109	148	257
	Fibrotic-HC	160	225	385

Table S1. Differentially Expressed Genes Summary.

Gene	Protein Encoded	Chr.	LogFC	Q value*	**Functional Annotation
IGHG1	Immunoglobulin heavy constant gamma 1	14	4.40433024	0.01892226	Antibody-dependent cellular cytotoxicity, adaptive immune response, phagocytosis, recognition, phagocytosis, engulfment, complement activation, classical pathway, antibacterial humoral response, defense response to bacterium, innate immune response, B cell receptor signaling pathway, positive regulation of B cell activation, complement-dependent cytotoxicity.
PLIN4	Perilipin 4	19	2.82815388	0.0344082	Involved in peroxisome proliferator-activated receptor (PPAR) signaling pathway.
NUTM2B	NUT family member 2B	10	2.77967703	0.0144482	Nuclear Testis protein, N-terminal, Nuclear Testis protein/FAM22.
AC009533.1	Pseudogene	12	2.22957223	0.00521699	Unknown function.
FO538757.1	Pseudogene	1	2.10030104	0.02065243	Unknown function.
FLG	Filaggrin	1	2.09940778	0.0259487	Peptide cross-linking, keratinocyte differentiation, establishment of skin barrier.

MYO1G	Myosin IG	7	1.98955926	0.04380642	T cell mediated immunity, exocytosis, actin filament organization, vesicle transport along actin filament, cell-substrate adhesion, Fc-gamma receptor signaling pathway involved in phagocytosis, cell gliding, T cell migration.
GOLGA8B	Golgin A8 family member B	15	1.97427688	0.04566786	Golgi organization, spindle assembly.
HLA-DQB1	Major histocompatibility complex, class II, DQ beta 1	6	1.9507629	0.02486262	Adaptive immune response, antigen processing and presentation of exogenous peptide antigen via MHC class I, TAP-independent, peptide antigen assembly with MHC class II protein complex, humoral immune response, leukocyte activation, T cell receptor signaling pathway, positive regulation of T cell activation, IFN-gamma-mediated signaling pathway.
ACAP1	ArfGAP with coiled-coil, ankyrin repeat and PH domains 1	17	1.93166256	0.03800936	Protein transport.

NLRC5	NLR family CARD domain containing 5	16	1.9085496	0.00259722	Response to bacterium and virus, negative regulation of NFKB, intracellular signal transduction, regulation of kinase activity, innate immune response, positive regulation of MHC class I, positive regulation of transcription from RNA polymerase II promoter, positive regulation of IFN-gamma signaling pathway, positive regulation of type I IFN pathway.
AC126755.1	Pseudogene	16	1.88909377	0.00172079	Unknown function.
CCDC88B	Coiled-coil domain containing 88B	11	1.86641864	0.03910425	Positive regulation of cytokine production, cytoskeleton-dependent intracellular transport, cytoplasmic microtubule organization, positive regulation of T cell proliferation/ activation.
HLA-DQA1	Major histocompatibility complex, class II, DQ alpha 1	6	1.84924421	0.00826441	Adaptive immune response, antigen processing and presentation of peptide or polysaccharide antigen via MHC class II, positive regulation of T cell activation.

MST1	Macrophage stimulating 1	3	1.75588489	0.01188071	Proteolysis, spermatogenesis, embryo implantation, regulation of macrophage chemotaxis, positive regulation of mammary gland epithelial cell proliferation, histone H2A-S139 phosphorylation, regulation of JAK-STAT cascade, cellular response to hypoxia, negative regulation of epithelial cell apoptotic process, regulation of cAMP-dependent protein kinase activity.
SPTBN2	Spectrin beta, non-erythrocytic 2	11	1.72489078	0.01912415	Cell communication, synapse assembly, vesicle-mediated transport, cerebellar Purkinje cell layer morphogenesis, signaling, actin cytoskeleton organization, adult behavior, multicellular organism growth, response to stimulus, cellular localization, actin filament capping.

IFI27	Interferon alpha inducible protein 27	14	1.70447515	0.04692332	Apoptotic process, proteasome-mediated ubiquitin-dependent protein catabolic process, modulation by host of viral genome replication, innate immune response, regulation of protein export from nucleus, defense response to virus, type I IFN signaling pathway, pyroptosis, protein K48-linked ubiquitination, apoptotic signaling pathway, extrinsic apoptotic signaling pathway.
CORO1A	Coronin 1A	16	1.66731389	0.04723006	Phagolysosome assembly, calcium ion transport, phagocytosis, actin filament organization, regulation of cell shape, epithelial cell migration, leukocyte chemotaxis, nerve growth factor signaling pathway, positive regulation of T cell proliferation, T cell homeostasis, natural killer cell degranulation, negative regulation of neuron apoptotic process, innate immune response, cellular response to interleukin-4, thymocyte migration, positive regulation of T cell migration.
GOLGA6L9	Golgin A6 family-like 9	15	1.65985337	0.0040774	Protein binding.

PLCH2	Phospholipase C eta 2	1	1.64171894	0.0180417	Lipid catabolic process, intracellular signal transduction, inositol phosphate metabolic process, phosphatidylinositol metabolic process, phosphatidylinositol-mediated signaling, release of sequestered calcium ion into cytosol.
CIITA	Class II major histocompatibility complex transactivator	16	1.64026587	0.00579872	Immune response, aging, negative regulation of collagen biosynthetic process, response to IFN-gamma, positive regulation of MHC class I and II process, negative regulation of viral entry into host cell, response to antibiotic, cellular response to electrical stimulus, cellular response to IFN-gamma, cellular response to exogenous dsRNA.
WHRN	Whirlin	9	1.6331297	0.00490783	Retina homeostasis, sensory perception of sound, positive regulation of gene expression, cerebellar Purkinje cell layer formation, establishment of protein localization, detection of mechanical stimulus involved in sensory perception of sound, sensory perception of light stimulus, establishment of localization in cell.

IRF7	Interferon regulatory factor 7	11	1.63140572	0.01768824	Negative regulation of transcription from RNA polymerase II promoter, regulation of adaptive immune response, cellular response to DNA damage stimulus, response to virus, immunoglobulin mediated immune response, establishment of viral latency, positive regulation of type I IFN production, regulation of MyD88-dependent toll-like receptor signaling pathway, MDA-5 signaling pathway, innate immune response, regulation of monocyte differentiation, defense response to virus, negative regulation of macrophage apoptotic process.
HIVEP3	Human immunodeficiency virus type I enhancer binding protein 3	1	1.61299129	0.01444834	Regulation of transcription from RNA polymerase II promoter, skeletal muscle cell differentiation, positive regulation of transcription, DNA-templated.
STK10	Serine/threonine kinase 10	5	1.61172716	0.00602534	Protein phosphorylation, cell cycle, protein autophosphorylation, lymphocyte aggregation, regulation of lymphocyte migration.

CSPG4	Chondroitin sulfate proteoglycan 4	15	1.60412209	0.03800936	Angiogenesis, cell migration, cell proliferation, intracellular signal transduction, positive regulation of MAPK cascade, platelet-derived growth factor receptor signaling pathway, tissue remodeling, positive regulation of peptidyl-tyrosine phosphorylation, ruffle assembly.
CDHR1	Cadherin related family member 1	10	1.60242086	0.038214	Cell adhesion.
MYO1F	Myosin IF	19	1.59024041	0.02018339	Actin filament organization, vesicle transport along actin filament.
PSD4	Pleckstrin and Sec7 domain containing 4	2	1.58066969	0.0099719	Regulation of ARF protein signal transduction.
ZNF767P	Zinc finger family member 767, pseudogene	7	1.57354032	0.00334364	Unknown function.
AGAP9	ArfGAP with GTPase domain, ankyrin repeat and PH domain 9	10	1.56280258	0.00605875	Activation of GTPase activity.
PABPC1L	Poly(A) binding protein cytoplasmic 1 like	20	1.56268483	0.00872253	Oocyte maturation, chromatin remodeling, mRNA polyadenylation, positive regulation of gene expression, epigenetic, nucleus localization.
LINC01002	Long intergenic non-protein coding RNA 1002	19	1.56146461	0.02054978	Unknown function.

ABCA7	ATP binding cassette subfamily A member 7	19	1.54383535	0.00350908	Lipid transport, phagocytosis, regulation of lipid metabolic process, cholesterol efflux, phospholipid efflux, beta-amyloid formation, apolipoprotein A-I-mediated signaling pathway, negative regulation of MAPK cascade, negative regulation of endocytosis, positive regulation of phagocytosis, positive regulation of Eagap9RK1 and ERK2 cascade, negative regulation of PERK-mediated unfolded protein response.

RASGRP1	RAS guanyl releasing protein 1	15	1.51305917	0.02798622	Positive regulation of protein phosphorylation, inflammatory response to antigenic stimulus, signal transduction, natural killer cell activation, differentiation, cytotoxicity, cell differentiation, positive regulation of IFN-gamma production, positive regulation of TNF production, positive regulation of T cell differentiation, proliferation/activation, B cell proliferation/activation, mast cell degranulation, positive regulation of MAP kinase, GTPase activity, positive regulation of JNK cascade, positive regulation of Ras protein signal transduction, regulation of ERK1 and ERK2 cascade, positive regulation of ERK1 and ERK2 cascade, activation of GTPase activity.
EVPL	Envoplakin	17	1.4933874	0.03322252	Regulation of antibacterial peptide production, epidermis development, peptide cross-linking, keratinocyte differentiation, keratinization, wound healing, intermediate filament cytoskeleton organization.

TAP2	Transporter 2, ATP binding cassette subfamily B member	6	1.49283203	0.02285849	Positive regulation of T cell mediated cytotoxicity, response to molecule of bacterial origin, adaptive immune response, antigen processing and presentation of exogenous protein antigen via MHC class Ib, TAP-dependent.
ARHGAP30	Rho GTPase activating protein 30	1	1.47496849	0.01837944	Signal transduction, small GTPase mediated signal transduction, regulation of small GTPase mediated signal transduction.
C22orf39	Chromosome 22 open reading frame 39	22	1.46965881	0.00969855	Protein binding.
ARHGAP33	Rho GTPase activating protein 33	19	1.46842532	0.03458076	Signal transduction, small GTPase mediated signal transduction, response to toxic substance, protein transport, regulation of dendritic spine morphogenesis.
NUP210	Nucleoporin 210	3	1.4655526	0.04050076	Nucleocytoplasmic transport, protein transport, mRNA transport.
TAP1	Transporter 1, ATP binding cassette subfamily B member	6	1.46502225	0.0048971	Adaptive immune response, antigen processing and presentation of exogenous peptide antigen via MHC class I, TAP-dependent, cytosol to ER transport, transmembrane transport.
AC139495.3	Pseudogene	5	1.46195103	0.00259722	Unknown function.

GBP3	Guanylate binding protein 3	1	1.44784857	0.038214	Defense response to bacterium, innate immune response, defense response to virus, cytolysis in other organism, cellular response to lipopolysaccharide, cellular response to IFN-gamma, cellular response to interleukin-1, cellular response to TNF.
TNK2	Tyrosine kinase non receptor 2	3	1.43864081	0.00101986	Protein phosphorylation, endocytosis, signal transduction, cell surface receptor signaling pathway, tyrosine kinase signaling pathway, small GTPase mediated signal transduction, phosphorylation, cell differentiation, innate immune response, positive regulation of peptidyl-tyrosine phosphorylation, regulation of clathrin-dependent endocytosis.
EPHB6	EPH receptor B6	7	1.43612263	0.01050316	Type IV hypersensitivity, protein phosphorylation, transmembrane receptor protein tyrosine kinase signaling pathway, axon guidance, central nervous system projection neuron axonogenesis, positive regulation of protein binding, positive regulation of kinase activity, ephrin receptor signaling pathway, activated T cell proliferation, positive regulation of T cell costimulation.

C5orf66	Chromosome 5 open reading frame 66	5	1.43053979	0.04474797	Unknown function.
CELSR2	Cadherin EGF LAG seven-pass G-type receptor 2	1	1.41068723	0.02010171	Neuron migration, G-protein coupled receptor signaling pathway, Wnt signaling pathway, regulation of cell-cell adhesion, regulation of protein localization, dendrite morphogenesis, planar cell polarity pathway, cilium assembly, motor neuron migration, cell-cell adhesion.
EPPK1	Epiplakin 1	8	1.41039087	0.04302081	Negative regulation of keratinocyte proliferation, negative regulation of cell migration, wound healing, intermediate filament cytoskeleton organization, intermediate filament organization, intermediate filament bundle assembly, negative regulation of epithelial cell proliferation, negative regulation of keratinocyte migration, negative regulation of wound healing, regulation of epithelium regeneration.
FCHSD1	FCH and double SH3 domains 1	5	1.40733822	0.00280309	Neuromuscular synaptic transmission, regulation of actin filament polymerization, positive regulation of actin filament polymerization, multi-organism membrane organization, membrane organization.

Table S2. Top 50 Upregulated Genes in LS *versus* HC. *P-values were corrected by multiple hypothesis testing to give the Q value by the Benjamini-Hochberg method. **Functional Annotation is adapted from Database for Annotation, Visualization and Integrated Discovery (DAVID). Chr; chromosome.

Gene	Protein Encoded	Chr.	LogFC	Q value*	**Functional Annotation
FP671120.3	*pseudogene*	21	-3.553024539	0.0025304	Unknown function.
FP236383.3	*pseudogene*	21	-3.552276398	0.0025304	Unknown function.
MT-ND4L	mitochondrially encoded NADH:ubiquinone oxidoreductase core subunit 4L	M	-3.227953031	0.0033436	Mitochondrial electron transport, NADH to ubiquinone, aerobic respiration, ATP synthesis coupled electron transport, mitochondrial ATP synthesis coupled proton transport.
MT-ATP8	mitochondrially encoded ATP synthase membrane subunit 8	M	-3.083713758	0.004757585554	ATP synthesis coupled proton transport, mitochondrial ATP synthesis coupled proton transport, hydrogen ion transmembrane transport.
MTATP6P1	mitochondrially encoded ATP synthase 6 pseudogene 1	1	-2.835706098	0.00209020	Unknown function.

GREM1	gremlin 1, DAN family BMP antagonist	15	-2.8125244	0.00072714	Cell morphogenesis, sprouting angiogenesis, transcription regulation in heart and kidney development, signal transduction, cell proliferation, limb and organ morphogenesis, bone and cartilage growth regulation, angiogenesis, DNA transcription, and cell differentiation. It also involves various negative regulations in bone remodeling and Wnt signaling, and positive influences on NF-kappaB activity, telomerase activity, and cardiac muscle development.
MT-ND3	mitochondrially encoded NADH:ubiquinone oxidoreductase core subunit 3	M	-2.788085962	0.00372906	Mitochondrial electron transport, NADH to ubiquinone, response to oxidative stress, aerobic respiration, response to light intensity, mitochondrial ATP synthesis coupled proton transport, cellular response to glucocorticoid stimulus.

MT-ND1	mitochondrially encoded NADH:ubiquinone oxidoreductase core subunit 1	M	-2.785189357	0.00372906	Mitochondrial electron transport, NADH to ubiquinone, aerobic respiration, mitochondrial respiratory chain complex I assembly, mitochondrial ATP synthesis coupled proton transport.
MT-RNR1	mitochondrially encoded 12S RNA	M	-2.6571596	0.00304977	Osteoblast differentiation, regulation of transcription from RNA polymerase II promoter, activation of protein kinase activity, osteoblast proliferation, regulation of carbohydrate utilization, skeletal muscle tissue growth, positive regulation of protein serine/threonine kinase activity, purine-containing compound biosynthetic process, negative regulation of phosphatidylinositol-3,4,5-trisphosphate 5-phosphatase activity.

MT-ATP6	mitochondrially encoded ATP synthase membrane subunit 6	M	-2.6340776	0.00350908	ATP biosynthetic process, ion transport, aging, ATP synthesis coupled proton transport, mitochondrial ATP synthesis coupled proton transport, response to hyperoxia, hydrogen ion transmembrane transport.
FP671120.4	*pseudogene*	21	-2.6175081	0.00334364	Unknown function.
MT-ND2	mitochondrially encoded NADH:ubiquinone oxidoreductase core subunit 2	M	-2.6081642	0.00350908	Mitochondrial electron transport, NADH to ubiquinone, aerobic respiration, mitochondrial respiratory chain complex I assembly, mitochondrial ATP synthesis coupled proton transport, reactive oxygen species metabolic process.
MT-CO2	mitochondrially encoded cytochrome c oxidase II	M	-2.604124968	0.00334364	Mitochondrial electron transport, cytochrome c to oxygen, lactation, response to cold, positive regulation of hydrogen peroxide biosynthetic process, positive regulation of necrotic cell death, ATP synthesis coupled electron transport, cellular respiration, hydrogen ion transmembrane transport, positive regulation of ATP biosynthetic process.

MT-CO3	mitochondrially encoded cytochrome c oxidase III	M	-2.596132919	0.00304977	Mitochondrial electron transport, cytochrome c to oxygen, respiratory chain complex IV assembly, aerobic respiration, aerobic electron transport chain, cellular respiration, hydrogen ion transmembrane transport.
LYVE1	lymphatic vessel endothelial hyaluronan receptor 1	11	-2.577923585	0.00200829	Positive regulation of cellular extravasation, glycosaminoglycan catabolic process, receptor-mediated endocytosis, cell adhesion, cell-matrix adhesion, response to wounding, anatomical structure morphogenesis, hyaluronan catabolic process.
RPPH1	ribonuclease P RNA component H1	14	-2.554313108	0.00410476	tRNA processing.
MT-CYB	mitochondrially encoded cytochrome b	M	-2.530899892	0.00372906	Oxidative phosphorylation, mitochondrial electron transport, ubiquinol to cytochrome c, respiratory electron transport chain, cellular respiration, hydrogen ion transmembrane transport.

OSR2	odd-skipped related transciption factor 2	8	-2.513775896	0.00082034	Negative regulation of transcription from RNA polymerase II promoter, urogenital system development, metanephros development, mesonephros development, chondrocyte differentiation, regulation of transcription from RNA polymerase II promoter, positive regulation of cell proliferation, embryo development, embryo development ending in birth or egg hatching, positive regulation of gene expression, cell differentiation, positive regulation of bone mineralization, osteoblast proliferation, embryonic forelimb morphogenesis, embryonic hindlimb morphogenesis, embryonic skeletal limb joint morphogenesis, middle ear morphogenesis, odontogenesis, embryonic digit morphogenesis, positive regulation of transcription, DNA-templated, positive regulation of transcription from RNA polymerase II

					promoter, embryonic skeletal system morphogenesis, positive regulation of epithelial cell proliferation, palate development, embryonic skeletal joint morphogenesis, head development, bone morphogenesis, eyelid development in camera-type eye, stem cell proliferation, embryonic skeletal joint development, positive regulation of stem cell proliferation.
OGN	osteoglycin	9	-2.505627767	0.00094155	Signal transduction, negative regulation of smooth muscle cell proliferation, bone development.
MT-CO1	mitochondrially encoded cytochrome c oxidase I	M	-2.486178717	0.00372906	Oxidative phosphorylation, mitochondrial electron transport, cytochrome c to oxygen, response to oxidative stress, aging, aerobic respiration, electron transport coupled proton transport, cerebellum development, respiratory electron transport chain, cellular respiration, response to copper ion, response to electrical stimulus.

SCARA5	scavenger receptor class A member 5	8	-2.471208016	0.01470036	Cellular iron ion homeostasis, endocytosis, cellular response to heat, iron ion transmembrane transport, protein homo-trimerization.
ACTG2	actin, gamma 2, smooth muscle, enteric	2	-2.386456917	0.00623616	Positive regulation of gene expression, mesenchyme migration.
MT-ND4	mitochondrially encoded NADH:ubiquinone oxidoreductase core subunit 4	M	-2.381827178	0.00411620	Response to hypoxia, in utero embryonic development, mitochondrial electron transport, NADH to ubiquinone, aging, aerobic respiration, electron transport coupled proton transport, cerebellum development, mitochondrial respiratory chain complex I assembly, response to nicotine, ATP synthesis coupled electron transport, mitochondrial ATP synthesis coupled proton transport, response to ethanol.
CADPS	calcium dependent secretion activator	3	-2.316239779	0.00334364	Exocytosis, protein transport, synaptic vesicle exocytosis, positive regulation of exocytosis, dense core granule exocytosis.

MT-ND6	mitochondrially encoded NADH:ubiquinone oxidoreductase core subunit 6	M	-2.315155235	0.00553023	Mitochondrial electron transport, NADH to ubiquinone, aerobic respiration, mitochondrial respiratory chain complex I assembly, response to nicotine, response to cocaine, response to hydrogen peroxide, mitochondrial ATP synthesis coupled proton transport.
TSPAN8	tetraspanin 8	12	-2.284808449	0.03474238	Spermatogenesis, regulation of gene expression, negative regulation of blood coagulation.
GSTM5	glutathione S-transferase mu 5	1	-2.274825182	0.00380091	Glutathione metabolic process, glutathione derivative biosynthetic process.
MT-ND5	mitochondrially encoded NADH:ubiquinone oxidoreductase core subunit 5	M	-2.203962196	0.00416067	Response to hypoxia, mitochondrial electron transport, NADH to ubiquinone, aerobic respiration, response to organonitrogen compound, electron transport coupled proton transport, mitochondrial respiratory chain complex I assembly, response to hydrogen peroxide, ATP synthesis coupled electron transport, mitochondrial ATP synthesis coupled proton transport.

CNN1	calponin 1	19	-2.143986336	0.01016240	Regulation of smooth muscle contraction, actin filament organization, actomyosin structure organization, negative regulation of vascular smooth muscle cell proliferation.
DCN	decorin	12	-2.13756741	0.00513301	Animal organ morphogenesis, positive regulation of autophagy, negative regulation of endothelial cell migration, positive regulation of phosphatidylinositol 3-kinase signaling, positive regulation of macroautophagy, negative regulation of angiogenesis, peptide cross-linking via chondroitin 4-sulfate glycosaminoglycan, positive regulation of transcription from RNA polymerase II promoter, positive regulation of protein kinase B signaling, positive regulation of mitochondrial depolarization, positive regulation of mitochondrial fission, negative regulation of vascular endothelial growth factor signaling pathway.

RN7SL1	RNA, 7SL, cytoplasmic 1	14	-2.088678124	0.00334364	SRP-dependent cotranslational protein targeting to membrane, signal sequence recognition.
AL139099.4	*pseudogene*	14	-2.075036237	0.00342550	Unknown function.
RNA5-8SN4	RNA, 5.8S ribosomal N4	21	-2.051010137	0.00623616	Involved in ribosome biogenesis in eukaryotes.
CAMK2N1	calcium/calmodulin dependent protein kinase II inhibitor 1	1	-1.994929724	0.00082034	Negative regulation of protein kinase activity, long-term memory, positive regulation of inflammatory response.
APOLD1	apolipoprotein L domain containing 1	12	-1.979430748	0.01055807	Angiogenesis, lipid transport, cell differentiation, lipoprotein metabolic process, regulation of endothelial cell differentiation.

LGR5	leucine rich repeat containing G protein-coupled receptor 5	12	-1.971065683	0.03140407	Hair follicle development, G-protein coupled receptor signaling pathway, adenylate cyclase-activating G-protein coupled receptor signaling pathway, activation of adenylate cyclase activity, hormone-mediated signaling pathway, oocyte differentiation, regulation of cell proliferation, inner ear development, positive regulation of canonical Wnt signaling pathway, epithelial cell proliferation involved in renal tubule morphogenesis.
RF00100	*pseudogene*	6	-1.967100084	0.00651481	Unknown function.
MSMO1	methylsterol monooxygenase 1	4	-1.948276783	0.02162106	Fatty acid metabolic process, cholesterol biosynthetic process, steroid metabolic process, lipid biosynthetic process, sterol biosynthetic process.
IGSF10	immunoglobulin superfamily member 10	3	-1.93819325	0.00958294	Ossification, cell differentiation, regulation of neuron migration.
RN7SK	RNA, 7SK small nuclear	6	-1.9306696	0.00682384	Negative regulation of transcription elongation from RNA polymerase II promoter.
RNA5S9	RNA, 5S ribosomal 9	1	-1.922731095	0.02483277	Ribosome biogenesis in eukaryotes.
FP236383.2	*pseudogene*	21	-1.905493854	0.00343207	Unknown function.

PDK4	pyruvate dehydrogenase kinase 4	7	-1.868285164	0.02285848	Glucose metabolic process, protein phosphorylation, regulation of pH, insulin receptor signaling pathway, cellular response to starvation, regulation of acetyl-CoA biosynthetic process from pyruvate, regulation of cellular ketone metabolic process, regulation of glucose metabolic process, phosphorylation, regulation of fatty acid biosynthetic process, glucose homeostasis, response to starvation, regulation of bone resorption, regulation of fatty acid oxidation, cellular response to fatty acid, reactive oxygen species metabolic process, negative regulation of anoikis.
AC133134.1	*pseudogene*	5	-1.833668842	0.00253044	Unknown function.
AL078639.1	*pseudogene*	X	-1.831012849	0.03017090	Unknown function.
OIP5-AS1	OIP5 antisense RNA 1	15	-1.809870006	0.01106114	Unknown function.
SNORD3B-2	small nucleolar RNA, C/D box 3B-2	17	-1.809624348	0.02110415	Ribosome biogenesis in eukaryotes.
RNA5-8SN1	RNA, 5.8S ribosomal N1	21	-1.801726005	0.02180451	Ribosome biogenesis in eukaryotes.

CXCL12	C-X-C motif chemokine ligand 12	10	-1.758990016	0.01349246	Responses to environmental stimuli like hypoxia, heat, radiation, and pathogens, as well as fundamental biological processes such as neuron migration, endothelial cell proliferation, immune and defense responses, and cell adhesion. Key activities also include signaling pathways (G-protein coupled receptor, chemokine), axon guidance, blood circulation, actin dynamics, locomotion, and various forms of cell chemotaxis. Additionally, it involves the regulation of apoptosis, leukocyte activity, calcium ion homeostasis, dopamine secretion, and T cell migration, along with roles in pain perception, organ regeneration, and neuron differentiation.

FBLN1	fibulin 1	22	-1.732129796	0.03255367	Negative regulation of protein phosphorylation, cell adhesion, and ERK1/ERK2 signaling cascades, as well as the suppression of transforming growth factor beta production, cell motility, stem cell proliferation, and virus-induced host cell transformation. Additionally, it emphasizes positive regulation of gene expression, fibroblast proliferation, and substrate-dependent cell migration. Key processes also include embryo implantation, extracellular matrix organization, blood coagulation, fibrin clot formation, and cell attachment to substrates.

Table S3. Top 50 Downregulated Genes in LS *versus* HC. *P-values were corrected by multiple hypothesis testing to give the Q value by the Benjamini-Hochberg method. **Functional Annotation is adapted from Database for Annotation, Visualization and Integrated Discovery (DAVID). Chr; chromosome.

Gene	Protein Encoded	Chr.	LogFC	Q value*	**Functional Annotation
IGHG1	Immunoglobulin heavy constant gamma 1	14	4.974925336	0.0085	Antibody-dependent cellular cytotoxicity, adaptive immune response, phagocytosis, recognition, phagocytosis, engulfment, complement activation, classical pathway, antibacterial humoral response, defense response to bacterium, innate immune response, B cell receptor signaling pathway, positive regulation of B cell activation, complement-dependent cytotoxicity.
IGLC2	immunoglobulin lambda constant 2	22	4.234596119	0.0301	Phagocytosis, recognition, phagocytosis, engulfment, complement activation, classical pathway, immunoglobulin mediated immune response, defense response to bacterium, innate immune response, B cell receptor signaling pathway, and positive regulation of B cell activation.

IGKC	immunoglobulin kappa constant	2	3.85170274	0.0313	retina homeostasis, adaptive immune response, phagocytosis, recognition, phagocytosis, engulfment, immune response, complement activation, classical pathway, immunoglobulin mediated immune response, defense response to bacterium, innate immune response, B cell receptor signaling pathway, positive regulation of B cell activation,
ITGAL	integrin subunit alpha L	16	2.838966694	0.0349	phagocytosis, recognition, phagocytosis, engulfment, complement activation, classical pathway, immunoglobulin mediated immune response, defense response to bacterium, innate immune response, B cell receptor signaling pathway, positive regulation of B cell activation,
PLIN4	Perilipin 4	19	2.779804922	0.0350	Involved in peroxisome proliferator-activated receptor (PPAR) signaling pathway.
NUTM2B	NUT family member 2B	10	2.734385886	0.0108	Nuclear Testis protein, N-terminal, Nuclear Testis protein/FAM22.
FLG	Filaggrin	1	2.492590644	0.0067	Peptide cross-linking, keratinocyte differentiation, establishment of skin barrier.

MYO1G	Myosin IG	7	2.459840252	0.0068	T cell mediated immunity, exocytosis, actin filament organization, vesicle transport along actin filament, cell-substrate adhesion, Fc-gamma receptor signaling pathway involved in phagocytosis, cell gliding, T cell migration.
HLA-DQA1	Major histocompatibility complex, class II, DQ beta 1	6	2.374299857	0.0009	Adaptive immune response, antigen processing and presentation of exogenous peptide antigen via MHC class I, TAP-independent, peptide antigen assembly with MHC class II protein complex, humoral immune response, leukocyte activation, T cell receptor signaling pathway, positive regulation of T cell activation, IFN-gamma-mediated signaling pathway.
MAP4K1	mitogen-activated protein kinase kinase kinase kinase 1	19	2.351168836	0.0339	Protein phosphorylation, JNK cascade, cell proliferation, peptidyl-serine phosphorylation, intracellular signal transduction, positive regulation of MAPK cascade, protein autophosphorylation, cellular response to phorbol 13-acetate 12-myristate.

HLA-DQB1	major histocompatibility complex, class II, DQ beta 1	6	2.314325071	0.0099	Adaptive immune response, antigen processing and presentation of exogenous peptide antigen via MHC class I, TAP-independent, peptide antigen assembly with MHC class II protein complex, humoral immune response, leukocyte activation, T cell receptor signaling pathway, positive regulation of T cell activation, IFN-gamma-mediated signaling pathway.
ACAP1	ArfGAP with coiled-coil, ankyrin repeat and PH domains 1	17	2.284634556	0.0085	Protein transport.
NLRC5	NLR family CARD domain containing 5	16	2.19369622	0.0003	Response to bacterium and virus, negative regulation of NFKB, intracellular signal transduction, regulation of kinase activity, innate immune response, positive regulation of MHC class I, positive regulation of transcription from RNA polymerase II promoter, positive regulation of IFN-gamma signaling pathway, positive regulation of type I IFN pathway.

CORO1A	coronin 1A	16	2.174646443	0.0067	Phagolysosome assembly, calcium ion transport, phagocytosis, actin filament organization, regulation of cell shape, epithelial cell migration, leukocyte chemotaxis, nerve growth factor signaling pathway, positive regulation of T cell proliferation, T cell homeostasis, natural killer cell degranulation, negative regulation of neuron apoptotic process, innate immune response, cellular response to interleukin-4, thymocyte migration, positive regulation of T cell migration.
IFI27	interferon alpha inducible protein 27	14	2.173084516	0.0039	Apoptotic process, proteasome-mediated ubiquitin-dependent protein catabolic process, modulation by host of viral genome replication, innate immune response, regulation of protein export from nucleus, defense response to virus, type I IFN signaling pathway, pyroptosis, protein K48-linked ubiquitination, apoptotic signaling pathway, extrinsic apoptotic signaling pathway.
AC009533.1	pseudogene	12	2.15251742	0.0072	Unknown function.

PSTPIP1	proline-serine-threonine phosphatase interacting protein 1	15	2.14127869	0.0434	endocytosis, inflammatory response, cell adhesion,signal transduction, actin filament polymerization,innate immune response
FO538757.1	pseudogene	1	2.135976846	0.0174	Unknown function.
CCDC88B	coiled-coil domain containing 88B	11	2.105502579	0.0186	Positive regulation of cytokine production, cytoskeleton-dependent intracellular transport, cytoplasmic microtubule organization, positive regulation of T cell proliferation/ activation.
GBP4	guanylate binding protein 4	1	2.080364828	0.0487	defense response to protozoan, defense response to Gram-positive bacterium, cellular response to interferon-gamma,
SP140	SP140 nuclear body protein	2	2.039486761	0.0325	regulation of transcription from RNA polymerase II promoter, defense response,
IRF1	interferon regulatory factor 1	5	2.010012436	0.0173	Immune processes, including adaptive and innate immune responses, and the regulation of transcription related to RNA polymerase II. Includes apoptotic processes, cell proliferation, type I interferon production, and T cell differentiation and proliferation. Key pathways include toll-like receptor signaling, interferon-mediated responses, and cellular

					reactions to viral defense and mechanical stimuli.
SBSN	suprabasin	19	2.001652906	0.0350	Extracellular exosome.

PIK3CD	phosphatidylinositol-4,5-bisphosphate 3-kinase catalytic subunit delta	1	1.957004241	0.0126	Natural killer cell differentiation, positive regulation of cytokine production, positive regulation of endothelial cell proliferation, adaptive immune response, mast cell chemotaxis, respiratory burst involved in defense response, protein phosphorylation, inflammatory response, immune response, signal transduction, positive regulation of endothelial cell migration, positive regulation of gene expression, T cell chemotaxis, phosphatidylinositol 3-kinase signaling, phosphorylation, cell migration, natural killer cell activation, B cell differentiation, T cell differentiation, positive regulation of cell migration, neutrophil chemotaxis, positive regulation of neutrophil apoptotic process, natural killer cell chemotaxis, B cell chemotaxis, phosphatidylinositol-3-phosphate biosynthetic process, positive regulation of cell migration by vascular endothelial growth factor signaling pathway, T cell activation, B cell activation, mast cell degranulation, protein kinase B signaling, innate immune response, positive regulation of

					angiogenesis, phosphatidylinositol phosphorylation, phosphatidylinositol-mediated signaling, T cell receptor signaling pathway, B cell receptor signaling pathway, positive regulation of protein kinase B signaling, mast cell differentiation, neutrophil extravasation, positive regulation of epithelial tube formation.
GOLGA8B	golgin A8 family member B	15	1.94766451	0.0398	Golgi organization, spindle assembly.

GBP1	guanylate binding protein 1	1	1.941246624	0.0223	negative regulation of interleukin-2 production, defense response to bacterium, defense response to protozoan, innate immune response, regulation of calcium-mediated signaling, negative regulation of T cell receptor signaling pathway, defense response to virus, cytolysis in other organism, negative regulation of ERK1 and ERK2 cascade, cellular response to cytokine stimulus, cellular response to interferon-gamma, cellular response to interleukin-1, cellular response to tumor necrosis factor, protein localization to vacuole, negative regulation of substrate adhesion-dependent cell spreading, regulation of protein localization to plasma membrane, negative regulation of protein localization to plasma membrane,

KRT1	keratin 1	12	1.93187412	0.0356	complement activation, lectin pathway, retina homeostasis, response to oxidative stress, peptide cross-linking, keratinization, fibrinolysis, intermediate filament organization, regulation of angiogenesis, negative regulation of inflammatory response, protein heterotetramerization, establishment of skin barrier, cornification,
ITGAX	integrin subunit alpha X	16	1.931423949	0.0338	Cell adhesion, cell-matrix adhesion, integrin-mediated signaling pathway, positive regulation of cell proliferation, animal organ morphogenesis, positive regulation of gene expression, extracellular matrix organization, positive regulation of cell migration, positive regulation of myelination, cell adhesion mediated by integrin, heterotypic cell-cell adhesion, positive regulation of angiogenesis, defense response to virus, cell-cell adhesion, positive regulation of endothelial tube morphogenesis.

IFI30	IFI30, lysosomal thiol reductase	19	1.903389138	0.0119	Antigen processing and presentation of exogenous peptide antigen via MHC class II, antigen processing and presentation of exogenous peptide antigen via MHC class I, negative regulation of fibroblast proliferation, protein stabilization.
STK10	serine/threonine kinase 10	5	1.901990307	0.0007	Protein phosphorylation, cell cycle, protein autophosphorylation, lymphocyte aggregation, regulation of lymphocyte migration.
MYO1F	myosin IF	19	1.886111093	0.0060	Actin filament organization, vesicle transport along actin filament.

CIITA	class II major histocompatibility complex transactivator	16	1.884076581	0.0017	Negative regulation of transcription from RNA polymerase II promoter, regulation of transcription, DNA-templated, inflammatory response, immune response, aging, phosphorylation, negative regulation of collagen biosynthetic process, response to interferon-gamma, positive regulation of MHC class I biosynthetic process, positive regulation of MHC class II biosynthetic process, negative regulation of transcription, DNA-templated, positive regulation of transcription, DNA-templated, positive regulation of transcription from RNA polymerase II promoter, negative regulation of viral entry into host cell, response to antibiotic, cellular response to electrical stimulus, cellular response to interferon-gamma, cellular response to exogenous dsRNA.

HLA-DRB5	major histocompatibility complex, class II, DR beta 5	6	1.871220941	0.0136	adaptive immune response, peptide antigen assembly with MHC class II protein complex, antigen processing and presentation of peptide or polysaccharide antigen via MHC class II, immune response, antigen processing and presentation, antigen processing and presentation of exogenous peptide antigen via MHC class II, positive regulation of immune response, positive regulation of T cell activation,
PARVG	parvin gamma	22	1.870952836	0.0396	Cell-matrix adhesion, establishment or maintenance of cell polarity, cell projection assembly, actin cytoskeleton organization, actin cytoskeleton reorganization, substrate adhesion-dependent cell spreading.
TAP2	transporter 2, ATP binding cassette subfamily B member	6	1.856601301	0.0026	Positive regulation of T cell mediated cytotoxicity, response to molecule of bacterial origin, adaptive immune response, antigen processing and presentation of exogenous protein antigen via MHC class Ib, TAP-dependent.

SPTBN2	spectrin beta, non-erythrocytic 2	11	1.843092788	0.0141	cell communication, synapse assembly, vesicle-mediated transport, cerebellar Purkinje cell layer morphogenesis, signaling, actin cytoskeleton organization, adult behavior, multicellular organism growth, response to stimulus, cellular localization, actin filament capping,
TAP1	transporter 1, ATP binding cassette subfamily B member	6	1.822458719	0.0003	Adaptive immune response, antigen processing and presentation of exogenous peptide antigen via MHC class I, TAP-dependent, cytosol to ER transport, transmembrane transport.
AC126755.1	pseudogene	16	1.8164113	0.0011	Unknown function.

RASGRP1	RAS guanyl releasing protein 1	15	1.814221051	0.0035	Positive regulation of protein phosphorylation, inflammatory response to antigenic stimulus, signal transduction, natural killer cell activation, differentiation, cytotoxicity, cell differentiation, positive regulation of IFN-gamma production, positive regulation of TNF production, positive regulation of T cell differentiation, proliferation/activation, B cell proliferation/activation, mast cell degranulation, positive regulation of MAP kinase, GTPase activity, positive regulation of JNK cascade, positive regulation of Ras protein signal transduction, regulation of ERK1 and ERK2 cascade, positive regulation of ERK1 and ERK2 cascade, activation of GTPase activity.

IRF7	interferon regulatory factor 7	11	1.812811368	0.0103	Negative regulation of transcription from RNA polymerase II promoter, regulation of adaptive immune response, cellular response to DNA damage stimulus, response to virus, immunoglobulin mediated immune response, establishment of viral latency, positive regulation of type I IFN production, regulation of MyD88-dependent toll-like receptor signaling pathway, MDA-5 signaling pathway, innate immune response, regulation of monocyte differentiation, defense response to virus, negative regulation of macrophage apoptotic process.
ARHGAP30	Rho GTPase activating protein 30	1	1.808435596	0.0021	Signal transduction, small GTPase mediated signal transduction, regulation of small GTPase mediated signal transduction.

PLCB2	phospholipase C beta 2	15	1.805522788	0.0281	detection of chemical stimulus involved in sensory perception of bitter taste, phospholipid metabolic process, G-protein coupled receptor signaling pathway, phospholipase C-activating G-protein coupled receptor signaling pathway, activation of phospholipase C activity, lipid catabolic process, intracellular signal transduction, phosphatidylinositol metabolic process, phosphatidylinositol-mediated signaling, release of sequestered calcium ion into cytosol,
WHRN	whirlin	9	1.804674441	0.0008	Retina homeostasis, sensory perception of sound, positive regulation of gene expression, cerebellar Purkinje cell layer formation, establishment of protein localization, detection of mechanical stimulus involved in sensory perception of sound, sensory perception of light stimulus, establishment of localization in cell.
PSD4	pleckstrin and Sec7 domain containing 4	2	1.793383001	0.0023	Regulation of ARF protein signal transduction.
HIVEP3	human immunodeficiency virus type I enhancer binding protein 3	1	1.775566661	0.0060	Regulation of transcription from RNA polymerase II promoter, skeletal muscle cell differentiation, positive regulation of transcription, DNA-templated.

NUP210	nucleoporin 210	3	1.771437949	0.0073	Nucleocytoplasmic transport, protein transport, mRNA transport.
HLA-DRB1	major histocompatibility complex, class II, DR beta 1	6	1.769444879	0.0020	adaptive immune response, peptide antigen assembly with MHC class II protein complex, antigen processing and presentation of peptide or polysaccharide antigen via MHC class II, immune response, antigen processing and presentation, antigen processing and presentation of exogenous peptide antigen via MHC class II, positive regulation of immune response, positive regulation of T cell activation,
STAT1	signal transducer and activator of transcription 1	2	1.750280062	0.0060	Pathways activated by bioactive peptides and responses to DNA damage, including apoptotic signaling. Examines the EGF, IFN alpha, IFN gamma, and p38 MAPK signaling pathways, as well as the anti-inflammatory effects of IL-10 and IL22 soluble receptor pathways. Involves the inhibition of cellular proliferation by Gleevec, PDGF, MAPKinase, and TPO signaling pathways.

MST1	macrophage stimulating 1	3	1.742260771	0.0108	Proteolysis, spermatogenesis, embryo implantation, regulation of macrophage chemotaxis, positive regulation of mammary gland epithelial cell proliferation, histone H2A-S139 phosphorylation, regulation of JAK-STAT cascade, cellular response to hypoxia, negative regulation of epithelial cell apoptotic process, regulation of cAMP-dependent protein kinase activity.
ITGA4	integrin subunit alpha 4	2	1.73965349	0.0417	Cells and Molecules involved in local acute inflammatory response, Adhesion and Diapedesis of Lymphocytes, Adhesion Molecules on Lymphocyte, Monocyte and its Surface Molecules,

Table S4. Top 50 Upregulated Genes in Active LS *versus* HC. *P-values were corrected by multiple hypothesis testing to give the Q value by the Benjamini-Hochberg method. **Functional Annotation is adapted from Database for Annotation, Visualization and Integrated Discovery (DAVID). Chr; chromosome.

Gene	Protein Encoded	Chr.	LogFC	Q value*	**Functional Annotation
FP671120.3	*pseudogene*	21	-3.646571091	0.0015	Unknown function
FP236383.3	*pseudogene*	21	-3.645231582	0.0015	Unknown function
MT-ND4L	mitochondrially encoded NADH:ubiquinone oxidoreductase core subunit 4L	M	-3.458502681	0.0020	Mitochondrial electron transport, NADH to ubiquinone, aerobic respiration, ATP synthesis coupled electron transport, mitochondrial ATP synthesis coupled proton transport.
MT-ATP8	mitochondrially encoded ATP synthase membrane subunit 8	M	-3.307877063	0.0032	ATP synthesis coupled proton transport, mitochondrial ATP synthesis coupled proton transport, hydrogen ion transmembrane transport.
SCARA5	scavenger receptor class A member 5	8	-3.221919862	0.0007	Cellular iron ion homeostasis, endocytosis, cellular response to heat, iron ion transmembrane transport, protein homo-trimerization.
MTATP6P1	mitochondrially encoded ATP synthase 6 pseudogene 1	1	-3.150357901	0.0010	Unknown function.
LYVE1	lymphatic vessel endothelial hyaluronan receptor 1	11	-3.113543637	6.81E-05	Positive regulation of cellular extravasation, glycosaminoglycan catabolic process, receptor-mediated endocytosis, cell adhesion, cell-matrix adhesion, response to wounding, anatomical structure morphogenesis, hyaluronan catabolic process.

FADS2	fatty acid desaturase 2	11	-3.10504311	0.0125	Lipid metabolic process, unsaturated fatty acid biosynthetic process, alpha-linolenic acid metabolic process, linoleic acid metabolic process
MT-ND3	mitochondrially encoded NADH:ubiquinone oxidoreductase core subunit 3	M	-3.037538703	0.0023	Mitochondrial electron transport, NADH to ubiquinone, response to oxidative stress, aerobic respiration, response to light intensity, mitochondrial ATP synthesis coupled proton transport, cellular response to glucocorticoid stimulus.

OSR2	odd-skipped related transciption factor 2	8	-3.035580303	1.99E-05	Negative regulation of transcription from RNA polymerase II promoter, urogenital system development, metanephros development, mesonephros development, chondrocyte differentiation, regulation of transcription from RNA polymerase II promoter, positive regulation of cell proliferation, embryo development, embryo development ending in birth or egg hatching, positive regulation of gene expression, cell differentiation, positive regulation of bone mineralization, osteoblast proliferation, embryonic forelimb morphogenesis, embryonic hindlimb morphogenesis, embryonic skeletal limb joint morphogenesis, middle ear morphogenesis, odontogenesis, embryonic digit morphogenesis, positive regulation of transcription, DNA-templated, positive regulation of transcription from RNA polymerase II promoter, embryonic skeletal system morphogenesis, positive regulation of epithelial cell proliferation, palate development, embryonic skeletal joint

					morphogenesis, head development, bone morphogenesis, eyelid development in camera-type eye, stem cell proliferation, embryonic skeletal joint development, positive regulation of stem cell proliferation.
OGN	osteoglycin	9	-3.007401528	1.99E-05	Signal transduction, negative regulation of smooth muscle cell proliferation, bone development.
TSPAN8	tetraspanin 8	12	-2.991836047	0.0040	Spermatogenesis, regulation of gene expression, negative regulation of blood coagulation.
MT-ND1	mitochondrially encoded NADH:ubiquinone oxidoreductase core subunit 1	M	-2.965259817	0.0023	Mitochondrial electron transport, NADH to ubiquinone, aerobic respiration, mitochondrial respiratory chain complex I assembly, mitochondrial ATP synthesis coupled proton transport.
GSTM5	glutathione S-transferase mu 5	1	-2.861492748	0.0005	Glutathione metabolic process, glutathione derivative biosynthetic process.
MT-ATP6	mitochondrially encoded ATP synthase membrane subunit 6	M	-2.856962542	0.0019	ATP biosynthetic process, ion transport, aging, ATP synthesis coupled proton transport, mitochondrial ATP synthesis coupled proton transport, response to hyperoxia, hydrogen ion transmembrane transport.
FP671120.4	pseudogene	21	-2.834611926	0.0019	Unknown

MT-ND2	mitochondrially encoded NADH:ubiquinone oxidoreductase core subunit 2	M	-2.819019619	0.0020	Mitochondrial electron transport, NADH to ubiquinone, aerobic respiration, mitochondrial respiratory chain complex I assembly, mitochondrial ATP synthesis coupled proton transport, reactive oxygen species metabolic process.
MT-CO3	mitochondrially encoded cytochrome c oxidase III	M	-2.802054326	0.0014	Mitochondrial electron transport, cytochrome c to oxygen, respiratory chain complex IV assembly, aerobic respiration, aerobic electron transport chain, cellular respiration, hydrogen ion transmembrane transport.
MT-CO2	mitochondrially encoded cytochrome c oxidase II	M	-2.79066339	0.0016	Mitochondrial electron transport, cytochrome c to oxygen, lactation, response to cold, positive regulation of hydrogen peroxide biosynthetic process, positive regulation of necrotic cell death, ATP synthesis coupled electron transport, cellular respiration, hydrogen ion transmembrane transport, positive regulation of ATP biosynthetic process.

GREM1	gremlin 1, DAN family BMP antagonist	15	-2.771473951	0.0004	Cell morphogenesis, sprouting angiogenesis, transcription regulation in heart and kidney development, signal transduction, cell proliferation, limb and organ morphogenesis, bone and cartilage growth regulation, angiogenesis, DNA transcription, and cell differentiation. It also involves various negative regulations in bone remodeling and Wnt signaling, and positive influences on NF-kappaB activity, telomerase activity, and cardiac muscle development.
MT-CYB	mitochondrially encoded cytochrome b	M	-2.743047271	0.0020	Oxidative phosphorylation, mitochondrial electron transport, ubiquinol to cytochrome c, respiratory electron transport chain, cellular respiration, hydrogen ion transmembrane transport.
RPPH1	ribonuclease P RNA component H1	14	-2.733687288	0.0024	tRNA processing.

LGR5	leucine rich repeat containing G protein-coupled receptor 5	12	-2.730114917	0.0024	Hair follicle development, G-protein coupled receptor signaling pathway, adenylate cyclase-activating G-protein coupled receptor signaling pathway, activation of adenylate cyclase activity, hormone-mediated signaling pathway, oocyte differentiation, regulation of cell proliferation, inner ear development, positive regulation of canonical Wnt signaling pathway, epithelial cell proliferation involved in renal tubule morphogenesis.
MT-RNR1	mitochondrially encoded 12S RNA	M	-2.706797503	0.0019	Osteoblast differentiation, regulation of transcription from RNA polymerase II promoter, activation of protein kinase activity, osteoblast proliferation, regulation of carbohydrate utilization, skeletal muscle tissue growth, positive regulation of protein serine/threonine kinase activity, purine-containing compound biosynthetic process, negative regulation of phosphatidylinositol-3,4,5-trisphosphate 5-phosphatase activity.

MT-CO1	mitochondrially encoded cytochrome c oxidase I	M	-2.691984969	0.0020	Oxidative phosphorylation, mitochondrial electron transport, cytochrome c to oxygen, response to oxidative stress, aging, aerobic respiration, electron transport coupled proton transport, cerebellum development, respiratory electron transport chain, cellular respiration, response to copper ion, response to electrical stimulus.
MT-ND4	mitochondrially encoded NADH:ubiquinone oxidoreductase core subunit 4	M	-2.653672965	0.0023	Mitochondrial electron transport, NADH to ubiquinone, aerobic respiration, ATP synthesis coupled electron transport, mitochondrial ATP synthesis coupled proton transport.
RNU4-2	RNA, U4 small nuclear 2	12	-2.597087218	0.0282	spliceosomal tri-snRNP complex assembly, formation of quadruple SL/U4/U5/U6 snRNP
ACTG2	actin, gamma 2, smooth muscle, enteric	2	-2.587728422	0.0023	Positive regulation of gene expression, mesenchyme migration.
CADPS	calcium dependent secretion activator	3	-2.579362776	0.0008	Exocytosis, protein transport, synaptic vesicle exocytosis, positive regulation of exocytosis, dense core granule exocytosis.

DCN	decorin	12	-2.574015565	0.0007	Animal organ morphogenesis, positive regulation of autophagy, negative regulation of endothelial cell migration, positive regulation of phosphatidylinositol 3-kinase signaling, positive regulation of macroautophagy, negative regulation of angiogenesis, peptide cross-linking via chondroitin 4-sulfate glycosaminoglycan, positive regulation of transcription from RNA polymerase II promoter, positive regulation of protein kinase B signaling, positive regulation of mitochondrial depolarization, positive regulation of mitochondrial fission, negative regulation of vascular endothelial growth factor signaling pathway.
AMY2A	amylase, alpha 2A (pancreatic)	1	-2.537078201	0.0497	carbohydrate metabolic process, carbohydrate catabolic process, polysaccharide digestion
MT-ND6	mitochondrially encoded NADH:ubiquinone oxidoreductase core subunit 6	M	-2.515755335	0.0035	Mitochondrial electron transport, NADH to ubiquinone, aerobic respiration, mitochondrial respiratory chain complex I assembly, response to nicotine, response to cocaine, response to hydrogen peroxide, mitochondrial ATP synthesis coupled proton transport.

IGSF10	immunoglobulin superfamily member 10	3	-2.445079925	0.0005	Ossification, cell differentiation, regulation of neuron migration.
MT-ND5	mitochondrially encoded NADH:ubiquinone oxidoreductase core subunit 5	M	-2.404723463	0.0026	Response to hypoxia, mitochondrial electron transport, NADH to ubiquinone, aerobic respiration, response to organonitrogen compound, electron transport coupled proton transport, mitochondrial respiratory chain complex I assembly, response to hydrogen peroxide, ATP synthesis coupled electron transport, mitochondrial ATP synthesis coupled proton transport.
FBLN1	fibulin 1	22	-2.374996448	0.0017	Negative regulation of protein phosphorylation, cell adhesion, and ERK1/ERK2 signaling cascades, as well as the suppression of transforming growth factor beta production, cell motility, stem cell proliferation, and virus-induced host cell transformation. Additionally, it emphasizes positive regulation of gene expression, fibroblast proliferation, and substrate-dependent cell migration. Key processes also include embryo implantation, extracellular matrix organization, blood coagulation, fibrin clot formation, and cell attachment to substrates.

RNA5-8SN4	RNA, 5.8S ribosomal N4	21	-2.371883971	0.0036	Involved in ribosome biogenesis in eukaryotes.
MSMO1	methylsterol monooxygenase 1	4	-2.332487017	0.0044	Fatty acid metabolic process, cholesterol biosynthetic process, steroid metabolic process, lipid biosynthetic process, sterol biosynthetic process.
APOLD1	apolipoprotein L domain containing 1	12	-2.310865438	0.0015	Angiogenesis, lipid transport, cell differentiation, lipoprotein metabolic process, regulation of endothelial cell differentiation.
AL078639.1	pseudogene	X	-2.290297858	0.0064	Unknown function
MGST1	microsomal glutathione S-transferase 1	12	-2.277955484	0.0153	Glutathione transport, cellular response to lipid hydroperoxide, cellular oxidant detoxification
RNA5S9	RNA, 5S ribosomal 9	1	-2.270402113	0.0116	Ribosome biogenesis in eukaryotes.
RN7SL1	RNA, 7SL, cytoplasmic 1	14	-2.242254054	0.0023	SRP-dependent cotranslational protein targeting to membrane, signal sequence recognition.
AL139099.4	pseudogene	14	-2.234194621	0.0023	Unknown function
CFD	complement factor D	19	-2.199549185	0.0034	Alternative Complement Pathway, Complement Pathway
CAMK2N1	calcium/calmodulin dependent protein kinase II inhibitor 1	1	-2.168515218	0.0003	Negative regulation of protein kinase activity, long-term memory, positive regulation of inflammatory response.
CNN1	calponin 1	19	-2.164740496	0.0104	Regulation of smooth muscle contraction, actin filament organization, actomyosin structure organization, negative regulation of vascular smooth muscle cell proliferation.

RNA5-8SN1	RNA, 5.8S ribosomal N1	21	-2.15522801	0.0112	Ribosome biogenesis in eukaryotes.
RF00100	pseudogene	6	-2.136252573	0.0037	Unknown function
SNORD116-19	small nucleolar RNA, C/D box 116-19	15	-2.13235729	0.0294	RNA processing
CREB3L1	cAMP responsive element binding protein 3 like 1	11	-2.131004694	0.0001	Negative regulation of transcription from RNA polymerase II promoter, osteoblast differentiation, regulation of transcription from RNA polymerase II promoter, negative regulation of gene expression, endoplasmic reticulum unfolded protein response, positive regulation of collagen biosynthetic process, negative regulation of fibroblast growth factor receptor signaling pathway, negative regulation of transcription, DNA-templated, positive regulation of transcription from RNA polymerase II promoter, extracellular matrix constituent secretion, negative regulation of endoplasmic reticulum stress-induced intrinsic apoptotic signaling pathway, negative regulation of sprouting angiogenesis, positive regulation of transcription from RNA polymerase II promoter in response to endoplasmic reticulum stress

Table S5. Top 50 Downregulated Genes in Active LS *versus* HC. *P-values were corrected by multiple hypothesis testing to give the Q value by the Benjamini-Hochberg method. **Functional Annotation is adapted from Database for Annotation, Visualization and Integrated Discovery (DAVID). Chr; chromosome.

Gene	Protein Encoded	Chr.	LogFC	Q value *	**Functional Annotation
HLA-DQA1	major histocompatibility complex, class II, DQ alpha 1	6	2.677202406	0.001683	Adaptive immune response, peptide antigen assembly with MHC class II protein complex, antigen processing and presentation of peptide or polysaccharide antigen via MHC class II, immune response, antigen processing and presentation, antigen processing and presentation of exogenous peptide antigen via MHC class II, positive regulation of immune response, positive regulation of T cell activation.
MAP4K1	mitogen-activated protein kinase kinase kinase kinase 1	19	2.467959003	0.002474	Protein phosphorylation, JNK cascade, cell proliferation, peptidyl-serine phosphorylation, intracellular signal transduction, positive regulation of MAPK cascade, protein autophosphorylation, cellular response to phorbol 13-acetate 12-myristate.

GBP1	guanylate binding protein 1	1	2.466359414	0.002474	Negative regulation of interleukin-2 production, defense response to bacterium, defense response to protozoan, innate immune response, regulation of calcium-mediated signaling, negative regulation of T cell receptor signaling pathway, defense response to virus, cytolysis in other organism, negative regulation of ERK1 and ERK2 cascade, cellular response to cytokine stimulus, cellular response to interferon-gamma, cellular response to interleukin-1, cellular response to tumor necrosis factor, protein localization to vacuole, negative regulation of substrate adhesion-dependent cell spreading, regulation of protein localization to plasma membrane, negative regulation of protein localization to plasma membrane.
AL591684.2	*pseudogene*	10	2.376277556	0.028254	Unknown function.
PLIN4	perilipin 4	19	2.343282602	0.041066	Peroxisome proliferator-activated receptor (PPAR) signaling pathway.

HLA-DQ B1	major histocompatibility complex, class II, DQ beta 1	6	2.3274923 39	0.0080 40	Adaptive immune response, antigen processing and presentation of exogenous peptide antigen via MHC class I, TAP-independent, peptide antigen assembly with MHC class II protein complex, antigen processing and presentation of peptide or polysaccharide antigen via MHC class II, immune response, humoral immune response, antigen processing and presentation, antigen processing and presentation of exogenous peptide antigen via MHC class II, leukocyte activation, positive regulation of immune response, T cell receptor signaling pathway, positive regulation of T cell activation, interferon-gamma-mediated signaling pathway.
STAT1	signal transducer and activator of transcription 1	2	2.2884864 27	0.0026 75	Pathways activated by bioactive peptides and responses to DNA damage, including apoptotic signaling. Examines the EGF, IFN alpha, IFN gamma, and p38 MAPK signaling pathways, as well as the anti-inflammatory effects of IL-10 and IL22 soluble receptor pathways. Involves the inhibition of cellular proliferation by Gleevec, PDGF, MAPKinase, and TPO signaling pathways.
FCGR2C	Fc fragment of IgG receptor IIc	1	2.2540210 64	0.0005 60	Immune response, cell surface receptor signaling pathway, regulation of immune response.

ITGAX	integrin subunit alpha X	16	2.205134749	0.009114	Cell adhesion, cell-matrix adhesion, integrin-mediated signaling pathway, positive regulation of cell proliferation, animal organ morphogenesis, positive regulation of gene expression, extracellular matrix organization, positive regulation of cell migration, positive regulation of myelination, cell adhesion mediated by integrin, heterotypic cell-cell adhesion, positive regulation of angiogenesis, defense response to virus, cell-cell adhesion, positive regulation of endothelial tube morphogenesis.
NLRC5	NLR family CARD domain containing 5	16	2.196500685	0.003189	Response to bacterium and virus, negative regulation of NFKB, intracellular signal transduction, regulation of kinase activity, innate immune response, positive regulation of MHC class I, positive regulation of transcription from RNA polymerase II promoter, positive regulation of IFN-gamma signaling pathway, positive regulation of type I IFN pathway.
MYO1F	myosin IF	19	2.181796777	0.000892	Actin filament organization, vesicle transport along actin filament.
HLA-DRB5	major histocompatibility complex, class II, DR beta 5	6	2.179719845	0.007087	Adaptive immune response, peptide antigen assembly with MHC class II protein complex, antigen processing and presentation of peptide or polysaccharide antigen via MHC class II, immune response, antigen processing and presentation, antigen processing and presentation of exogenous peptide antigen via MHC class II, positive

					regulation of immune response, positive regulation of T cell activation.
PARVG	parvin gamma	22	2.128315319	0.005487	Cell-matrix adhesion, establishment or maintenance of cell polarity, cell projection assembly, actin cytoskeleton organization, actin cytoskeleton reorganization, substrate adhesion-dependent cell spreading.
AL669918.1	*pseudogene*	6	2.079192925	0.011361	Unknown function.

FCGR2B	Fc fragment of IgG receptor IIb	1	2.06535988	0.020555	Negative regulation of type I hypersensitivity, negative regulation of antibody-dependent cellular cytotoxicity, negative regulation of cytokine production, follicular dendritic cell activation, mature B cell differentiation involved in immune response, follicular B cell differentiation, immune complex clearance by monocytes and macrophages, negative regulation of dendritic cell antigen processing and presentation, regulation of B cell antigen processing and presentation, negative regulation of immunoglobulin production, regulation of adaptive immune response, negative regulation of acute inflammatory response to antigenic stimulus, positive regulation of humoral immune response, negative regulation of humoral immune response mediated by circulating immunoglobulin, receptor-mediated endocytosis, phagocytosis, engulfment, defense response, inflammatory response, immune response, signal transduction, cell surface receptor signaling pathway, response to bacterium, regulation of receptor activity, immunoglobulin mediated immune response, antigen processing and presentation of exogenous peptide antigen via MHC class II, cerebellum development, negative regulation of B cell proliferation, negative regulation of interleukin-10 production, Fc-gamma receptor signaling pathway involved in phagocytosis, negative regulation of macrophage activation, negative regulation of

					cytotoxic T cell degranulation, regulation of innate immune response, positive regulation of JNK cascade, negative regulation of phagocytosis, positive regulation of phagocytosis, regulation of immune response, negative regulation of immune response, negative regulation of B cell receptor signaling pathway, negative regulation of B cell activation, cellular response to molecule of bacterial origin, regulation of immune complex clearance by monocytes and macrophages, positive regulation of neuron death, negative regulation of neutrophil activation, regulation of dendritic spine maintenance, regulation of endoplasmic reticulum stress-induced neuron intrinsic apoptotic signaling pathway, cellular response to beta-amyloid, positive regulation of response to endoplasmic reticulum stress, negative regulation of dendritic cell differentiation.
HLA-DPA1	major histocompatibility complex, class II, DP alpha 1	6	2.059032404	0.001548	Adaptive immune response, peptide antigen assembly with MHC class II protein complex, antigen processing and presentation of peptide or polysaccharide antigen via MHC class II, immune response, antigen processing and presentation, antigen processing and presentation of exogenous peptide antigen via MHC class II, positive regulation of interferon-gamma production, positive regulation of T cell proliferation, positive regulation of immune response, positive regulation of T cell

					activation, cellular response to interferon-gamma.
PIK3CD	phosphatidylinositol-4,5-bisphosphate 3-kinase catalytic subunit delta	1	2.0571081 09	0.0065 31	Natural killer cell differentiation, positive regulation of cytokine production, positive regulation of endothelial cell proliferation, adaptive immune response, mast cell chemotaxis, respiratory burst involved in defense response, protein phosphorylation, inflammatory response, immune response, signal transduction, positive regulation of endothelial cell migration, positive regulation of gene expression, T cell chemotaxis, phosphatidylinositol 3-kinase signaling, phosphorylation, cell migration, natural killer cell activation, B cell differentiation, T cell differentiation, positive regulation of cell migration, neutrophil chemotaxis, positive regulation of neutrophil apoptotic process, natural killer cell chemotaxis, B cell chemotaxis, phosphatidylinositol-3-phosphate biosynthetic process, positive regulation of cell migration by vascular endothelial growth factor signaling pathway, T cell activation, B cell activation, mast cell degranulation, protein kinase B signaling, innate immune response, positive regulation of angiogenesis, phosphatidylinositol phosphorylation, phosphatidylinositol-mediated signaling, T cell receptor signaling pathway, B cell receptor signaling pathway, positive regulation of protein kinase B signaling, mast cell differentiation, neutrophil extravasation, positive regulation of epithelial tube formation.

PLCB2	phospholipase C beta 2	15	2.046271062	0.008499	Detection of chemical stimulus involved in sensory perception of bitter taste, phospholipid metabolic process, G-protein coupled receptor signaling pathway, phospholipase C-activating G-protein coupled receptor signaling pathway, activation of phospholipase C activity, lipid catabolic process, intracellular signal transduction, phosphatidylinositol metabolic process, phosphatidylinositol-mediated signaling, release of sequestered calcium ion into cytosol.

HLA-DRB1	major histocompatibility complex, class II, DR beta 1	6	2.039507465	0.001548	Positive regulation of T cell mediated cytotoxicity, positive regulation of protein phosphorylation, adaptive immune response, inflammatory response to antigenic stimulus, myeloid dendritic cell antigen processing and presentation, antigen processing and presentation of endogenous peptide antigen via MHC class II, peptide antigen assembly with MHC class II protein complex, antigen processing and presentation of peptide or polysaccharide antigen via MHC class II, positive regulation of T cell mediated immune response to tumor cell, negative regulation of inflammatory response to antigenic stimulus, immune response, humoral immune response, cytoskeleton organization, signal transduction, epidermis development, detection of bacterium, antigen processing and presentation, antigen processing and presentation of exogenous peptide antigen via MHC class II, macrophage differentiation, regulation of interleukin-10 production, regulation of interleukin-4 production, negative regulation of interferon-gamma production, positive regulation of CD4-positive, CD25-positive, alpha-beta regulatory T cell differentiation, positive regulation of kinase activity, positive regulation of insulin secretion involved in cellular response to glucose stimulus, T-helper 1 type immune response, negative regulation of T cell proliferation, positive regulation of I-kappaB

					kinase/NF-kappaB signaling, positive regulation of memory T cell differentiation, positive regulation of MAPK cascade, regulation of T-helper cell differentiation, positive regulation of monocyte differentiation, positive regulation of transcription, DNA-templated, positive regulation of viral entry into host cell, positive regulation of immune response, T cell receptor signaling pathway, positive regulation of T cell activation, protein tetramerization, positive regulation of ERK1 and ERK2 cascade, positive regulation of CD4-positive, alpha-beta T cell activation.

HLA-DRA	major histocompatibility complex, class II, DR alpha	6	2.034216569	0.006138	Positive regulation of T cell mediated cytotoxicity, positive regulation of protein phosphorylation, adaptive immune response, inflammatory response to antigenic stimulus, myeloid dendritic cell antigen processing and presentation, antigen processing and presentation of endogenous peptide antigen via MHC class II, peptide antigen assembly with MHC class II protein complex, antigen processing and presentation of peptide or polysaccharide antigen via MHC class II, positive regulation of T cell mediated immune response to tumor cell, negative regulation of inflammatory response to antigenic stimulus, immune response, humoral immune response, cytoskeleton organization, signal transduction, epidermis development, detection of bacterium, antigen processing and presentation, antigen processing and presentation of exogenous peptide antigen via MHC class II, macrophage differentiation, regulation of interleukin-10 production, regulation of interleukin-4 production, negative regulation of interferon-gamma production, positive regulation of CD4-positive, CD25-positive, alpha-beta regulatory T cell differentiation, positive regulation of kinase activity, positive regulation of insulin secretion involved in cellular response to glucose stimulus, T-helper 1 type immune response, negative regulation of T cell proliferation, positive regulation of I-kappaB

					kinase/NF-kappaB signaling, positive regulation of memory T cell differentiation, positive regulation of MAPK cascade, regulation of T-helper cell differentiation, positive regulation of monocyte differentiation, positive regulation of transcription, DNA-templated, positive regulation of viral entry into host cell, positive regulation of immune response, T cell receptor signaling pathway, positive regulation of T cell activation, protein tetramerization, positive regulation of ERK1 and ERK2 cascade, positive regulation of CD4-positive, alpha-beta T cell activation.
AC009533.1	*pseudogene*	12	2.008346788	0.011962	Unknown function.
IL10RA	interleukin 10 receptor subunit alpha	11	2.008001799	0.001385	Negative regulation of autophagy, cytokine-mediated signaling pathway, response to lipopolysaccharide, positive regulation of JAK-STAT cascade, negative regulation of inflammatory response, regulation of synapse organization, intestinal epithelial structure maintenance, ubiquitin-dependent endocytosis.
ACAP1	ArfGAP with coiled-coil, ankyrin repeat and PH domains 1	17	2.000364022	0.007133	Protein transport.

HLA-DPB1	major histocompatibility complex, class II, DP beta 1	16	1.998446437	0.007087	Adaptive immune response, antigen processing and presentation of exogenous peptide antigen via MHC class I, TAP-independent, peptide antigen assembly with MHC class II protein complex, antigen processing and presentation of peptide or polysaccharide antigen via MHC class II, immune response, antigen processing and presentation, antigen processing and presentation of exogenous peptide antigen via MHC class II, positive regulation of interferon-gamma production, positive regulation of T cell proliferation, leukocyte activation, positive regulation of immune response, T cell receptor signaling pathway, positive regulation of T cell activation, interferon-gamma-mediated signaling pathway.
CLEC10A	C-type lectin domain containing 10A	17	1.989306309	0.007209	Adaptive immune response, endocytosis, interspecies interaction between organisms, innate immune response.

ITGB2	integrin subunit beta 2	21	1.948911037	0.016161	Microglial cell activation, receptor-mediated endocytosis, phagocytosis, phagocytosis, engulfment, apoptotic process, inflammatory response, cell adhesion, leukocyte cell-cell adhesion, cell-matrix adhesion, integrin-mediated signaling pathway, cell-cell signaling, regulation of cell shape, cell migration, extracellular matrix organization, neutrophil chemotaxis, receptor internalization, positive regulation of superoxide anion generation, cell adhesion mediated by integrin, heterotypic cell-cell adhesion, endodermal cell differentiation, receptor clustering, positive regulation of neutrophil degranulation, negative regulation of dopamine metabolic process, regulation of peptidyl-tyrosine phosphorylation, cellular response to low-density lipoprotein particle stimulus, positive regulation of protein targeting to membrane, beta-amyloid clearance, cell-cell adhesion, cell-cell adhesion via plasma-membrane adhesion molecules, positive regulation of neuron death, positive regulation of leukocyte adhesion to vascular endothelial cell, neutrophil migration, positive regulation of prostaglandin-E synthase activity.

CD74	CD74 molecule	5	1.921113152	0.003189	Prostaglandin biosynthetic process, positive regulation of protein phosphorylation, positive regulation of cytokine-mediated signaling pathway, T cell activation involved in immune response, positive regulation of dendritic cell antigen processing and presentation, negative regulation of peptide secretion, positive regulation of type 2 immune response, negative regulation of mature B cell apoptotic process, intracellular protein transport, immune response, cell proliferation, positive regulation of gene expression, immunoglobulin mediated immune response, antigen processing and presentation, antigen processing and presentation of endogenous antigen, antigen processing and presentation of exogenous peptide antigen via MHC class II, negative regulation of cell migration, positive regulation of B cell proliferation, positive regulation of prostaglandin biosynthetic process, positive regulation of chemokine production, positive regulation of interleukin-6 production, positive regulation of interleukin-8 production, positive regulation of kinase activity, response to interferon-gamma, macrophage migration inhibitory factor signaling pathway, regulation of macrophage activation, negative regulation of apoptotic process, positive regulation of I-kappaB kinase/NF-kappaB signaling, positive regulation of MAPK cascade, negative regulation of DNA damage response, signal

					transduction by p53 class mediator, T cell selection, positive thymic T cell selection, negative thymic T cell selection, negative regulation of T cell differentiation, positive regulation of T cell differentiation, positive regulation of monocyte differentiation, positive regulation of transcription, DNA-templated, negative regulation of viral entry into host cell, positive regulation of viral entry into host cell, positive regulation of fibroblast proliferation, positive regulation of peptidyl-tyrosine phosphorylation, protein stabilization, chaperone mediated protein folding requiring cofactor, positive regulation of macrophage cytokine production, macromolecular complex assembly, protein trimerization, positive regulation of ERK1 and ERK2 cascade, positive regulation of neutrophil chemotaxis, negative regulation of intrinsic apoptotic signaling pathway in response to DNA damage by p53 class mediator, positive regulation of chemokine (C-X-C motif) ligand 2 production, positive regulation of macrophage migration inhibitory factor signaling pathway.
LAP3	leucine aminopeptidase 3	4	1.91484213	0.001385	Proteolysis.

GBP2	guanylate binding protein 2	1	1.906764964	0.007277	Activation of innate immune response, immune response, protein localization to nucleus, defense response to bacterium, defense response to protozoan, defense response to Gram-positive bacterium, defense response to virus, cytolysis in other organism, cellular response to lipopolysaccharide, cellular response to cytokine stimulus, cellular response to interferon-gamma, cellular response to interleukin-1, cellular response to tumor necrosis factor.
FERMT3	fermitin family member 3	11	1.90582668	0.010879	Leukocyte cell-cell adhesion, cell-matrix adhesion, integrin-mediated signaling pathway, positive regulation of cell migration, integrin activation, regulation of cell-cell adhesion mediated by integrin, substrate adhesion-dependent cell spreading, platelet aggregation.

SULF1	sulfatase 1	8	1.877736936	0.023986	Kidney development, negative regulation of endothelial cell proliferation, chondrocyte development, glomerular filtration, apoptotic process, positive regulation of vascular endothelial growth factor production, esophagus smooth muscle contraction, negative regulation of angiogenesis, positive regulation of Wnt signaling pathway, heparan sulfate proteoglycan metabolic process, negative regulation of cell migration, positive regulation of BMP signaling pathway, glomerular basement membrane development, glial cell-derived neurotrophic factor receptor signaling pathway, regulation of fibroblast growth factor receptor signaling pathway, negative regulation of fibroblast growth factor receptor signaling pathway, vascular endothelial growth factor receptor signaling pathway, embryonic skeletal system development, cartilage development, bone development, innervation, negative regulation of prostatic bud formation.
IFI27	interferon alpha inducible protein 27 like 2	14	1.866823142	0.004679	Apoptotic process, proteasome-mediated ubiquitin-dependent protein catabolic process, modulation by host of viral genome replication, innate immune response, regulation of protein export from nucleus, defense response to virus, type I IFN signaling pathway, pyroptosis, protein K48-linked ubiquitination, apoptotic signaling pathway, extrinsic apoptotic signaling pathway.

DOCK2	dedicator of cytokinesis 2	5	1.853292547	0.005926	Membrane raft polarization, establishment of T cell polarity, immunological synapse formation, myeloid dendritic cell activation involved in immune response, chemotaxis, small GTPase mediated signal transduction, myoblast fusion, cell migration, actin cytoskeleton organization, macropinocytosis, positive thymic T cell selection, negative thymic T cell selection, alpha-beta T cell proliferation, positive regulation of phagocytosis, regulation of small GTPase mediated signal transduction.
CIITA	class II major histocompatibility complex transactivator	16	1.843412705	0.009429	Negative regulation of transcription from RNA polymerase II promoter, regulation of transcription, DNA-templated, inflammatory response, immune response, aging, phosphorylation, negative regulation of collagen biosynthetic process, response to interferon-gamma, positive regulation of MHC class I biosynthetic process, positive regulation of MHC class II biosynthetic process, negative regulation of transcription, DNA-templated, positive regulation of transcription, DNA-templated, positive regulation of transcription from RNA polymerase II promoter, negative regulation of viral entry into host cell, response to antibiotic, cellular response to electrical stimulus, cellular response to interferon-gamma, cellular response to exogenous dsRNA.

PIK3R5	Phosphoinositide-3-kinase regulatory subunit 5	17	1.838486942	0.002474	Immune response, G-protein coupled receptor signaling pathway, phosphatidylinositol 3-kinase signaling, positive regulation of MAP kinase activity, protein kinase B signaling, positive regulation of protein kinase B signaling.
STK10	serine/threonine kinase 10	5	1.828132963	0.004832	Protein phosphorylation, cell cycle, protein autophosphorylation, lymphocyte aggregation, regulation of lymphocyte migration.
MYO1G	myosin IG	7	1.826531956	0.005367	T cell mediated immunity, exocytosis, actin filament organization, vesicle transport along actin filament, cell-substrate adhesion, Fc-gamma receptor signaling pathway involved in phagocytosis, cell gliding, T cell migration.
LCP2	retinoid X receptor alpha	5	1.805800235	0.004679	Immune response, transmembrane receptor protein tyrosine kinase signaling pathway, intracellular signal transduction, mast cell activation, positive regulation of protein kinase activity, T cell receptor signaling pathway.

TNC	tenascin C	9	1.788161098	0.008961	Regulation of cell growth, osteoblast differentiation, morphogenesis of an epithelium, cell adhesion, negative regulation of cell adhesion, neuromuscular junction development, positive regulation of cell proliferation, response to wounding, response to mechanical stimulus, positive regulation of gene expression, peripheral nervous system axon regeneration, regulation of cell adhesion, regulation of cell migration, regulation of cell proliferation, odontogenesis of dentin-containing tooth, response to ethanol, regulation of inflammatory response, bud outgrowth involved in lung branching, mesenchymal-epithelial cell signaling involved in prostate gland development, prostate gland epithelium morphogenesis, cellular response to retinoic acid, cellular response to vitamin D, response to fibroblast growth factor, cellular response to prostaglandin D stimulus.
TLR2	toll like receptor 2	4	1.773515069	0.007890	Hypoxia response, microglial activation, cytokine regulation, and toll-like receptor signaling. Explores inflammatory and immune responses, apoptosis, signal transduction, and responses to bacterial stimuli. Involves the regulation of gene expression, chemokine production, and cellular responses to various environmental factors.

TRPV2	transient receptor potential cation channel subfamily V member 2	17	1.734442555	0.004554	Sensory perception, response to temperature stimulus, response to heat, positive regulation of axon extension, calcium ion transmembrane transport, positive regulation of calcium ion import, calcium ion import across plasma membrane.
LILRB3	leukocyte immunoglobulin like receptor B3	19	1.725649129	0.007277	Adaptive immune response, defense response, cell surface receptor signaling pathway, cytokine-mediated signaling pathway, negative regulation of osteoclast differentiation.
IRF1	interferon regulatory factor 1	5	1.713285737	0.013261	Immune processes, including adaptive and innate immune responses, and the regulation of transcription related to RNA polymerase II. Includes apoptotic processes, cell proliferation, type I interferon production, and T cell differentiation and proliferation. Key pathways include toll-like receptor signaling, interferon-mediated responses, and cellular reactions to viral defense and mechanical stimuli.
RASSF4	Ras association domain family member 4	10	1.704422168	0.024085	Cell cycle and signal transduction.

HCLS1	hematopoietic cell-specific Lyn substrate 1	3	1.702105373	0.010879	Negative regulation of transcription from RNA polymerase II promoter, regulation of transcription, DNA-templated, actin filament organization, signal transduction, positive regulation of cell proliferation, response to hormone, positive regulation of phosphatidylinositol 3-kinase signaling, actin filament polymerization, erythrocyte differentiation, regulation of actin filament polymerization, positive regulation of granulocyte differentiation, positive regulation of peptidyl-serine phosphorylation, intracellular signal transduction, positive regulation of protein import into nucleus, positive regulation of tyrosine phosphorylation of STAT protein, positive regulation of macrophage differentiation, positive regulation of transcription from RNA polymerase II promoter, positive regulation of peptidyl-tyrosine phosphorylation, positive regulation of sequence-specific DNA binding transcription factor activity, positive regulation of protein kinase B signaling, cellular response to cytokine stimulus, negative regulation of leukocyte apoptotic process, positive regulation of actin cytoskeleton reorganization
FMNL1	formin like 1	17	1.69200873	0.008499	Cytoskeleton organization, regulation of cell shape, cell migration, cortical actin cytoskeleton organization, actin filament severing.

WDFY4	WDFY family member 4	10	1.684183517	0.007890	Autophagy, antigen processing and presentation, CD8-positive, alpha-beta T cell activation, cellular response to virus.
ADA2	adenosine deaminase 2	22	1.677939748	0.015729	Adenosine catabolic process, signal transduction, inosine biosynthetic process.
HIVEP3	human immunodeficiency virus type I enhancer binding protein 3	1	1.650363561	0.008499	Regulation of transcription from RNA polymerase II promoter, skeletal muscle cell differentiation, positive regulation of transcription, DNA-templated.
MTHFD1L	methylenetetrahydrofolate dehydrogenase (NADP+ dependent) 1 like	6	1.644956729	0.007122	Neural tube closure, one-carbon metabolic process, folic acid-containing compound metabolic process, 10-formyltetrahydrofolate biosynthetic process, formate metabolic process, tetrahydrofolate interconversion, embryonic neurocranium morphogenesis, embryonic viscerocranium morphogenesis.

Table S6. Top 50 Upregulated Genes in Inflammatory LS *versus* HC using overlap. *P-values were corrected by multiple hypothesis testing to give the Q value by the Benjamini-Hochberg method. **Functional Annotation is adapted from Database for Annotation, Visualization and Integrated Discovery (DAVID). Chr; chromosome.

Gene	Protein Encoded	Chr.	LogFC	Q value*	**Functional Annotation
AMY2A	amylase, alpha 2A (pancreatic)	1	-4.170188235	0.039265	Carbohydrate metabolic process, carbohydrate catabolic process, polysaccharide digestion.
AMY1B	amylase, alpha 1B (salivary)	1	-3.678760001	0.028565	Carbohydrate metabolic process, oligosaccharide metabolic process.
KRT8P3	keratin 8 pseudogene 33	5	-3.560451607	0.040205	Unknown function.
UBBP4	ubiquitin B pseudogene 4	17	-3.275834182	0.047363	Unknown function.
TSPAN8	tetraspanin 8	12	-3.25062302	0.011921	Spermatogenesis, regulation of gene expression, negative regulation of blood coagulation.
FP671120.3	*pseudogene*	21	-3.246161734	0.018770	Unknown function.
FP236383.3	*pseudogene*		-3.195157557	0.018770	Unknown function.

OSR2	odd-skipped related transciption factor 2	8	-3.06060553	0.000540	Negative regulation of transcription from RNA polymerase II promoter, urogenital system development, metanephros development, mesonephros development, chondrocyte differentiation, regulation of transcription from RNA polymerase II promoter, positive regulation of cell proliferation, embryo development, embryo development ending in birth or egg hatching, positive regulation of gene expression, cell differentiation, positive regulation of bone mineralization, osteoblast proliferation, embryonic forelimb morphogenesis, embryonic hindlimb morphogenesis, embryonic skeletal limb joint morphogenesis, middle ear morphogenesis, odontogenesis, embryonic digit morphogenesis, positive regulation of transcription, DNA-templated, positive regulation of transcription from RNA polymerase II promoter, embryonic skeletal system morphogenesis, positive regulation of epithelial cell proliferation, palate development, embryonic skeletal joint morphogenesis, head development, bone morphogenesis, eyelid development in camera-type eye, stem cell proliferation, embryonic skeletal joint development, positive

					regulation of stem cell proliferation.
CD24	CD24 molecule	6	-2.615741189	0.007277	Response to hypoxia, cell activation, regulation of cytokine-mediated signaling pathway, response to molecule of bacterial origin, immune response-regulating cell surface receptor signaling pathway, cell adhesion, positive regulation of cytosolic calcium ion concentration, Wnt signaling pathway, cell migration, regulation of epithelial cell differentiation, T cell costimulation, B cell receptor transport into membrane raft, chemokine receptor transport out of membrane raft, negative regulation of transforming growth factor beta3 production, positive regulation of activated T cell proliferation, regulation of phosphorylation, cholesterol homeostasis, positive regulation of MAP kinase activity, regulation of MAPK cascade, response to estrogen, respiratory burst, glomerular visceral epithelial cell differentiation, glomerular parietal epithelial cell differentiation, intrinsic apoptotic signaling pathway, cell-cell adhesion, positive regulation of nephron tubule epithelial cell differentiation.
AC133435.1	*pseudogene*	3	-2.530711802	0.040446	Unknown function.

LGR5	leucine rich repeat containing G protein-coupled receptor 5	12	-2.518130271	0.008499	Hair follicle development, G-protein coupled receptor signaling pathway, adenylate cyclase-activating G-protein coupled receptor signaling pathway, activation of adenylate cyclase activity, hormone-mediated signaling pathway, oocyte differentiation, regulation of cell proliferation, inner ear development, positive regulation of canonical Wnt signaling pathway, epithelial cell proliferation involved in renal tubule morphogenesis.
ADTRP	androgen dependent TFPI regulating protein	6	-2.509579769	0.003189	Positive regulation of protein phosphorylation, cell migration involved in sprouting angiogenesis, negative regulation of leukocyte migration, negative regulation of extracellular matrix constituent secretion, positive regulation of gene expression, negative regulation of blood coagulation, long-chain fatty acid catabolic process, protein kinase B signaling, negative regulation of protein secretion, cellular response to steroid hormone stimulus, negative regulation of leukocyte cell-cell adhesion, negative regulation of lymphocyte migration.
OGN	osteoglycin	9	-2.472202637	0.015354	Signal transduction, negative regulation of smooth muscle cell proliferation, bone development.

CPS1	carbamoyl-phosphate synthase 1	2	-2.464567147	0.035175	Urea cycle, 'de novo' pyrimidine nucleobase biosynthetic process, glutamine metabolic process, nitrogen compound metabolic process, midgut development, response to xenobiotic stimulus, response to toxic substance, response to zinc ion, response to amine, citrulline biosynthetic process, triglyceride catabolic process, response to food, response to lipopolysaccharide, cellular nitrogen compound metabolic process, vasodilation, response to starvation, response to amino acid, cellular response to fibroblast growth factor stimulus, nitric oxide metabolic process, homocysteine metabolic process, anion homeostasis, response to growth hormone, hepatocyte differentiation, carbamoyl phosphate biosynthetic process, cellular response to ammonium ion, cellular response to cAMP, cellular response to glucagon stimulus, cellular response to oleic acid, response to dexamethasone.
SCARA5	scavenger receptor class A member 5	8	-2.437200597	0.012122	Cellular iron ion homeostasis, endocytosis, cellular response to heat, iron ion transmembrane transport, protein homo-trimerization.
MGST1	microsomal glutathione S-transferase 1	12	-2.43441883	0.031058	Glutathione transport, cellular response to lipid hydroperoxide, cellular oxidant detoxification.

CADPS	calcium dependent secretion activator	3	-2.387944656	0.005367	Exocytosis, protein transport, synaptic vesicle exocytosis, positive regulation of exocytosis, dense core granule exocytosis.
LYVE1	lymphatic vessel endothelial hyaluronan receptor 1	11	-2.383506163	0.028254	Positive regulation of cellular extravasation, glycosaminoglycan catabolic process, receptor-mediated endocytosis, cell adhesion, cell-matrix adhesion, response to wounding, anatomical structure morphogenesis, hyaluronan catabolic process.
MTATP6P1	mitochondrially encoded ATP synthase 6 pseudogene 1	1	-2.32676152	0.031058	Unknown function.
SNORD3B-2	small nucleolar RNA, C/D box 3B-2	17	-2.277587514	0.003845	Ribosome biogenesis in eukaryotes.
CAMK2N1	calcium/calmodulin dependent protein kinase II inhibitor 1	1	-2.277548327	0.002911	Negative regulation of protein kinase activity, long-term memory, positive regulation of inflammatory response.

PKP2	plakophilin 2	12	-2.266692891	0.007122	Desmosome assembly, desmosome organization, cell-cell junction assembly, heart development, positive regulation of sodium ion transport, regulation of cell-substrate adhesion, intermediate filament bundle assembly, maintenance of animal organ identity, ventricular cardiac muscle tissue morphogenesis, protein localization to plasma membrane, cardiac muscle cell action potential involved in contraction, ventricular cardiac muscle cell action potential, cell-cell signaling involved in cardiac conduction, cell communication by electrical coupling involved in cardiac conduction, bundle of His cell-Purkinje myocyte adhesion involved in cell communication, regulation of heart rate by cardiac conduction, cell-cell adhesion, regulation of ventricular cardiac muscle cell action potential, regulation of substrate adhesion-dependent cell spreading.
ASS1P11	argininosuccinate synthetase 1 pseudogene 11	7	-2.204292648	0.020836	Unknown function.
MSMO1	methylsterol monooxygenase 1	4	-2.20034369	0.008499	Fatty acid metabolic process, cholesterol biosynthetic process, steroid metabolic process, lipid biosynthetic process, sterol biosynthetic process.

CKMT1A	creatine kinase, mitochondrial 1A	15	-2.182128683	0.016096	Phosphorylation, carboxylic acid metabolic process, phosphocreatine biosynthetic process.
TFF3	trefoil factor 3	21	-2.156749162	0.031727	Regulation of glucose metabolic process, maintenance of gastrointestinal epithelium.
SEMA3B	semaphorin 3B	3	-2.129579004	0.003190	Neural crest cell migration, cell-cell signaling, axon guidance, positive regulation of cell migration, negative regulation of axon extension involved in axon guidance, negative chemotaxis, chemorepulsion of axon, semaphorin-plexin signaling pathway.
SATB2	SATB homeobox 2	2	-2.106182611	0.013358	Negative regulation of transcription from RNA polymerase II promoter, neuron migration, osteoblast development, chromatin remodeling, regulation of transcription from RNA polymerase II promoter, embryonic pattern specification, commitment of neuronal cell to specific neuron type in forebrain, positive regulation of transcription from RNA polymerase II promoter, embryonic skeletal system morphogenesis, cartilage development, palate development, cellular response to organic substance.

CREB3L1	cAMP responsive element binding protein 3 like 1	11	-2.091768877	0.001876	Negative regulation of transcription from RNA polymerase II promoter, osteoblast differentiation, regulation of transcription from RNA polymerase II promoter, negative regulation of gene expression, endoplasmic reticulum unfolded protein response, positive regulation of collagen biosynthetic process, negative regulation of fibroblast growth factor receptor signaling pathway, negative regulation of transcription, DNA-templated, positive regulation of transcription from RNA polymerase II promoter, extracellular matrix constituent secretion, negative regulation of endoplasmic reticulum stress-induced intrinsic apoptotic signaling pathway, negative regulation of sprouting angiogenesis, positive regulation of transcription from RNA polymerase II promoter in response to endoplasmic reticulum stress.
RN7SL1	RNA, 7SL, cytoplasmic 1	14	-2.089545067	0.013015	SRP-dependent cotranslational protein targeting to membrane, signal sequence recognition.
AL139099.4	*pseudogene*	14	-2.050213597	0.013262	Unknown function.

FBLN1	fibulin 1	22	-1.907581315	0.033096	Negative regulation of protein phosphorylation, cell adhesion, and ERK1/ERK2 signaling cascades, as well as the suppression of transforming growth factor beta production, cell motility, stem cell proliferation, and virus-induced host cell transformation. Additionally, it emphasizes positive regulation of gene expression, fibroblast proliferation, and substrate-dependent cell migration. Key processes also include embryo implantation, extracellular matrix organization, blood coagulation, fibrin clot formation, and cell attachment to substrates.
ATP5MC3	ATP synthase membrane subunit c locus 3	2	-1.862944275	0.003107	ATP synthesis coupled proton transport, cellular component organization, hydrogen ion transmembrane transport.
GREM1	gremlin 1, DAN family BMP antagonist	15	-1.860441855	0.028814	Cell morphogenesis, sprouting angiogenesis, transcription regulation in heart and kidney development, signal transduction, cell proliferation, limb and organ morphogenesis, bone and cartilage growth regulation, angiogenesis, DNA transcription, and cell differentiation. It also involves various negative regulations in bone remodeling and Wnt signaling, and positive influences on NF-kappaB activity, telomerase activity,

					and cardiac muscle development.
H2AFJ	H2A histone family member J	12	-1.854464785	0.015632	Unknown function.
PANK1	pantothenate kinase 1	10	-1.847816541	0.004679	Coenzyme A biosynthetic process, phosphorylation.
PCSK5	proprotein convertase subtilisin/kexin type 5	9	-1.826550795	0.003832	Kidney development, renin secretion into blood stream, cardiac septum development, signal peptide processing, cell-cell signaling, determination of left/right symmetry, heart development, embryo implantation, anterior/posterior pattern specification, protein processing, peptide hormone processing, viral life cycle, respiratory tube development, plasma lipoprotein particle remodeling, limb morphogenesis, peptide biosynthetic process, embryonic digestive tract development, embryonic skeletal system development, regulation of lipoprotein lipase activity, coronary vasculature development.

SEMA6D	semaphorin 6D	15	-1.826086605	0.009363	Neural crest cell migration, nervous system development, axon guidance, smooth muscle cell migration, positive regulation of smooth muscle cell migration, negative regulation of smooth muscle cell migration, ventricular system development, cell differentiation, positive regulation of cell migration, negative regulation of axon extension, negative regulation of axon extension involved in axon guidance, negative chemotaxis, semaphorin-plexin signaling pathway.

NR5A2	nuclear receptor subfamily 5 group A member 2	1	-1.786722497	0.035392	Regulation of transcription, DNA-templated, regulation of transcription from RNA polymerase II promoter, bile acid metabolic process, hormone-mediated signaling pathway, embryo development ending in birth or egg hatching, tissue development, intracellular receptor signaling pathway, epithelial cell differentiation, regulation of cell proliferation, homeostatic process, cholesterol homeostasis, positive regulation of viral genome replication, positive regulation of transcription, DNA-templated, positive regulation of transcription from RNA polymerase II promoter, pancreas morphogenesis, acinar cell differentiation, calcineurin-mediated signaling, cellular response to leukemia inhibitory factor.
HEPH	hephaestin	X	-1.78415287	0.027307	Copper ion transport, iron ion transport, cellular iron ion homeostasis, iron ion transmembrane transport, iron ion homeostasis.

DSG2	desmoglein 2	18	-1.780408402	0.037604	Desmosome organization, Purkinje myocyte development, cell adhesion, homophilic cell adhesion via plasma membrane adhesion molecules, response to progesterone, maternal process involved in female pregnancy, bundle of His cell-Purkinje myocyte adhesion involved in cell communication, regulation of heart rate by cardiac conduction, cell-cell adhesion, regulation of ventricular cardiac muscle cell action potential.
AFF2	AF4/FMR2 family member 2	X	-1.777646634	0.017870	mRNA processing, brain development, learning or memory, RNA splicing, regulation of gene expression, negative regulation of gene expression, nuclear speck organization, regulation of RNA splicing.
FMO5	flavin containing monooxygenase 5	1	-1.746896323	0.013058	Lipid metabolic process, xenobiotic metabolic process, NADPH oxidation, regulation of cholesterol metabolic process.
PPIC	peptidylprolyl isomerase C	5	-1.744440974	0.003189	Protein peptidyl-prolyl isomerization, protein folding.
TCP1P1	t-complex 1 pseudogene 1	7	-1.738508122	0.017983	Unknown function.

GPC4	glypican 4	X	-1.73826001	0.033568	Retinoid metabolic process, glycosaminoglycan biosynthetic process, glycosaminoglycan catabolic process, regulation of signal transduction, Wnt signaling pathway, cell migration, synaptic membrane adhesion, regulation of protein localization to membrane, regulation of presynapse assembly.
IGSF10	immunoglobulin superfamily member 10	3	-1.736311016	0.035446	Ossification, cell differentiation, regulation of neuron migration.
HMGCS1	3-hydroxy-3-methylglutaryl-CoA synthase 1	5	-1.733035271	0.000893	Acetyl-CoA metabolic process, lipid metabolic process, cholesterol biosynthetic process, cholesterol metabolic process, farnesyl diphosphate biosynthetic process, mevalonate pathway, sterol biosynthetic process.
GREB1L	GREB1 like retinoic acid receptor coactivator	18	-1.73160728	0.019286	Metanephros development, branching involved in ureteric bud morphogenesis, kidney development, cardiac ventricle development, multicellular organism development, male genitalia development, uterus development, paramesonephric duct development, mesonephric duct development.
AC104651.1	*pseudogene*	2	-1.721831279	0.031727	Unknown function.

Table S7. Top 50 Downregulated Genes in Inflammatory LS *versus* HC using overlap. *P-values were corrected by multiple hypothesis testing to give the Q value by the Benjamini-Hochberg method. **Functional Annotation is adapted from Database for Annotation, Visualization and Integrated Discovery (DAVID). Chr; chromosome.

Gene	Protein Encoded	Ch r.	LogFC	Q value*	**Functional Annotation
KRT1	keratin 1	12	4.180481563	0.004305	Complement activation, lectin pathway, retina homeostasis, response to oxidative stress, peptide cross-linking, keratinization, fibrinolysis, intermediate filament organization, regulation of angiogenesis, negative regulation of inflammatory response, protein heterotetramerization, establishment of skin barrier, cornification.
FLG	filaggrin	1	4.134427223	0.002592	Peptide cross-linking, keratinocyte differentiation, establishment of skin barrier.
KRT5	keratin 5	12	3.714483672	0.005620	Epidermis development, response to mechanical stimulus, regulation of cell migration, keratinization, regulation of protein localization, intermediate filament polymerization, intermediate filament organization, cornification
KRT10	keratin 10	17	3.697007159	0.006626	Epidermis development, peptide cross-linking, keratinocyte differentiation, epithelial cell differentiation, intermediate filament organization, positive regulation of epidermis development, protein heterotetramerization.
PKP1	plakophilin 1	1	3.196047565	0.002063	Cell-cell junction assembly, cell adhesion, signal transduction, positive regulation of gene expression, intermediate filament bundle assembly, cell-cell adhesion, negative regulation of mRNA catabolic process.

KRT14	keratin 14	17	3.178217983	0.002761	Aging, epidermis development, keratinocyte differentiation, epithelial cell differentiation, hair cycle, intermediate filament organization, intermediate filament bundle assembly, stem cell differentiation.
CDHR1	cadherin related family member 1	10	2.971847374	0.002326	Cell adhesion.
SPTBN2	spectrin beta, non-erythrocytic 2	11	2.763828763	0.001470	Cell communication, synapse assembly, vesicle-mediated transport, cerebellar Purkinje cell layer morphogenesis, signaling, actin cytoskeleton organization, adult behavior, multicellular organism growth, response to stimulus, cellular localization, actin filament capping.
KRT6A	keratin 6A	12	2.753625558	0.032472	Negative regulation of cytolysis by symbiont of host cells, morphogenesis of an epithelium, positive regulation of cell proliferation, cell differentiation, keratinization, killing of cells of other organism, wound healing, intermediate filament organization, defense response to Gram-positive bacterium, cytolysis by host of symbiont cells, antimicrobial humoral immune response mediated by antimicrobial peptide, cornification, negative regulation of entry of bacterium into host cell.
CLCA2	chloride channel accessory 2	1	2.617451236	0.006132	Proteolysis, chloride transport, cell adhesion, ion transmembrane transport, chloride transmembrane transport.
COL17A1	collagen type XVII alpha 1 chain	10	2.564707485	0.001123	Cell-matrix adhesion, epidermis development, extracellular matrix organization, hemidesmosome assembly.

FO538757.1	*pseudogene*	1	2.550255793	0.009472	Unknown function.
GOLGA6L10	golgin A6 family-like 10	15	2.537099641	0.017859	Unknown function.
FAT2	FAT atypical cadherin 2	5	2.533131085	0.001449	Homophilic cell adhesion via plasma membrane adhesion molecules, epithelial cell migration, cell-substrate adhesion, cell-cell adhesion.
NEB	nebulin	2	2.510090271	0.028165	Muscle organ development, somatic muscle development, regulation of actin filament length, cardiac muscle thin filament assembly.
COL7A1	collagen type VII alpha 1 chain	3	2.39272439	0.000904	Cell adhesion, epidermis development, extracellular matrix organization, endodermal cell differentiation.
PHGDH	phosphoglycerate dehydrogenase	1	2.367160009	0.005228	Glutamine metabolic process, glycine metabolic process, L-serine biosynthetic process, threonine metabolic process, brain development, gamma-aminobutyric acid metabolic process, regulation of gene expression, taurine metabolic process, spinal cord development, glial cell development, neural tube development, electron transport chain, neuron projection development, G1 to G0 transition.
DSC3	desmocollin 3	18	2.341681257	0.008871	In utero embryonic development, cell adhesion, homophilic cell adhesion via plasma membrane adhesion molecules, system development, protein stabilization, cell-cell adhesion.
HR	HR lysine demethylase and nuclear receptor corepressor	8	2.331994081	0.001297	Chromatin remodeling, regulation of transcription from RNA polymerase II promoter,histone H3-K9 demethylation.

RARG	retinoic acid receptor gamma	12	2.279375556	0.002229	Negative regulation of transcription from RNA polymerase II promoter, neural tube closure, glandular epithelial cell development, growth plate cartilage chondrocyte growth, apoptotic process, positive regulation of cell proliferation, negative regulation of cell proliferation, regulation of cell size, hormone-mediated signaling pathway, anterior/posterior pattern specification, positive regulation of gene expression, cell differentiation, embryonic camera-type eye development, regulation of myelination, negative regulation of chondrocyte differentiation, response to retinoic acid, embryonic hindlimb morphogenesis, multicellular organism growth, positive regulation of apoptotic process, positive regulation of programmed cell death, regulation of myeloid cell differentiation, positive regulation of transcription from RNA polymerase II promoter, embryonic eye morphogenesis, retinoic acid receptor signaling pathway, canonical Wnt signaling pathway, face development, trachea cartilage development, prostate gland epithelium morphogenesis, Harderian gland development, cellular response to retinoic acid, stem cell proliferation, cellular response to leukemia inhibitory factor, negative regulation of stem cell proliferation.
LINC01001	long intergenic non-protein coding RNA 1001	11	2.274660512	0.003033	Unknown function.

DSP	desmoplakin	6	2.207740769	0.001732	Desmosome organization, ventricular compact myocardium morphogenesis, epidermis development, peptide cross-linking, keratinocyte differentiation, adherens junction organization, wound healing, skin development, intermediate filament cytoskeleton organization, intermediate filament organization, bundle of His cell-Purkinje myocyte adhesion involved in cell communication, regulation of heart rate by cardiac conduction, epithelial cell-cell adhesion, cell-cell adhesion, regulation of ventricular cardiac muscle cell action potential.
CELSR2	cadherin EGF LAG seven-pass G-type receptor 2	1	2.20505622	0.000904	Neuron migration, G-protein coupled receptor signaling pathway, Wnt signaling pathway, regulation of cell-cell adhesion, regulation of protein localization, dendrite morphogenesis, planar cell polarity pathway, cilium assembly, motor neuron migration, cell-cell adhesion.
EPHB6	EPH receptor B6	7	2.169451165	0.001028	Type IV hypersensitivity, protein phosphorylation, transmembrane receptor protein tyrosine kinase signaling pathway, axon guidance, central nervous system projection neuron axonogenesis, positive regulation of protein binding, positive regulation of kinase activity, ephrin receptor signaling pathway, activated T cell proliferation, positive regulation of T cell costimulation.
PDZD2	PDZ domain containing 2	5	2.155099231	0.000610	Cell adhesion.
AHNAK2	AHNAK nucleoprotein 2	14	2.148132177	0.005180	Regulation of RNA splicing.

EPS8L1	EPS8 like 1	19	2.1435018 87	0.00070 1	Rho protein signal transduction, regulation of Rho protein signal transduction, regulation of catalytic activity, positive regulation of ruffle assembly.
PLCH2	phospholipase C eta 2	1	2.1220169	0.00055 7	Lipid catabolic process, intracellular signal transduction, inositol phosphate metabolic process, phosphatidylinositol metabolic process, phosphatidylinositol-mediated signaling, release of sequestered calcium ion into cytosol.
ANO1	anoctamin 1	11	2.0699477 65	0.00149 1	Cation transport, chloride transport, phospholipase C-activating G-protein coupled receptor signaling pathway, iodide transport, ion transmembrane transport, cellular response to heat, positive regulation of insulin secretion involved in cellular response to glucose stimulus, detection of temperature stimulus involved in sensory perception of pain, transmembrane transport, mucus secretion, protein localization to membrane, cation transmembrane transport, cellular response to peptide, chloride transmembrane transport.
EVPL	envoplakin	17	2.0483710 68	0.00046 5	Regulation of antibacterial peptide production, epidermis development, peptide cross-linking, keratinocyte differentiation, keratinization, wound healing, intermediate filament cytoskeleton organization.

EPHA4	EPH receptor A4	2	2.039995428	0.001779	Protein phosphorylation, cell adhesion, negative regulation of cell adhesion, transmembrane receptor protein tyrosine kinase signaling pathway, axon guidance, adult walking behavior, motor neuron axon guidance, positive regulation of cell proliferation, glial cell migration, negative regulation of epithelial to mesenchymal transition, negative regulation of neuron projection development, negative regulation of translation, peptidyl-tyrosine phosphorylation, corticospinal tract morphogenesis, positive regulation of cell migration, negative regulation of cell migration, positive regulation of kinase activity, adherens junction organization, regulation of GTPase activity, positive regulation of JUN kinase activity, positive regulation of cell adhesion, protein autophosphorylation, ephrin receptor signaling pathway, negative regulation of axon regeneration, regulation of astrocyte differentiation, regulation of axonogenesis, positive regulation of dendrite morphogenesis, protein stabilization, regulation of dendritic spine morphogenesis, positive regulation of protein tyrosine kinase activity, negative regulation of ERK1 and ERK2 cascade, nephric duct morphogenesis, cochlea development, fasciculation of sensory neuron axon, fasciculation of motor neuron axon, neuron projection guidance, synapse disassembly, negative regulation of cellular response to hypoxia, negative regulation of long-term synaptic potentiation, positive regulation of beta-amyloid formation, positive regulation of

		1			aspartic-type endopeptidase activity involved in amyloid precursor protein catabolic process, negative regulation of proteolysis involved in cellular protein catabolic process, cellular response to beta-amyloid, regulation of modification of synaptic structure, regulation of synapse disassembly, positive regulation of Rho guanyl-nucleotide exchange factor activity.
TACSTD2	tumor associated calcium signal transducer 2	1	2.039312773	0.005247	Visual perception, negative regulation of epithelial cell migration, regulation of epithelial cell proliferation, response to stimulus, negative regulation of stress fiber assembly, ureteric bud morphogenesis, negative regulation of branching involved in ureteric bud morphogenesis, negative regulation of substrate adhesion-dependent cell spreading, negative regulation of ruffle assembly, negative regulation of cell motility, positive regulation of stem cell differentiation.

GJA1	gap junction protein alpha 1	6	2.03828733	0.004069	Establishment of mitotic spindle orientation, osteoblast differentiation, in utero embryonic development, neuron migration, heart looping, epithelial cell maturation, lens development in camera-type eye, cardiac conduction system development, atrial ventricular junction remodeling, signal transduction, cell-cell signaling, spermatogenesis, heart development, adult heart development, protein localization, male gonad development, positive regulation of gene expression, negative regulation of gene expression, cell communication by chemical coupling, cell communication by electrical coupling, glutamate secretion, gap junction assembly, negative regulation of cell growth, regulation of bone mineralization, negative regulation of gonadotropin secretion, ion transmembrane transport, cellular protein localization, glutathione transmembrane transport, maintenance of permeability of blood-brain barrier, T cell proliferation, embryonic digit morphogenesis, xenobiotic transport, positive regulation of I-kappaB kinase/NF-kappaB signaling, skeletal muscle tissue regeneration, cell-cell junction organization, positive regulation of striated muscle tissue development, bone remodeling, regulation of bone remodeling, blood vessel morphogenesis, milk ejection reflex, regulation of ventricular cardiac muscle cell membrane repolarization, bone development, regulation of atrial cardiac muscle cell membrane depolarization,

					regulation of ventricular cardiac muscle cell membrane depolarization, cardiac conduction, cellular response to pH, atrial cardiac muscle cell action potential, cell communication by electrical coupling involved in cardiac conduction, microtubule-based transport, negative regulation of trophoblast cell migration, cellular response to beta-amyloid, positive regulation of vascular smooth muscle cell proliferation, positive regulation of morphogenesis of an epithelium, positive regulation of mesodermal cell differentiation, positive regulation of stem cell proliferation.
AC126755.1	nuclear pore complex interacting protein family member A5	16	2.014705542	0.000976	Unknown function.

JUP	junction plakoglobin	17	2.0096818 92	0.00186 4	Positive regulation of cell-matrix adhesion, desmosome assembly, cell adhesion, cell migration, regulation of cell proliferation, positive regulation of protein import into nucleus, negative regulation of blood vessel endothelial cell migration, skin development, positive regulation of angiogenesis, positive regulation of transcription from RNA polymerase II promoter, detection of mechanical stimulus, positive regulation of sequence-specific DNA binding transcription factor activity, canonical Wnt signaling pathway, endothelial cell-cell adhesion, cellular response to indole-3-methanol, protein localization to plasma membrane, bundle of His cell-Purkinje myocyte adhesion involved in cell communication, regulation of heart rate by cardiac conduction, positive regulation of canonical Wnt signaling pathway, cell-cell adhesion, regulation of ventricular cardiac muscle cell action potential.
NECTIN1	nectin cell adhesion molecule 1	11	1.9735708 46	0.00127 8	Lens morphogenesis in camera-type eye, desmosome organization, iron ion transport, immune response, cell adhesion, homophilic cell adhesion via plasma membrane adhesion molecules, heterophilic cell-cell adhesion via plasma membrane cell adhesion molecules, axon guidance, virion attachment to host cell, viral entry into host cell, regulation of synapse assembly, retina development in camera-type eye, enamel mineralization, cell-cell adhesion, protein localization to cell junction.

GTF2IP12	general transcription factor IIi pseudogene 12	4	1.930873976	0.040077	Unknown function.
IFFO2	intermediate filament family orphan 2	1	1.928865469	0.006698	Unknown function.
AGAP9	ArfGAP with GTPase domain, ankyrin repeat and PH domain 9	10	1.925946641	0.003965	Activation of GTPase activity.
GEMIN4	gem nuclear organelle associated protein 4	17	1.914798019	0.002184	Spliceosomal snRNP assembly, rRNA processing.
HRNR	hornerin	1	1.901702643	0.002064	Keratinization, cell envelope organization, establishment of skin barrier.

BAIAP2	BAI1 associated protein 2	17	1.898719565	0.002761	Plasma membrane organization, axonogenesis, brain development, insulin receptor signaling pathway, regulation of cell shape, response to bacterium, dendrite development, positive regulation of actin filament polymerization, regulation of actin cytoskeleton organization, protein localization to synapse, regulation of synaptic plasticity, actin filament bundle assembly, actin crosslink formation, positive regulation of dendritic spine morphogenesis, cellular response to epidermal growth factor stimulus, cell-cell adhesion, modification of synaptic structure, modulating synaptic transmission, cellular response to L-glutamate, regulation of modification of postsynaptic actin cytoskeleton, positive regulation of actin cytoskeleton reorganization, positive regulation of excitatory postsynaptic potential.

EPHB3	EPH receptor B3	3	1.896693519	0.001377	Angiogenesis, urogenital system development, protein phosphorylation, transmembrane receptor protein tyrosine kinase signaling pathway, axon guidance, axonal fasciculation, cell migration, central nervous system projection neuron axonogenesis, corpus callosum development, regulation of cell-cell adhesion, retinal ganglion cell axon guidance, positive regulation of kinase activity, substrate adhesion-dependent cell spreading, regulation of GTPase activity, protein autophosphorylation, ephrin receptor signaling pathway, thymus development, digestive tract morphogenesis, regulation of axonogenesis, positive regulation of synapse assembly, palate development, dendritic spine development, dendritic spine morphogenesis.
PROM2	prominin 2	2	1.890694961	0.005106	Positive regulation of protein phosphorylation, positive regulation of cell projection organization, regulation of GTPase activity, negative regulation of pinocytosis, negative regulation of caveolin-mediated endocytosis.
LAD1	ladinin 1	1	1.880272841	0.006687	Unknown function.
DSG3	desmoglein 3	18	1.879574778	0.010614	Homophilic cell adhesion via plasma membrane adhesion molecules, cell-cell adhesion.
PKP3	plakophilin 3	11	1.851235539	0.005228	Desmosome assembly, cell-cell junction assembly, positive regulation of gene expression, protein localization to plasma membrane, cell-cell adhesion, negative regulation of mRNA catabolic process.

PTPRZ1	protein tyrosine phosphatase, receptor type Z1	7	1.846820454	0.023057	Hematopoietic progenitor cell differentiation, protein dephosphorylation, axonogenesis, central nervous system development, learning or memory, regulation of myelination, peptidyl-tyrosine dephosphorylation, negative regulation of neuron apoptotic process, oligodendrocyte differentiation, positive regulation of oligodendrocyte differentiation, regulation of oligodendrocyte progenitor proliferation.
NOTCH3	notch 3	19	1.836998901	0.001481	Negative regulation of transcription from RNA polymerase II promoter, Notch signaling pathway, axon guidance, neuroblast differentiation, forebrain development, negative regulation of neuron differentiation, positive regulation of transcription from RNA polymerase II promoter, positive regulation of smooth muscle cell proliferation, neuron fate commitment, artery morphogenesis, glomerular capillary formation.
AC009533.1	*pseudogene*	12	1.832185439	0.001377	Unknown function.

Table S8. Top 50 Upregulated Genes in Fibrotic LS *versus* HC using overlap. *P-values were corrected by multiple hypothesis testing to give the Q value by the Benjamini-Hochberg method. **Functional Annotation is adapted from Database for Annotation, Visualization and Integrated Discovery (DAVID). Chr; chromosome.

Gene	Protein Encoded	Chr.	LogFC	Q value*	**Functional Annotation
AC006064.5	*pseudogene*	12	-5.377019797	0.045950	Unknown function.
AMY1B	amylase, alpha 1B (salivary)	1	-3.762244731	0.011029	Carbohydrate metabolic process, oligosaccharide metabolic process.
AMY2A	amylase, alpha 2A (pancreatic)	1	-3.666555153	0.025834	Carbohydrate metabolic process, carbohydrate catabolic process, polysaccharide digestion.
POTEKP	POTE ankyrin domain family member K, pseudogene	2	-3.560209032	0.026354	Axonogenesis, cell motility, postsynaptic actin cytoskeleton organization.
UBBP4	ubiquitin B pseudogene 4	17	-3.307154771	0.013555	Unknown function.
ADH1C	alcohol dehydrogenase 1C (class I), gamma polypeptide	4	-3.12622025	0.039841	Ethanol oxidation, retinol metabolic process, retinoic acid metabolic process.
AC078927.1	*pseudogene*	12	-3.094384721	0.026929	Unknown function.
FP671120.3	*pseudogene*	21	-3.006002094	0.001465	Unknown function.
FP236383.3	*pseudogene*	21	-2.953596868	0.001297	Unknown function.
ACTG1P14	actin gamma 1 pseudogene 14	9	-2.831497634	0.044198	Unknown function.
POTEI	POTE ankyrin domain family member I	2	-2.738894476	0.035795	Retina homeostasis, axonogenesis, cell motility, postsynaptic actin cytoskeleton organization.
TSPAN8	tetraspanin 8	12	-2.720942208	0.017468	Spermatogenesis, regulation of gene expression, negative regulation of blood coagulation.
FTH1P16	ferritin heavy chain 1 pseudogene 16	11	-2.56947263	0.000465	Unknown function.

MT-ATP8	mitochondrially encoded ATP synthase membrane subunit 8	M	-2.558360766	0.039802	ATP synthesis coupled proton transport, mitochondrial ATP synthesis coupled proton transport, hydrogen ion transmembrane transport.
MT-ND4L	mitochondrially encoded NADH:ubiquinone oxidoreductase core subunit 4L	M	-2.535946832	0.025994	Mitochondrial electron transport, NADH to ubiquinone, aerobic respiration, ATP synthesis coupled electron transport, mitochondrial ATP synthesis coupled proton transport.
MT-ND1	mitochondrially encoded NADH:ubiquinone oxidoreductase core subunit 1	M	-2.381448887	0.027884	Mitochondrial electron transport, NADH to ubiquinone, aerobic respiration, mitochondrial respiratory chain complex I assembly, mitochondrial ATP synthesis coupled proton transport.
FTH1P5	ferritin heavy chain 1 pseudogene 5	6	-2.340789929	0.003227	Unknown function.
RN7SL1	RNA, 7SL, cytoplasmic 1	14	-2.338791824	0.001028	SRP-dependent cotranslational protein targeting to membrane, signal sequence recognition.
CAMK2N1	calcium/calmodulin dependent protein kinase II inhibitor 1	1	-2.320289093	0.000610	Negative regulation of protein kinase activity, long-term memory, positive regulation of inflammatory response.

GREM1	gremlin 1, DAN family BMP antagonist	15	-2.319254012	0.001028	Cell morphogenesis, sprouting angiogenesis, transcription regulation in heart and kidney development, signal transduction, cell proliferation, limb and organ morphogenesis, bone and cartilage growth regulation, angiogenesis, DNA transcription, and cell differentiation. It also involves various negative regulations in bone remodeling and Wnt signaling, and positive influences on NF-kappaB activity, telomerase activity, and cardiac muscle development.
MT-ND3	mitochondrially encoded NADH:ubiquinone oxidoreductase core subunit 3	M	-2.296709396	0.034145	Mitochondrial electron transport, NADH to ubiquinone, response to oxidative stress, aerobic respiration, response to light intensity, mitochondrial ATP synthesis coupled proton transport, cellular response to glucocorticoid stimulus.
ASS1P9	argininosuccinate synthetase 1 pseudogene 9	5	-2.296412265	0.014078	Unknown function.
PPIAP19	peptidylprolyl isomerase A pseudogene 19	10	-2.29417462	0.034711	Unknown function.
MTATP6P1	mitochondrially encoded ATP synthase 6 pseudogene 1	1	-2.294076253	0.011281	Unknown function.
AC133435.1	*pseudogene*	3	-2.284065598	0.022259	Unknown function.
AL139099.4	*pseudogene*	14	-2.268492108	0.001028	Unknown function.

FP671120.4	*pseudogene*	21	-2.24873022	0.034711	Unknown function.
ATRNL1	attractin like 1	10	-2.175585014	0.003907	G-protein coupled receptor signaling pathway, animal organ morphogenesis, tissue development, cell migration, substrate adhesion-dependent cell spreading.
GSTM5	glutathione S-transferase mu 5	1	-2.156081123	0.018020	Glutathione metabolic process, glutathione derivative biosynthetic process.
AC092017.1	*pseudogene*	1	-2.154146357	0.008292	Unknown function.
MT-ND2	mitochondrially encoded NADH:ubiquinone oxidoreductase core subunit 2	M	-2.141351911	0.033624	Mitochondrial electron transport, NADH to ubiquinone, aerobic respiration, mitochondrial respiratory chain complex I assembly, mitochondrial ATP synthesis coupled proton transport, reactive oxygen species metabolic process.
ALDH1B1	aldehyde dehydrogenase 1 family member B1	9	-2.123125104	0.001824	Carbohydrate metabolic process, ethanol catabolic process.
MT-ATP6	mitochondrially encoded ATP synthase membrane subunit 6	M	-2.118479188	0.034601	ATP biosynthetic process, ion transport, aging, ATP synthesis coupled proton transport, mitochondrial ATP synthesis coupled proton transport, response to hyperoxia, hydrogen ion transmembrane transport.

MT-CO3	mitochondrially encoded cytochrome c oxidase III	M	-2.098789923	0.024656	Mitochondrial electron transport, cytochrome c to oxygen, respiratory chain complex IV assembly, aerobic respiration, aerobic electron transport chain, cellular respiration, hydrogen ion transmembrane transport.
OGN	osteoglycin	9	-2.086254337	0.006782	Signal transduction, negative regulation of smooth muscle cell proliferation, bone development.
MT-CYB	mitochondrially encoded cytochrome b	M	-2.083627284	0.035557	Oxidative phosphorylation, mitochondrial electron transport, ubiquinol to cytochrome c, respiratory electron transport chain, cellular respiration, hydrogen ion transmembrane transport.
MT-CO1	mitochondrially encoded cytochrome c oxidase I	M	-2.081977809	0.021141	Oxidative phosphorylation, mitochondrial electron transport, cytochrome c to oxygen, response to oxidative stress, aging, aerobic respiration, electron transport coupled proton transport, cerebellum development, respiratory electron transport chain, cellular respiration, response to copper ion, response to electrical stimulus.

MT-CO2	mitochondrially encoded cytochrome c oxidase II	M	-2.081029888	0.032286	Mitochondrial electron transport, cytochrome c to oxygen, lactation, response to cold, positive regulation of hydrogen peroxide biosynthetic process, positive regulation of necrotic cell death, ATP synthesis coupled electron transport, cellular respiration, hydrogen ion transmembrane transport, positive regulation of ATP biosynthetic process.
MUC3A	mucin 3A, cell surface associated	7	-2.062091453	0.040769	Unknown function.
AC093484.3	*pseudogene*	17	-2.044628861	0.028391	Unknown function.
ACTG2	actin, gamma 2, smooth muscle, enteric	2	-2.024511946	0.007998	Positive regulation of gene expression, mesenchyme migration.
AC104651.1	*pseudogene*	2	-2.024405977	0.003122	Unknown function.
MT-ND4	mitochondrially encoded NADH:ubiquinone oxidoreductase core subunit 4	M	-1.977764583	0.035165	Response to hypoxia, in utero embryonic development, mitochondrial electron transport, NADH to ubiquinone, aging, aerobic respiration, electron transport coupled proton transport, cerebellum development, mitochondrial respiratory chain complex I assembly, response to nicotine, ATP synthesis coupled electron transport, mitochondrial ATP synthesis coupled proton transport, response to ethanol.

GALNT12	polypeptide N-acetylgalactosaminyltransferase 12	9	-1.966139246	0.010967	Protein O-linked glycosylation, O-glycan processing.
MT-RNR1	mitochondrially encoded 12S RNA	M	-1.95126754	0.023594	Osteoblast differentiation, regulation of transcription from RNA polymerase II promoter, activation of protein kinase activity, osteoblast proliferation, regulation of carbohydrate utilization, skeletal muscle tissue growth, positive regulation of protein serine/threonine kinase activity, purine-containing compound biosynthetic process, negative regulation of phosphatidylinositol-3,4,5-trisphosphate 5-phosphatase activity.
TFF3	trefoil factor 3	21	-1.908159834	0.037148	Regulation of glucose metabolic process, maintenance of gastrointestinal epithelium.
ETHE1	ETHE1, persulfide dioxygenase	19	-1.88685121	0.011757	Glutathione metabolic process, hydrogen sulfide metabolic process.

NR5A2	nuclear receptor subfamily 5 group A member 2	1	-1.844932599	0.014219	Regulation of transcription, DNA-templated, regulation of transcription from RNA polymerase II promoter, bile acid metabolic process, hormone-mediated signaling pathway, embryo development ending in birth or egg hatching, tissue development, intracellular receptor signaling pathway, epithelial cell differentiation, regulation of cell proliferation, homeostatic process, cholesterol homeostasis, positive regulation of viral genome replication, positive regulation of transcription, DNA-templated, positive regulation of transcription from RNA polymerase II promoter, pancreas morphogenesis, acinar cell differentiation, calcineurin-mediated signaling, cellular response to leukemia inhibitory factor.
SNORD3B-2	small nucleolar RNA, C/D box 3B-2	17	-1.818591807	0.015388	Ribosome biogenesis in eukaryotes.
FUCA1	alpha-L-fucosidase 1	1	-1.817313405	0.000557	Glycosaminoglycan catabolic process, glycoside catabolic process, glycolipid catabolic process.

Table S9. Top 50 Downregulated Genes in Fibrotic LS *versus* HC using overlap. *P-values were corrected by multiple hypothesis testing to give the Q value by the Benjamini-Hochberg method. **Functional Annotation is adapted from Database for Annotation, Visualization and Integrated Discovery (DAVID). Chr; chromosome.

Condition	Number of Samples	Fusion Events (per sample)
Active LS	19	282.1
Inactive LS	9	245.1
HC	9	189.6

Table S10. Fusion Events per LS Sample.

Patient	Condition 1	Condition2	Gene 1	Gene 2
Patient A	HC	N/A	MIER1	TSC22D3
	HC	N/A	EEF2K	LSM8
Patient A	Inactive	Generalized	EHBP1	ITGB1
Patient B	Active	Circumscribed	RUNX1	WASHC4
Patient C	Active	Circumscribed	MAP3K4	WASF3
Patient D	Active	Circumscribed	PLCB1	TMX4
Patient E	Active	Generalized	SP140L	ORC4
Patient F	Inactive	Generalized	JAZF1	TAX1BP1

Table S11. Fusion Genes identified in pediatric LS samples.Gene fusions identified in HC and LS samples. Fusions were detected using either STARFUSION or ARRIBA software with statistical significance at $p<0.05$.
